# Combination of mutations in genes controlling DNA repair and high mutational load plays a prognostic role in pancreatic ductal adenocarcinoma (PDAC): a retrospective real-life study in Sardinian population

**DOI:** 10.1186/s12967-024-04923-3

**Published:** 2024-01-27

**Authors:** Maria Cristina Sini, Maria Grazia Doro, Laura Frogheri, Angelo Zinellu, Panagiotis Paliogiannis, Alberto Porcu, Fabrizio Scognamillo, Daniele Delogu, Davide Adriano Santeufemia, Ivana Persico, Grazia Palomba, Giovanni Battista Maestrale, Antonio Cossu, Giuseppe Palmieri

**Affiliations:** 1 Unit of Cancer Genetics, Institute of Genetic Biomedical Research (IRGB), National Research Council (CNR), Sassari, Italy; 2 Department of Biomedical Sciences, University of Sassari, Sassari, Italy; 3 Department of Medicine, Surgery and Pharmacy, University of Sassari, Traversa La Crucca 3, 07100 Sassari, Italy; 4 Oncology, Civil Hospital, Alghero, Italy; 5 Immuno-Oncology & Targeted Cancer Biotherapies, University of Sassari, Sassari, Italy

**Keywords:** Pancreatic ductal adenocarcinoma, Mutation analysis, Genome damage repair, Mutation load rate, Prognosis

## Abstract

**Background:**

Patients with pancreatic ductal adenocarcinoma (PDCA) carrying impaired mismatch repair mechanisms seem to have an outcome advantage under treatment with conventional chemotherapy, whereas the role for the tumor mutation burden on prognosis is controversial. In this study, we evaluated the prognostic role of the mutated genes involved in genome damage repair in a real-life series of PDAC patients in a hospital-based manner from the main Institution deputed to surgically treat such a disease in North Sardinia.

**Methods:**

A cohort of fifty-five consecutive PDAC patients with potentially resectable/border line resectable PDAC (stage IIB-III) or oligometastatic disease (stage IV) and tumor tissue availability underwent next-generation sequencing (NGS)-based analysis using a panel containing driver oncogenes and tumor suppressor genes as well as genes controlling DNA repair mechanisms.

**Results:**

Genes involved in the both genome damage repair (DR) and DNA mismatch repair (MMR) were found mutated in 17 (31%) and 15 (27%) cases, respectively. One fourth of PDAC cases (14/55; 25.5%) carried tumors presenting a combination of mutations in repair genes (DR and MMR) and the highest mutation load rates (MLR-H). After correction for confounders (surgery, adjuvant therapy, stage T, and metastasis), multivariate Cox regression analysis indicated that mutations in DR genes (HR = 3.0126, 95% CI 1.0707 to 8.4764, p = 0.0367) and the MLR (HR = 1.0018, 95%CI 1.0005 to 1.0032, p = 0.009) were significantly related to worse survival.

**Conclusions:**

The combination of mutated repair genes and MLR-H, which is associated with a worse survival in our series of PDAC patients treated with conventional chemotherapy protocols, might become a predictive biomarker of response to immunotherapy in addition to its prognostic role in predicting survival.

**Supplementary Information:**

The online version contains supplementary material available at 10.1186/s12967-024-04923-3.

## Introduction

Pancreatic ductal adenocarcinoma (PDAC) is a highly lethal disease, with an estimated annual incidence of 62,210 cases and 49,830 related deaths in 2022 in the United States [1]. Although, complete surgical resection (R0 resection) represents the only potentially curative treatment, only 15%-20% of pancreatic cancer patients undergo primary tumor resection since cancer early spreads to other organs and/or peripancreatic vessels, limiting its role [2]. Overall, the introduction of new surgical techniques and medical therapies such as laparoscopic techniques and neo-adjuvant chemo-radiotherapy has only led to modest improvements in outcomes [3].

Unfortunately, due to lack of early diagnostic tools and initial specific symptoms, up to 80% of patients presents with either unresectable or metastatic disease and a 5-year relative survival rate of approximately 11.5% [4, 5]. Moreover, prognosis remains poor also for patients who are diagnosed with localized tumor and who are treated by potentially curative surgery [4, 5] because of a high rate of either, local or, distant disease recurrence despite next adjuvant treatment such as chemotherapy or chemo-radiotherapy [6–9].

Palliative systemic chemotherapy represents the standard treatment of advanced PDAC; it is delivered with the aim to improve patient’s symptoms, quality of life, and survival. There are well-established options for first-line advanced PDAC treatment with the choice depending on the patient’s Eastern Cooperative Oncology Group (ECOG) performance status, comorbidity, and patient’s preferences. Usually, these options include combination chemotherapy regimens such as FOLFIRINOX (oxaliplatin, irinotecan, leucovorin, and 5-fluorouracil) [10] and gemcitabine plus nab-paclitaxel [11] when the patient’s performance status is good. For patients with poorer performance status or older age, gemcitabine single-agent treatment represents a valid option [12].

Despite the introduction in clinical practice of new chemotherapeutic schedules [10, 11] and drugs [13], only little progresses have been made in recent years. This may be potentially due to the diversity of pancreatic cancer genomic landscape, identifying subgroups of patients with distinct biological and clinical tumor behavior [14, 15]. Considering all driver alterations in primary and metastatic tumors detected by whole-genome sequencing, pancreatic neuroendocrine carcinomas have been reported to show a significantly intense transformation of the genomic landscape during tumor progression, whereas PDACs belong to a large group of cancer types displaying variable genomic differences within a substantially conserved portrait of gene driver alterations [15].

Immune checkpoint inhibitors (ICI) as monoclonal antibodies (mAbs) against the programmed death-1 (PD-1)/programmed death- ligand 1 (PD-L1) axis are particularly active in gastrointestinal cancers with deficient mismatch repair (dMMR) system or high microsatellite instability (MSI-H) [16–18]. However, there are few data on the use of checkpoint inhibitors in MSI-H/dMMR mPDAC. Marabelle et al. [16] investigated treatment with the anti-PD-1 Pembrolizumab in a cohort of MSI-H patients suffering from metastatic PDAC, which were enrolled in the Keynote 158-Study. Among 22 patients, an ORR of 18.2% was reported. Median PFS was 2.1 months while median OS was 4 months. Interestingly, patients responding to the treatment exhibited a longer duration of response (13.4 months), though only a limited subgroup of patients with MSI-H/dMMR appears to substantially benefit from an ICI treatment [16].

Previous studies showed that PDCA patients carrying a proficient MMR (pMMR) system and treated with conventional chemotherapy have a survival advantage compared to dMMR patients, suggesting that MSI status can be considered a potential predictor of sensitivity to different treatments [19–21].

Independently from MSI-H/dMMR, the role of high tumor mutational burden (TMB-H) in selecting patients to be addressed to ICI treatment remains largely unclear, though preliminary data suggests a possible role of immunotherapy in selected cases of PDAC [22, 23]. In a retrospective cohort study, the TMB-H—which was found in a tiny fraction (3.3%) among 48.606 gastrointestinal tumors—was not able to predict antitumor immune response due to a poor correlation with the infiltration of immune cells and immune signatures within the tumor environment [24].

In another retrospective analysis, about one fourth of 1.856 patients with pancreatic cancer were found to carry actionable molecular alterations and among those who received a matched therapy a significantly longer overall survival was reported as compared with patients without a druggable targets [25]. This is consistent with observation that PARP inhibitor Olaparib does improve progression-free survival when used as a maintenance therapy in the subgroup of patients with metastatic PDAC and a BRCA1-2 germline mutation, that benefits from platinum-based chemotherapy [26]. Such evidence represented a clear indication that the BRCA1-2 mutational status should be routinely investigated in metastatic PDAC—though the expected occurrence of mutations in these genes for this malignancy is lower than 4% (any BRCA mutation: 3.3%; 1.7% to 5.3% [27]) within the Caucasian/white population.

Therefore, an increasingly extensive use of the molecular classification of the advanced PDAC patients seems to become mandatory to tailor treatment decision in the context of the management of this highly malignant neoplasm. In other words, characterization of the mutation genomic landscape may offer chance to improve the management and outcome of PDAC patients with non-localized disease. The aim of the current study is to provide additional clues about the prognostic role of the main molecular alterations in a real-life series of N + PDAC patients molecularly classified in a hospital-based manner diagnosed at the main Institution deputed to surgically treat patients with pancreatic cancer across the entire North Sardinia.

## Methods

### Samples

A cohort of fifty-five consecutive patients with a histologically proven diagnosis of PDAC at disease stage IIB (T1-3, N1, M0) or higher stage (III-IV)—according to the American Joint Commission on Cancer (AJCC)/TNM 2017, eighth edition, guidelines [28]—and availability of primary tissue samples was retrieved from the archives of the Anatomic Pathology Institute of the University of Sassari, during a period of time from July 2014 to June 2020 in order to have a follow-up coverage of at least 3 years. Demographic, clinical and pathological data of all patients were obtained from clinical records and histology reports exclusively for the purposes of the study. At the time of diagnosis, all patients gave their informed consent for the use of their archival samples and clinical data for the purposes of translational researches and studies, in an anonymous way. The study was performed in accordance with the principles of the declaration of Helsinki and was approved by the Committee for the Ethics of the Research and Bioethics of the National Research Council (CNR).

### Mutation analysis

For mutation analysis, DNA was extracted from formalin-fixed paraffin-embedded (FFPE) primary PDAC tumors using the GeneRead DNA FFPE tissue Kit (QIAGEN, Hilden, Germany). Briefly, FFPE blocks were firstly sectioned to permit adequate morphologic assessment by hematoxylin and eosin staining and then, using light microscopy, tissue sections underwent manual macrodissection by removing surrounding healthy tissues in order to obtain tumor samples with a minimum of 50% of neoplastic cells (range 50%-80%). Isolated genomic DNA and correspondent libraries were accurately quantified using fluorescence-based quantification method such as Qubit dsDNA HS (Invitrogen, Life Technologies, USA) in order to minimize technical problems due to the insufficient DNA sample quality.

Next Generation Sequencing (NGS) analysis was performed on Ion S5 GeneStudio (Thermo Fisher Scientific, Waltham, USA), using a customized Oncomine™ Tumor specific gene panel requiring 10 ng DNA/Pool consisting of two primer pools (1777 amplicons; amplicon range: 125-175 bp) covering hot spot or entire coding regions of 40 oncogenic driver genes, including the main ones involved at different impact level in tumor onset and progression (*KRAS*, *NRAS*, *BRAF*, *SMAD4*, *TP53*, *PIK3CA*, *PTEN*, *AKT3*, *CDKN2A*, *CDK4*, *RB1*, *CTNNB1*, *APC*, *FGFR2*, *KIT*, *EGFR*, *ALK*, *GNAQ*/*11*, *NF1*, *MYC*, *VHL*) as well as in both genome damage repair by homologous recombination (*ARID1/2*, *ATM*, *BAP1*, *BARD1*, *BRIP1*, *CDH1*, *CHEK2*, *FANCD2*, *PALB2*, *POLE*, *POLD1*, *RAD51C*/*D*) and DNA mismatch repair (*MLH1*, *MSH2*, *MSH3*, *MSH6*, *PMS2*) mechanisms. About two thirds of PDCA cases (38/55; 69%) were previously found negative for mutations in both BRCA1 and BRCA2 genes, using a specific panel (Oncomine BRCA Assay, Thermo Fisher Scientific); in the remaining cases, mutational status of BRCA1-2 was not determined for low quality NGS analyses within these large and complex genomic loci.

The annotation and interpretation of all identified variants was performed using Ion Reporter™ Software (Thermo Fisher Scientific) a suite of bioinformatic tools that performs analysis on BAM files that are output from Torrent Suite™ Software. Output files result from the use of a specific VariantCaller plugin in Ion Reporter™ Software that provided a final annotated analysis file.

Considering the poor quality of the isolated DNA from archived tissue samples of our series (majority of them were sampled ≥ 5 years before)—thus affecting the NGS library quality, coverage of 100 reads or greater (≥ 100) and variant allele frequency (VAF) cutoff > 5%, anyway with at least 10 mutated alleles for each candidate amplicon, were adopted for mutation selection criteria.

Variants were screened against COSMIC v92 database (Catalogue of Somatic Mutations in Cancer; https://cancer.sanger.ac.uk/cosmic) and VARSOME (https://varsome.com/) to identify known somatic mutations and mutation types, respectively. The clinical significance of all identified variants was examined using the standards and guidelines for the interpretation of sequence variants recommended by American College of Medical Genetics and Genomics (ACMG Laboratory Quality Assurance Committee) and Association for Molecular Pathology (AMP).

Mutational load rate (MLR) estimates were directly computed by the Ion Reporter™ Software to nearly all (50/55; 91%) cases using the specific panel analysis workflow including only non-synonymous somatic mutations (ns-SNVs/indel) in coding regions, with computational germline status filtering. Limit of detection of variant minimum allele frequency was set to ≥ 10% (LOD10) since DNA sequence variants with lower allele frequencies are difficult to be reliably quantitated and classified. Considering that a NGS panel including a limited number of genes—such as the one we used in the present study (Oncomine™ Tumor specific gene panel)—is not able to produce a canonical tumor mutation burden (TMB) value, MLR can be estimated by targeting a group of only a few hundred genes of interest with average coverage of 120X to 150X, but it can be performed at a high depth even for the smaller panels as mutational load rate (MLR/0.17 Mb). To calculate the MLR for our panel, cutoff values were defined as MLR-very high (≥ 75 mutations), MLR-high (> 25 ≤ 75 mutations), MLR-medium (> 10 ≤ 25 mutations) and MLR-low (≤ 10 mutations).

The panel was designed to characterize tumors for which the presence of predisposing mutations in *BRCA1* and *BRCA2* was not required since these two genes were examined apart (see above); the panel was instead aimed at assessing the presence of “BRCAness” characterized by the occurrence of pathogenic mutations affecting non-BRCA genes involved in the control of DNA repair by homologous recombination and determining a deficient genome damage repair (DR) mechanism.

### Microsatellite Instability (MSI)

Microsatellite instability (MSI) analysis was performed on all FFPE primary tumors of the series by Easy PGX System (Diatech Pharmacogenetics, Jesi, Italy) assays, regardless of the mutation status of the MMR genes. Easy PGX kits are designed to identify the detection of 8 mononucleotide "same-monomorphic" markers: BAT-25, BAT-26, NR-21, NR-22, NR-24, NR-27, CAT-25 and MONO-27 by real time PCR and subsequent analysis of the targets based on the denaturation profile. The test allows, accurately and with reduced "hands-on time", to detect the microsatellite instability in tumor samples without comparison with normal tissue.

The presence/absence of MSI was also confirmed with a second real-time PCR array, the fully automated IDYLLA™ (Biocartis, Mechelen, Belgium) test that showed a concordance of 100%.

### Copy number variation (CNV) analysis for KRAS gene

The Copy Number Variation (CNV) analysis was performed for the *KRAS* gene on all FFPE primary tumors by droplet digital PCR (ddPCR; Bio-Rad, Hercules, USA) assays, regardless of its mutational status. Each PCR reaction was carried out with 10 ng input DNA using QX200™ ddPCR (Bio-Rad) according to the manufacturer’s instructions. AP3B1 housekeeping gene was used as a reference. Droplets were generated using the QX200™ Droplet Generator. After PCR amplification, the fluorescence of each droplet was read on a QX200™ Droplet Reader and counted with QuantaSoft Software, version 1.7.4.0917. Using a modeling based on Poisson distribution, the software estimated the concentration of the target and reference gene in units of copies per microliters of amplified mix. An estimate of the target gene copy number was then determined by multiplying by 2 the ratio between the concentrations of the target and the reference gene.

### Statistical analysis

Survival probability for demographic, clinical, and genetic parameters was estimated using the Kaplan–Meier curves and log-rank test. The independent association between genetic mutations and survival was separately assessed for each genetic variable, by correcting for confounders that have a p < 0.1 in univariate analysis (surgery, adjuvant therapy, stage T, stage N and metastasis). Variables with p < 0.1 were retained in the model. Statistical analyses were performed using MedCalc for Windows, version 19.4.1 64 bit (MedCalc Software, Ostend, Belgium).

## Results

In our series, 63 consecutively collected patients with potentially resectable/border line resectable PDAC (stage IIB-III; N = 55) or stage IV disease (N = 8) and tumor tissue availability were identified. Criteria to classify potentially resectable and border line resectable PDAC were as summarized in Jain et al. 2023 [29]. Among them, eight patients were excluded because of the very low quality of the DNA extracted; the remaining 55 cases were included into the study and addressed to molecular screening. As shown in Table 1, a quite similar proportion of males and females (slightly preponderant into the latter ones) was present; median age was 69 years, range 41–85 years. The majority of patients (36/55; 65.5%) presented with IIB AJCC stage disease, whereas about one tenth of them (5/55; 9.1%) was found to carry a potentially resectable oligometastatic PDAC (all of them constituted by synchronous metastases involving a single liver segment with one case also carrying a single secondary lesion into the lung) (Table 1).

**Table 1:** Patients’ characteristics

Feature	No	%
*Sex*		
Female	29	52,7
Male	26	47,3
*AJCC Stage (TNM)*		
IIB (T1-3, pN1, M0)	36	65,45
III	14	25,45
(T1-3, pN2, M0)	11	20,0
(T4, any-N, M0)	3	5,5
IV (any-T, any-N, M1)	5	9,1
*First treatment*		
Neoadjuvant plus surgery	14	25,5
Neoadjuvant, no surgery	6	10,9
Surgery plus adjuvant	13	23,6
Surgery, no adjuvant	12	21,8
No surgery, no therapy	10	18,2
*1st line chemotherapy*		
None	29	52,7
Gemcitabine-based	20	36,4
Capecitabine	5	9,1
FOLFIRINOX	1	1,8

AJCC, American Joint Commission on Cancer; FOLFIRINOX regimen: 5-fluorouracil, leucovorin, irinotecan, and oxaliplatin

Considering the first treatment, more than two thirds of cases (39/55; 70.9) underwent the surgical excision with or without (neo-) adjuvant therapy (Table 1). In the remaining 16 patients, once the radiological resectability "status" was defined, surgery was excluded after taking into account the clinical context of the patient (age, associated pathologies, etc.) as well as after careful evaluation of the surgical and anesthesiological risk factors. Vast majority of patients receiving neoadjuvant therapies was treated with the FOLFIRINOX regimen (16/20; 80%), whereas the Gemcitabine plus NAB Paclitaxel combination was administered in the remaining one fifth of cases. The Gemcitabine therapy was used in all 16 patients who underwent the adjuvant treatment (in three cases, preceded by a neo-adjuvant therapy). At disease progression, nearly half of the patients (26/55; 47.3%) received a first line systemic chemotherapy (Table 1).

A total of 665 pathogenic/likely pathogenic variants were detected in the 55 lesions examined; the median value for total variants in our cases was 4 (range 1–61). Variants were classified as pathogenic or likely pathogenic in accordance with the criteria reported in COSMIC (FATHMM score > 0,70) and ClinVar databases. All the pathogenic/likely pathogenic variants detected are listed in Additional file 1: Table S1.

**Fig. 1:**
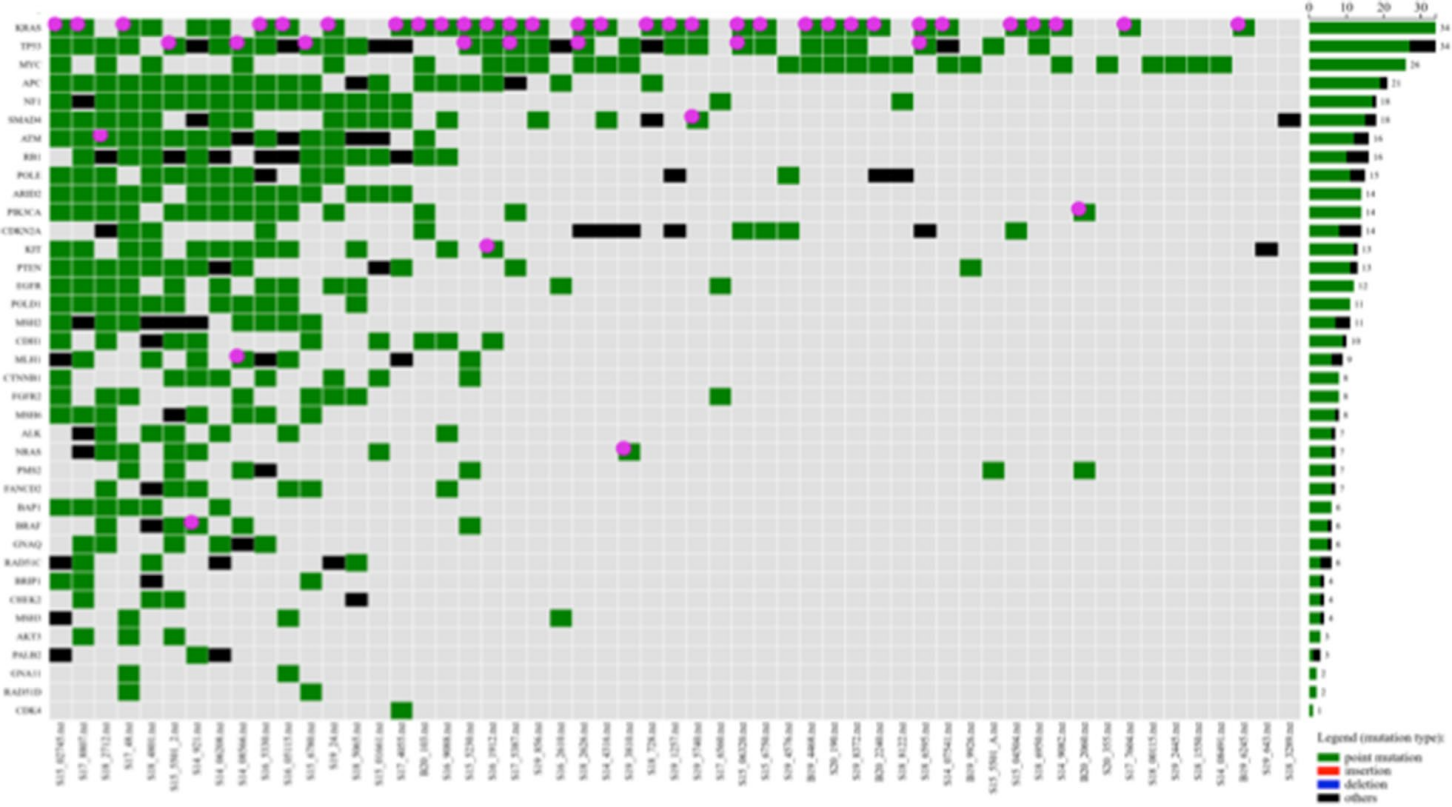
Oncoplot of somatic non-synonymous and pathogenic/likely pathogenic mutations. Total number of variants are reported non right side

Oncogenically activated *KRAS* was preponderantly involved in PDAC patients from our series, being mutations in this gene detected in 34/55 (62%) (Fig. 1; Additional file 2: Table S2). In particular, vast majority of *KRAS* mutations (28/34; 82%) occurs in codon 12 of exon 2, resulting in the replacement of glycine with aspartic acid (G12D: 19/28; 68%), valine (G12V: 6/28; 21%), and arginine (G12R: 3/28; 11%). Other substitutions were found in codon 61 of exon 3 involving different changes into the glutamine residue (Q61: 3/34; 9%) and in other distinct codons of exons 2 and 3 of *KRAS* (3/34; 9%). Considering that all mutations in *NRAS* (detected in 7 cases) and *BRAF* (in 6 cases) occurred within uncommonly involved codons—i.e. none in valine 600 residue for *BRAF* and none but one in G12/13 or Q61 codons for *NRAS*, the mutated *KRAS* acts as the true driver oncogene into the MAPK signaling pathway (Additional file 1: Table S1). Overall, 39 (71%) PDAC cases from our series carried mutation(s) in at least one of these three MAPK genes (Additional file 2: Table S2).

We then evaluated the potential correlation between the *KRAS* gene copy numbers (CNV) and the *KRAS* mutational status. Considering the *KRAS*-mutated cases, none of them showed co-occurrence of the *KRAS* gene amplification, even in PDAC samples with a mutation allele frequency above the median value of 11.6% (range, 5–49%).

The other mostly mutated genes (> 30% of cases) were: *TP53* (34/55; 62%), *MYC* (26; 47%), *APC* (21; 38%), *NF1* (18; 33%), and *SMAD4* (18; 33%). Considering the functional pathways, genes participating to the control of cell survival (*PTEN*, *PIK3CA*, and *AKT3*) and those involved into the regulation of the cell cycle progression (*CDKN2A*, *CDK4*, and *RB1*) were found mutated in 18 (33%) and 25 (45%) PDAC cases, respectively (Fig. 1**; **Additional file 1: Table S1). In our series, a lower rate of mutations (8/55; 15%) was found in *CTNNB1* gene and in nearly all cases they were associated with *APC* mutations; overall, these two genes acting downstream the WNT/β-catenin signaling pathway were found mutated in two fifths (22/55; 40%) of PDAC cases from our series (Fig. 1).

Genes involved in the both genome damage repair (DR) and DNA mismatch repair (MMR)—see Methods for the gene lists—were found mutated in 17 (31%) and 15 (27%) cases, respectively (Fig. 1**; **Additional file 2: Table S2).

The microsatellite instability (MSI) is mostly due to genetic or epigenetic impairment of at least one MMR gene with subsequent deficient DNA repair mechanisms. However, it is widely recognized that the most appropriate approach to assess the existence of a MSI status is represented by the detection of the instability effects on tumor genomic DNA. At genomic level, MSI is indeed characterized by small insertion or deletion within short tandem microsatellite repeats in tumor DNA. In our study, a real-time PCR-based high-resolution melting curve analysis using a specific assay with eight monomorphic homo-polymer biomarkers was performed for all DNA samples. Surprisingly, none of the 15 cases carrying mutations in MMR genes was found positive for high-MSI (MSI-H); the single sample carrying MSI-H was instead detected among the remaining 40 PDAC cases negative for mutated MMR genes (overall, 1/55; 1.8%).

**Fig. 2:**
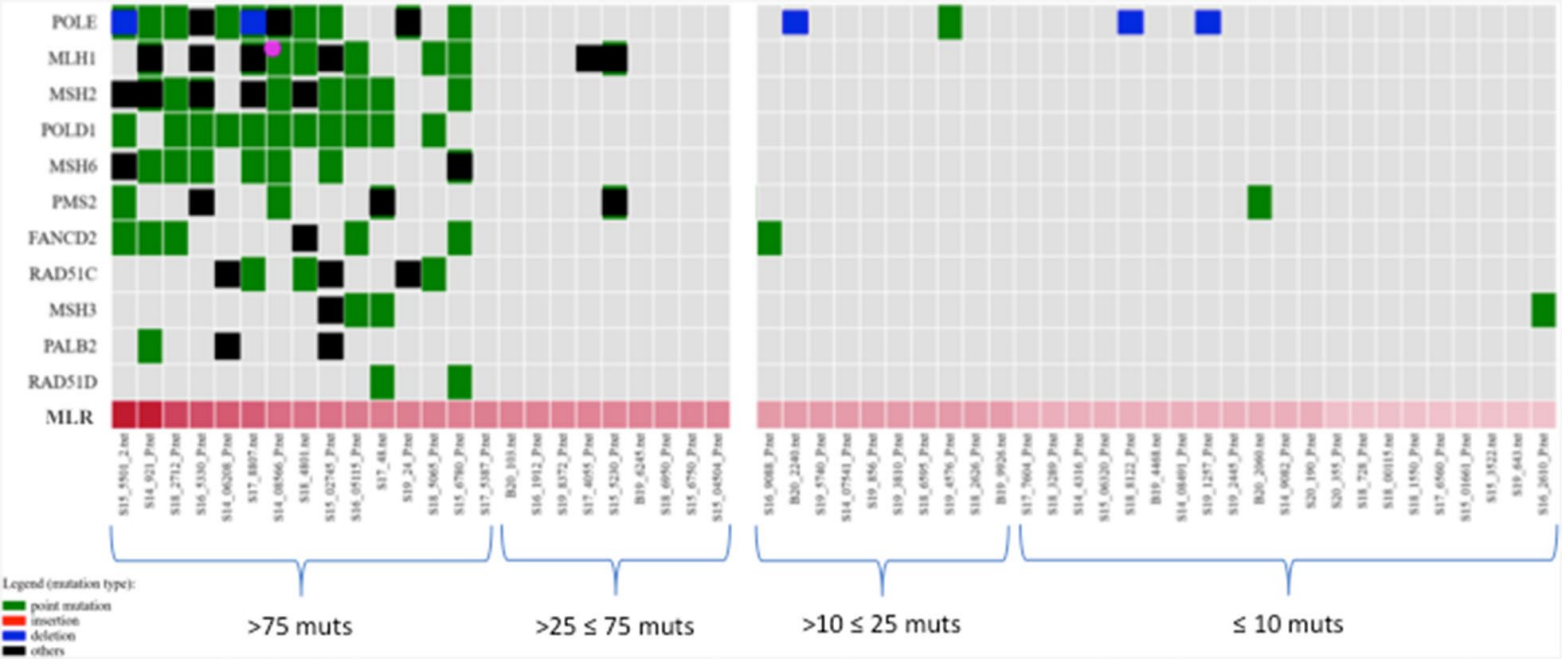
Oncoplot of somatic non-synonymous and pathogenic/likely pathogenic mutations in MMR and DR genes. Mutation load rate (MLR) values are indicated according to the total number of detected mutations

Finally, we evaluated the mutational load rate (MLR) in our series. Due to the limited genomic coverage of our NGS-based multi-gene array, MLR was calculated using a specific panel analysis workflow taking into consideration all non-synonymous somatic mutations in coding regions. The MLR cutoff values were defined as MLR-very high (≥ 75 mutations), MLR-high (> 25 and ≤ 75 mutations), MLR-medium (> 10 and ≤ 25 mutations) and MLR-low (≤ 10 mutations). When we compared the MLR classification with the gene mutational status, nearly all mutations in DR/MMR genes involved in DNA repair occurred in subset of PDAC cases with MLR-very high (Fig. 2).

Log-rank test by Kaplan–Meier survival curves, reported in Table 2 showed no significant association between genetic variables and survival. As reported in Table 3, some confounders (like surgery, adjuvant therapy, stage T, and metastasis) may however mask this association.

**Table 2:** Hazard ratios of the genetic mutations under investigation obtained by log-rank test

Mutated genes	HR	95% CI	p-value
*KRAS*	1.2337	0.5509 to 2.7627	0.610
*BRAF* (nonV600) *NRAS* (uncommon)	2.0720	0.7344 to 5.8458	0.170
*PIK3CA*	0.4734	0.2129 to 1.0525	0.066
*CDKN2A*	1.2823	0.5525 to 2.9763	0.560
*TP53*	0.4730	0.2084 to 1.0736	0.073
*SMAD4*	1.1467	0.5033 to 2.6130	0.740
*POLE* / *POLD1*	0.7953	0.3472 to 1.8213	0.590
MMR genes	0.8510	0.3763 to 1.9246	0.700
DR genes	0.8795	0.3999 to 1.9344	0.750
Mutation Load Rate	0.5953	0.2553 to 1.3878	0.230

HR, hazard ratio; CI, confidence interval; MMR, mismatch repair; DR, damage repair

**Table 3:** Hazard ratios of demographic and clinical parameters obtained by log-rank test

Characteristic	HR	95% CI	p-value
Age	1.7413	0.8202 to 3.6965	0.150
Sex	1.5123	0.7092 to 3.2250	0.280
Surgery	0.05810	0.01923 to 0.1755	** < 0.0001**
Adjuvant therapy	0.3638	0.1639 to 0.8076	**0.013**
Neo-adjuvant therapy	0.8056	0.3563 to 1.8213	0.600
First-line therapy	0.8185	0.3568 to 1.8773	0.630
T (T3-4 vs T1-2) stage	9.5881	1.4152 to 64.962	**0.026**
N (N2 vs N1) stage	2.3826	0.9539 to 5.9511	0.063
M stage (M1 vs M0)	266	28 to 2563	** < 0.0001**

In bold, significant values

HR, hazard ratio; CI, confidence interval

Subsequently, multivariate Cox regression models with backward elimination were built to evaluate independent association between the genetic alterations and mortality. As reported in Table 4, mutations in *SMAD4* (HR = 2.4987; 95% CI, 1.0134–6.1607, p = 0.0467) and DR genes (HR = 3.0126, 95% CI 1.0707 to 8.4764, p = 0.0367) as well as the MLR (HR = 1.0018, 95%CI 1.0005 to 1.0032, p = 0.009) were significantly related to survival after correction for the above reported confounders. A trend towards statistical significance was also observed with mutated MMR genes (HR = 2.5218; 95% CI, 0.8906–7.1406, p = 0.0815).

**Table 4:** Hazard ratios of the genetic alterations under investigation obtained by multivariate Cox regression models with backward elimination

Feature	HR	95% CI	p-value
Mutated (m)*KRAS*	–	–	–
Surgery	0.1238	0.0508 to 0.3020	< 0.0001
Adjuvant therapy	–	–	–
T	–	–	–
N	2.7091	1.1919 to 6.1575	0.0170
M	–	–	–
m*BRAF* (nonV600) m*NRAS* (uncommon)	–	–	–
Surgery	0.1238	0.0508 to 0.3020	< 0.0001
Adjuvant therapy	–	–	–
T	–	–	–
N	2.7091	1.1919 to 6.1575	0.0170
M	–	–	–
m*PIK3CA*	–	–	–
Surgery	0.1238	0.0508 to 0.3020	< 0.0001
Adjuvant therapy	–	–	–
T	–	–	–
N	2.7091	1.1919 to 6.1575	0.0170
M	–	–	–
m*CDKN2A*	–	–	–
Surgery	0.1238	0.0508 to 0.3020	< 0.0001
Adjuvant therapy	–	–	–
T	–	–	–
N	2.7091	1.1919 to 6.1575	0.1700
M	–	–	–
m*TP53*	–	–	–
Surgery	2.7091	1.1919 to 6.1575	< 0.0001
Adjuvant therapy	–	–	–
T	–	–	–
N	2.7091	1.1919 to 6.1575	0.1700
M	–	–	–
m*SMAD4*	2.4987	1.0134 to 6.1607	**0.0467**
Surgery	0.0891	0.0331 to 0.2397	< 0.0001
Adjuvant therapy	–	–	–
T	–	–	–
N	3.4631	1.4461 to 8.2934	0.0053
M	–	–	–
m*POLE* / m*POLD1*	–	–	–
Surgery	2.7091	1.1919 to 6.1575	< 0.0001
Adjuvant therapy	–	–	–
pT	–	–	–
pN	2.7091	1.1919 to 6.1575	0.1700
M	–	–	–
mMMR genes	2.5218	0.8906 to 7.1406	0.0815
Surgery	0.0814	0.0278 to 0.2379	< 0.0001
Adjuvant therapy	–	–	–
T	–	–	–
N	3.5458	1.4546 to 8.6434	0.0054
M	–	–	–
mDR genes	3.0126	1.0707 to 8.4764	**0.0367**
Surgery	0.0957	0.0306 to 0.2998	0.0001
Adjuvant therapy	0.3642	0.1100 to 1.2057	0.0980
T	–	–	–
N	0.2280	1.7129 to 10.4365	0.0180
M	–	–	–
Mutation load rate	1.0018	1.0005 to 1.0032	**0.0090**
Surgery	0.1417	0.0510 to 0.3933	0.0002
Adjuvant therapy	0.2807	0.0750 to 1.0498	0.0590
T	–	–	–
N	3.4240	1.2897 to 9.0905	0.0140
M	–	–	–

In bold, significant values for molecular markers are indicated

T, primary tumor; N, regional lymph node; M, distant metastasis

## Discussion

In this study, we describe a somatic mutational profile with a NGS targeted panel in a retrospective cohort of 55 patients with PDAC, consecutively collected in a hospital-based manner and ascertained for Sardinian origin.

In our series, a high prevalence of oncogenic mutations was observed for *KRAS* gene (62%). However, the frequency of activating mutations in *KRAS* was lower than expected since they are reported in literature to be present up to 80%-95% of PDACs [30, 31]; this lower prevalence may be probably due to the “genetic background” of the Sardinian population, showing strong founder effects for several genetic diseases. Indeed, as previously reported by our group for different neoplastic pathologies (colorectal and breast cancer, melanoma) [32–34]), the founder effect in Sardinia may mostly act at germline level but also underlay discrepant penetrance and distribution of mutations in candidate cancer genes at somatic level.

The spectrum of the *KRAS* activating mutations is instead highly consistent with data from literature [35], with G12D as the most prevalent variant (35%) and the G12V or G12R as the only mutations affecting codon 12 into the nucleotide-binding pocket of the *KRAS* kinase domain (Additional file 1: Table S1). It is noteworthy to underline that the most frequent G12D mutation is critical for the initiation and maintenance of PDAC and, at the same time, acts as a known repressor of tumor immunity [36]. In our series, the KRAS-G12C mutation was not detected—though this observation is not far from expected (the G12C variant is reported in approximately 1.5% of PDACs [37, 38]). Despite its very low prevalence, systematic search for this variant might open the way to the use of KRAS-G12C inhibitor (i.e. Sotorasib, which was already evaluated in monotherapy in metastatic KRAS-G12C mutant PDAC, with a limited clinical benefit due to the rapid development of resistance [31]) given in sequence or combination with other drugs. Overall, our findings confirmed the central pathogenic role of *KRAS* mutations, which are highly abundant in pancreatic cancer, inducing to speculate that such a mutated oncogene may participate in early molecular events toward the progression from normal pancreatic tissues to malignant PDAC. Moreover, the KRAS-G12D mutation occurs in nearly half of PDAC patients and its oncogenic signaling has been reported to contribute in maintaining the immune evasive status of the tumor microenvironment (TME) in such cancer type [39, 40], by also suppressing PD-L1 expression, thus impairing the activity of immune checkpoint inhibitors (ICIs) in PDAC and explaining the observed low response of PDAC to immunotherapy [41–43]. Recently, KRAS-G12D mutations have been reported to drive the fibroblast expansion toward the creation of an immunosuppressive stroma from early stages of pancreatic malignant transformation [44]. In lung cancer, the detrimental activity of the oncogenic KRAS-G12D variant into the responsiveness to ICIs has been largely demonstrated [45, 46]. As a confirmation of the negative impact of the KRAS-G12D mutation on the immune responsiveness, inhibition of such mutant protein with MRTX1133, a specific small molecule inhibitor of KRAS-G12D variant, has been reported to somehow revert the immune suppressive status of TME by increasing the intra-tumor infiltrate of CD8^+^ effector T cells and contributing to make PDAC more immunogenic and better reactive to immunotherapy [47–49].

Regarding the relationship between CNV and mutation occurrence in *KRAS* gene, our data excluded any role of the gene amplification on determining a high frequency of *KRAS* mutant alleles in primary PDACs at baseline (such a scenario could be however modified during treatment course). This is quite consistent with data presented by our group about the minority of melanoma patients with concurrent high frequency of *BRAF* mutations and *BRAF* gene amplification [50].

In addition to *KRAS*, our PDAC cases displayed an increased frequency of genetic alterations in hallmark genes such as *TP53* (62%), *MYC* (47%), *APC* (38%), *NF1* (33%), and *SMAD4* (33%). The *TP53* genetic alterations have been demonstrated to exert an impact on the homeostasis of the TME in PDAC tissues, acting the gene as one of the principal modulators of disease progression by shaping the tumor-stromal environment [51]. Recently, functional significance of the different *TP53* mutations have been related to outcome of PDAC patients, with the pathogenic variants determining a gain of function of the *TP53* gene being associated with worse overall survival [52].

Nevertheless, the activation of WNT/β-catenin signaling pathway—including the downstream WNT effectors *CTNNB1* and *APC*, which were found mutated in a large fraction (22/55; 40%) of PDAC cases in our series—is another critical molecular mechanism for pancreatic tumor initiation and progression as well as for facilitating immune evasion and contributing to immunotherapy resistance of such a tumor [53]. The genes of this pathway act as common oncogenic drivers even in other non-PDAC malignancies [54].

Among the single genes screened in our series, *SMAD4* was the only one whose mutations remained significantly related to worse survival in multivariate analysis. *SMAD4* is a tumor suppressor gene that has been demonstrated to regulate cell proliferation and differentiation as well as interfere with the immune responses in PDAC [55]. The *SMAD4* mutations have long been known for their negative impact on survival [56]; moreover, they were mainly associated with a poor prognosis in PDAC patients who do not harbor mutations in *KRAS* [57]

Altogether, such evidence on intracellular genetic signatures from our real-life study combined with above mentioned findings from literature markedly underline a consistently high refractoriness of PDACs to immune activation. Considering the content of the extracellular compartment, the TME of such a malignancy is similarly characterized by a preponderance of immunosuppressive elements—including regulatory T cells (Tregs) and myeloid-derived suppressor cells (MDSCs), strongly interfering with the recognition of cancer cells by the immune system—as well as a peculiar desmoplastic structure with a limited amount of tumor-infiltrating lymphocytes (TILs) [58–61]. Overall, this contributes to explain the historical disappointment for clinical benefits of treatment with ICIs in monotherapy among advanced PDACs, as compared to other cancers [62, 63]. Recent evidence suggests that immunotherapies may show promises when included in combined treatments with single- or multi-agent chemotherapy (alone or in chemoradiation protocols), vaccination, or other immunotherapeutic compounds targeting different mechanisms involved in T cell immune regulation [64, 65].

High level of microsatellite instability (MSI-H), which is caused by a deficient DNA mismatch repair system, has been instead recognized as a highly effective biomarker to select good responders to immunotherapy in multiple cancer types, since its occurrence favors higher levels of CD8 + tumor infiltrating lymphocytes in TME [66]. Unfortunately, only a very limited subset of PDAC patients is harboring tumors with MSI-H or MMR deficiency (< 2%) [67]. On this regard, the prevalence of MSI-H in our series (1/55; 1.8%) was highly consistent with frequencies reported in literature.

High tumor mutation burden (TMB) has been indicated as a putative biomarker for predicting the response to immunotherapy in many cancers through an increased production of neoantigens, which in turn make the tumors more immunogenic [22]. However, clear evidence about the role of high TMB in predicting both the response to immunotherapy and/or a favorable outcome in PDAC is still lacking [68] as well as the relationship between TMB and MSI in determining the impact on survival. Some authors identified a PDAC subgroup with low TMB and MSI-H associated with prolonged OS [69], whereas others reported a better outcome in a PDAC subset harboring a tumor with high TMB and microsatellite stability [70].

**Fig. 3:**
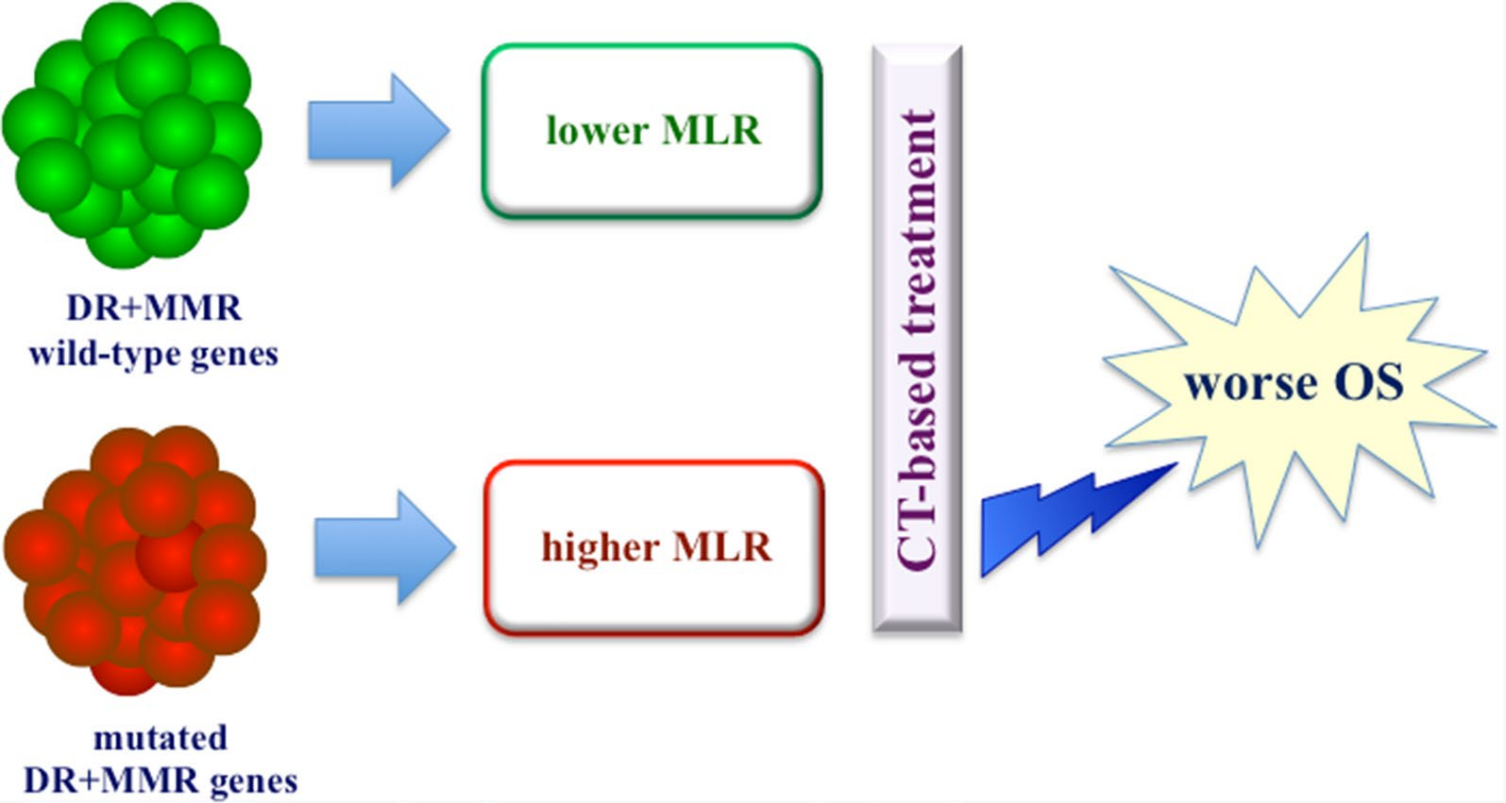
A model representing the main effects of the occurrence of combined mutations in repair genes in our PDAC series

In our series, mutated genes involved in DNA damage repair (DR) and high mutational load rate (MLR) were not significantly correlated with survival when taken singularly. In multivariate analysis models, which included the most relevant clinical confounders identified (disease stage, surgery, adjuvant treatments), such alterations were significantly associated with worse survival (HR 1.0018, 95% CI 1.0005 to 1.0032; p < 0.01). It was particularly interesting the fact that one fourth of our PDAC cases (14/55; 25.5%) carried tumors presenting a combination of mutations in repair genes (DR and MMR, including *POLE* and *POLD1* genes participating to base excision repair mechanisms) and the highest mutation load rates (MLR-H; see Fig. 2). This represents a further confirmation that impairments of the genes controlling the DNA damage repair and DNA replication markedly lead to the accumulation of mutations caused by replicative errors. One could speculate that the specific subset of PDAC patients carrying the combination of mutated repair genes and MLR-H, which is overall associated with a worse survival in our series treated with conventional chemotherapy protocols **(**Fig. 3**)**, might have a good chance to be sensitive to ICI-based immunotherapies. If true, the combination of these alterations may act as a predictive biomarker of response to immunotherapy in addition to their prognostic role in predicting survival.

Our data on prevalence of TMB in PDAC are not consistent with those reported in literature (less than 5% of cases with high-TMB) [23, 71], though it should be kept in mind that our MLR estimate cannot be considered as a canonical TMB value. On the other hand, higher rates of mutations within different cancer types carrying altered MMR and/or DR genes have been reported in literature; such data are thus highly consistent with our findings [71–73].

From the practical point of view, our study further demonstrated that NGS-based profiling of PDAC is nowadays needed to offer the opportunity to better dissect the genomic landscape of the disease, helping to identify and characterize the distinct molecular patients’ subgroups. More comprehensive genomic profiling assays may provide some advantages for the management of PDAC patients, being able to identify actionable targets and biomarkers of immune sensitivity or resistance. These efforts may pave the way for combination or sequence of tailored treatments. A recent retrospective study has indeed clearly indicated that PDAC patients who carry actionable molecular alterations can derive considerable benefit from receiving a matched targeted therapy [25].

We are aware that several limitations are present in this work, including its retrospective nature and the limitedness of our patients’ collection. The small sample size of our retrospective series is mostly due to the quality of the genomic DNA isolated from archive tumor specimens that underwent different tissue handling processes and non-codified pre-analytical procedures during a quite long past time period (from July 2014 to June 2020, aimed at having a longer follow-up and a better survival correlation: see Methods). In other words, factors affecting samples’ quality reduced the suitability for a highly performing NGS analysis. For the retrospective nature of the study, the absence of some types of additional clinical information (i.e. T-cell infiltration density, more detailed data about course and comorbidities of the patients), which might somehow influence the outcome, enforces us to plan a prospective study including a larger sample size of PDAC patients at various stages of the disease.

## Conclusion

All information coming from translational trials and real-life studies as ours may contribute to identify patterns of molecular alterations as innovative biomarkers useful to manage patients into the clinical practice. Although chemotherapy will surely remain a milestone of PDAC treatment, the future successful direction to treat such a disease cannot rely on a one-size-fits-all approach but on the identification of specific tumor-associated targets and molecular alterations as a turning point for the development of a more extensive armamentarium of therapeutic options and the subsequent assessment of treatment algorithms for optimizing the cure of PDAC patients. In this sense, our findings—obtained in a real-world hospital-based collected series—open the way to address to ICI-based immunotherapies the subset of PDAC patients carrying the combination of mutated repair genes and MLR-H, which instead was found to be associated with a poorer survival under conventional chemotherapy-based treatments.

### Supplementary Information

**Additional file 1: Table S1.** All somatic non-synonymous variants found in each PDAC sample. Variants were classified as pathogenic or likely pathogenic in accordance with the criteria reported in COSMIC and ClinVar databases (see text), also indicating the Oncomine gene variant classes. Coverage (total number of sequence reads) and frequency of the mutated alleles are also indicated.

**Additional file 2: Table S2.** Synthesis of pathogenic/likely pathogenic mutations detected in our series. Results of mutation analysis are reported for main candidate genes involved in PDAC pathogenesis: KRAS, uncommon NRAS variants (NRASuncom), BRAF nonV600 variants (BRAFnonV600), PIK3CA, CDKN2A, TP53, SMAD4, POLE, and POLD1. Occurrence of mutations in mismatch repair (MMR) and damage repair (DR) genes as well as results from the analysis of microsatellite instability (MSI) and the total amount of variants observed (mutation load rate: number of mutations per megabase) are also reported. In table columns, “0” and “1” values mean absence and presence of the genomic alterations, respectively.

## Data Availability

All data generated during the current study, including results from mutational analysis, are available from the corresponding author on reasonable request; most of them has already been included as supplementary Tables and Figures.

## Abbreviations

AJCC: American Joint Commission on Cancer
ECOG: Eastern Cooperative Oncology Group
CNV: Copy number variation
DR: Damage repair
FFPE: Formalin-fixed paraffin-embedded
MLR: Mutation load rates
MMR: Mismatch repair
MSI: Microsatellite instability
NGS: Next-generation sequencing
OS: Overall survival
PDCA: Pancreatic ductal adenocarcinoma
TMB: Tumor mutational burden
TME: Tumor microenvironment
TNM: Tumor-node-metastasis
VAF: Variant allele frequency
