# Supplementary material for: Combination of mutations in genes controlling DNA repair and high mutational load plays a prognostic role in pancreatic ductal adenocarcinoma (PDAC): a retrospective real-life study in Sardinian population
Source: J Transl Med. 2024 Jan 27;22:108. doi: 10.1186/s12967-024-04923-3 (PMC10821545; doi:10.1186/s12967-024-04923-3)
Supplement: Supplementary file 1 — Additional file 1: Table S1. All somatic non-synonymous variants found in each PDAC sample. Variants were classified as pathogenic or likely pathogenic in accordance with the criteria reported in COSMIC and ClinVar databases (see text), also indicating the Oncomine gene variant classes. Coverage (total number of sequence reads) and frequency of the mutated alleles are also indicated. [file 12967_2024_4923_MOESM1_ESM.pdf]

ID	Genes	Locus	Genotype	Type	Length	Oncomine Variant Class	Oncomine Gene Class	Variant ID	% Frequency	Mutated Alleles	Amino Acid Change	Coverage	Exon	Transcript	Coding	Variant Effect	PolyPhen	FATHMM	ClinVar	p-value
B19-04468	KRAS	chr12:25398284	CC/TC	SNV	1	Hotspot	Gain-of-Function	COSM521	11,4	43,0	p.Gly12Asp	379	2	NM_033360,3	c.35G>A	missense	0,517	0,98(COSM521)	Pathogenic	0,00001
B19-04468	MYC	chr8:128750803	C/G	SNV	1			COSM6209969	6,3	33,0	p.Leu114Val	524	2	NM_002467,5	c.340C>G	missense	0,855	0,96(COSM6209969)		0,00001
B19-04468	TP53	chr17:7578499	T/A	SNV	1			COSM43783	5,5	51,0	p.Gln144Leu	931	5	NM_000546,5	c.431A>T	missense	0,67	0,98(COSM43783)	Likely pathogenic	0,00001
B19-06245	KRAS	chr12:25398284	CC/TC	SNV	1	Hotspot	Gain-of-Function	COSM521	5,6	13,0	p.Gly12Asp	232	2	NM_033360,3	c.35G>A	missense	0,517	0,98(COSM521)	Pathogenic	0,01242
B19-09926	MYC	chr8:128750803	C/G	SNV	1			COSM6209969	5,9	90,0	p.Leu114Val	1523	2	NM_002467,5	c.340C>G	missense	0,855	0,96(COSM6209969)		0,00001
B19-09926	PTEN	chr10:89717767	G/A	SNV	1			COSM5351742	5,6	34,0	p.Met264Ile	603	7	NM_000314,6	c.792G>A	missense	0,007	0,99(COSM5351742)		0,00001
B20-00103	APC	chr5:112175769	GGAA/GAA	INDEL	1	Deleterious	Loss-of-Function	COSM19626	9,7	35,0	p.Glu1494LysfsTer13	360	16	NM_000038,5	c.4480delG	frameshiftDeletion		1,00COSM19626		0,00001
B20-00103	APC	chr5:112175558	C/T	SNV	1			COSM6196801	5,1	14,0	p.Leu1423Phe	273	16	NM_000038,5	c.4267C>T	missense	0,999	0,92(COSM6196801)		0,00471
B20-00103	ATM	chr11:108236204	G/A	SNV	1			COSM1351063	7,2	32,0	p.Arg3047Gln	445	63	NM_000051,3	c.9140G>A	missense	0,65	0,96(COSM1351063)	Uncertain significance	0,00001
B20-00103	CDH1	chr16:68849586	G/A	SNV	1			COSM1247778	5,2	42,0	p.Glu497Lys	805	10	NM_004360,4	c.1489G>A	missense	1	1,00(COSM1,00247778)		0,00001
B20-00103	CDKN2A	chr9:21971116	G/C	SNV	1			COSM6985959	10,1	36,0	p.Pro81Arg	358	2	NM_001195132,1	c.242C>G	missense	1	0,98(COSM6985959)	Uncertain significance	0,00001
B20-00103	KRAS	chr12:25398284	CC/TC	SNV	1	Hotspot	Gain-of-Function	COSM521	11,9	21,0	p.Gly12Asp	177	2	NM_033360,3	c.35G>A	missense	0,517	0,98(COSM521)	Pathogenic	0,00001
B20-00103	KRAS	chr12:25398267	C/T	SNV	1			COSM541	6,1	11,0	p.Ala18Thr	180	2	NM_033360,3	c.52G>A	missense	0,995	0,98(COSM541)		0,00268
B20-00103	MYC	chr8:128750803	C/G	SNV	1			COSM6209969	5,8	29,0	p.Leu114Val	503	2	NM_002467,5	c.340C>G	missense	0,855	0,96(COSM6209969)		0,00002
B20-00103	PIK3CA	chr3:178937004	C/T	SNV	1			COSM9630050	6,4	29,0	p.Pro562Leu	456	11	NM_006218,3	c.1685C>T	missense	1	0,97(COSM9630050)		0,00001
B20-00103	RB1	chr13:49039236	G/T	SNV	1			COSM348849	6,0	34,0	p.Ala772Ser	569	22	NM_000321,2	c.2314G>T	missense	0,686	0,98(COSM348849)		0,00001
B20-02060	PIK3CA	chr3:178936091	G/A	SNV	1	Hotspot	Gain-of-Function	COSM763	6,4	128,0	p.Glu545Lys	2000	10	NM_006218,3	c.1633G>A	missense	0,991	0,97(COSM763)	Pathogenic/Likely pathogenic	0,00001
B20-02240	KRAS	chr12:25398284	CC/AC	SNV	1	Hotspot	Gain-of-Function	COSM520	8,6	17,0	p.Gly12Val	197	2	NM_033360,3	c.35G>T	missense	0,999	0,98(COSM520)	Pathogenic	0,00005
B20-02240	MYC	chr8:128750803	C/G	SNV	1			COSM6209969	5,1	15,0	p.Leu114Val	292	2	NM_002467,5	c.340C>G	missense	0,855	0,96(COSM6209969)		0,00363
B20-02240	POLE	chr12:133220098	CCA/C	INDEL	2	Deleterious	Loss-of-Function	COSM1745059	6,7	21,0	p.Val1446GlyfsTer3	315	34	NM_006231,3	c.4337_4338delTG	frameshiftDeletion		1,00(COSM1745059)	Uncertain significance	0,00027
S14-00921	APC	chr5:112173848	G/A	SNV	1			COSM6475562	20,2	21,0	p.Glu853Lys	104	16	NM_000038,5	c.2557G>A	missense	0,094	0,99(COSM6475562)	Uncertain significance	0,00001
S14-00921	APC	chr5:112175174	G/A	SNV	1			COSM7134556	6,9	17,0	p.Glu1295Lys	247	16	NM_000038,5	c.3883G>A	missense	0,029	0,82(COSM7134556)		0,00008
S14-00921	APC	chr5:112174047	G/T	SNV	1			COSM4166215	8,5	11,0	p.Arg919Ile	129	16	NM_000038,5	c.2756G>T	missense	0,991	0,90(COSM4166215)		0,00017
S14-00921	ARID2	chr12:46215212	C/T	SNV	1			COSM3460961	5,9	28,0	p.Ser216Phe	478	6	NM_152641,3	c.647C>T	missense	0,998	0,96(COSM3460961)		0,00002
S14-00921	ARID2	chr12:46287447	G/A	SNV	1			COSM4663274	14,7	28,0	p.Arg1769Gln	191	20	NM_152641,3	c.5306G>A	missense	1	0,96(COSM4663274)		0,00001
S14-00921	ARID2	chr12:46245720	C/T	SNV	1	Deleterious	Loss-of-function	COSM4041992	8,8	23,0	p.Arg1272Ter	261	15	NM_152641,3	c.3814C>T	nonsense		0,94(COSM4041992)		0,00001
S14-00921	ARID2	chr12:46287315	C/T	SNV	1	Deleterious	Loss-of-function	COSM1628614	11,9	17,0	p.Arg1754Ter	143	19	NM_152641,3	c.5260C>T	nonsense		0,90(COSM1628614)		0,00001
S14-00921	ARID2	chr12:46205217	G/A	SNV	1			COSM6939540	9,9	10,0	p.Glu101Lys	101	4	NM_152641,3	c.301G>A	missense	1	1,00(COSM6939540)		0,00012
S14-00921	ATM	chr11:108106519	G/A	SNV	1			COSM9118690	12,2	34,0	p.Val152Met	278	5	NM_000051,3	c.454G>A	missense	1	0,99(COSM9118690)		0,00001
S14-00921	ATM	chr11:108196911	C/T	SNV	1			COSM1235407	6,4	27,0	p.Leu2312Phe	422	47	NM_000051,3	c.6934C>T	missense	1	0,99(COSM1235407)		0,00001
S14-00921	ATM	chr11:108186557	T/C	SNV	1			COSM7351415	8,4	24,0	p.Leu2005Pro	286	41	NM_000051,3	c.6014T>C	missense	1	0,98(COSM7351415)		0,00001
S14-00921	ATM	chr11:108141816	C/T	SNV	1			COSM1492404	9,8	19,0	p.Pro955Leu	193	19	NM_000051,3	c.2864C>T	missense	0,314	0,99(COSM1492404)		0,00001
S14-00921	ATM	chr11:108201089	C/T	SNV	1	Deleterious	Loss-of-function	COSM1351002	6,3	13,0	p.Arg2486Ter	206	50	NM_000051,3	c.7456C>T	nonsense		0,94(COSM1351002)	Pathogenic/Likely pathogenic	0,00098
S14-00921	BRAF	chr7:140453109	T/C	SNV	1			COSM21613	7,6	30,0	p.Gln609Arg	393	15	NM_004333,5	c.1826A>G	missense	0,017	0,98(COSM21613)		0,00001
S14-00921	BRAF	chr7:140481401	TCC/TTC	SNV	1	Hotspot	Gain-of-function	COSM461	6,5	20,0	p.Gly469Glu	306	11	NM_004333,5	c.1406G>A	missense	0,999	0,99(COSM461)	Pathogenic	0,00052
S14-00921	CDH1	chr16:68862194	G/A	SNV	1			COSM4824743	8,3	36,0	p.Gly761Glu	436	14	NM_004360,4	c.2282G>A	missense	1	0,99(COSM4824743)	Uncertain significance	0,00001
S14-00921	CTNNB1	chr3:41266073	C/T	SNV	1			COSM5748	5,5	22,0	p.His24Tyr	400	3	NM_001904,3	c.70C>T	missense	0,035	0,95(COSM5748)		0,00028
S14-00921	FANCD2	chr3:10081030	G/A	SNV	1			COSM5805919	17,0	17,0	p.Val187Met	100	8	NM_033084,4	c.559G>A	missense	0,973	0,91(COSM5805919)		0,00001
S14-00921	KIT	chr4:55599308	G/A	SNV	1			COSM7335268	10,0	23,0	p.Gly812Ser	229	17	NM_000222,2	c.2434G>A	missense	1	0,99(COSM7335268)		0,00001
S14-00921	KIT	chr4:55602667	C/T	SNV	1			COSM28588	8,4	16,0	p.Arg830Ter	191	18	NM_000222,2	c.2488C>T	nonsense		0,97(COSM28588)		0,00001
S14-00921	KRAS	chr12:25380285	G/A	SNV	1			COSM87288	15,0	41,0	p.Thr58Ile	274	3	NM_033360,3	c.173C>T	missense	1	0,99(COSM87288)	Pathogenic	0,00001
S14-00921	KRAS	chr12:25380283	C/A	SNV	1	Hotspot	Gain-of-function	COSM1235389	6,9	19,0	p.Ala59Ser	274	3	NM_033360,3	c.175G>T	missense	0,745	0,98(COSM1235389)	Likely pathogenic	0,00033
S14-00921	MLH1	chr3:37090086	C/T	SNV	1	Deleterious	Loss-of-function	COSM1692535	21,1	28,0	p.Arg659Ter	133	17	NM_000249,3	c.1975C>T	nonsense		0,97(COSM1692535)	Pathogenic	0,00001
S14-00921	MLH1	chr3:37056023	C/T	SNV	1			COSM9361837	5,5	19,0	p.Leu260Phe	347	9	NM_000249,3	c.778C>T	missense	0,971	0,99(COSM9361837)	Uncertain significance	0,00068
S14-00921	MSH2	chr2:47643501	C/T	SNV	1	Deleterious	Loss-of-function	COSM1215558	14,4	18,0	p.Gln337Ter	125	6	NM_000251,2	c.1009C>T	nonsense		0,99(COSM1215558)	Pathogenic	0,00001
S14-00921	MSH6	chr2:48026963	C/T	SNV	1			COSM6973078	28,6	32,0	p.Ser614Phe	112	4	NM_000179,2	c.1841C>T	missense	0,205	0,96(COSM6973078)		0,00001
S14-00921	NF1	chr17:29654820	G/A	SNV	1			COSM71825	6,5	29,0	p.Ala1858Thr	449	38	NM_001042492,2	c.5572G>A	missense	0,824	0,99(COSM71825)	Uncertain significance	0,00001
S14-00921	NF1	chr17:29576112	G/A	SNV	1			COSM6947513	12,6	24,0	p.Arg1362Gln	190	30	NM_001042492,2	c.4085G>A	missense	0,999	0,88(COSM6947513)	Uncertain significance	0,00001
S14-00921	NF1	chr17:29576111	C/T	SNV	1	Deleterious	Loss-of-function	COSM24443	9,0	17,0	p.Arg1362Ter	189	30	NM_001042492,2	c.4084C>T	nonsense		1,00(COSM24443)	Pathogenic	0,00003
S14-00921	NRAS	chr1:115256542	C/T	SNV	1			COSM6438073	11,1	13,0	p.Asp57Asn	117	3	NM_002524,4	c.169G>A	missense	0,999	0,99(COSM6438073)		0,00001
S14-00921	PALB2	chr16:23637618	G/A	SNV	1			COSM9310492	5,6	19,0	p.Ser896Phe	342	7	NM_024675,3	c.2687C>T	missense	1	0,98(COSM9310492)		0,00057
S14-00921	PIK3CA	chr3:178951971	G/A	SNV	1			COSM28544	13,8	35,0	p.Gly1009Glu	253	21	NM_006218,3	c.3026G>A	missense	1	0,99(COSM28544)		0,00001
S14-00921	PIK3CA	chr3:178921548	G/A	SNV	1	Hotspot	Gain-of-function	COSM253279	7,0	26,0	p.Val344Met	372	5	NM_006218,3	c.1030G>A	missense	1	0,99(COSM253279)	Pathogenic/Likely pathogenic	0,00004
S14-00921	PIK3CA	chr3:178919314	C/A	SNV	1			COSM7909985	10,6	11,0	p.Leu267Met	104	4	NM_006218,3	c.799C>A	missense	0,82	0,90(COSM7909985)		0,00004
S14-00921	PIK3CA	chr3:178952141	G/A	SNV	1			COSM6941467	6,8	11,0	p.Ala1066Thr	161	21	NM_006218,3	c.3196G>A	missense	0,141	0,95(COSM6941467)		0,00109
S14-00921	POLE	chr12:133249314	C/A	SNV	1			COSM430778	13,0	13,0	p.Asp529Tyr	100	15	NM_006231,3	c.1585G>T	missense	0,773	0,84(COSM430778)		0,00001
S14-00921	POLE	chr12:133212542	C/A	SNV	1			COSM385240	8,1	12,0	p.Trp1916Leu	149	42	NM_006231,3	c.5747G>T	missense	0,996	0,99(COSM385240)		0,00027
S14-00921	PTEN	chr10:89717652	C/T	SNV	1			COSM1702738	15,9	21,0	p.Ser226Phe	132	7	NM_000314,6	c.677C>T	missense	0,773	0,98(COSM1702738)		0,00001
S14-00921	PTEN	chr10:89717749	C/A	SNV	1			COSM3807906	9,5	20,0	p.Phe258Leu	210	7	NM_000314,6	c.774C>A	missense	0,896	0,97(COSM3807906)		0,00001
S14-00921	RB1	chr13:49027196	C/T	SNV	1			COSM5674798	5,3	33,0	p.Ser588Phe	620	18	NM_000321,2	c.1763C>T	missense	0,246	0,77(COSM5674798)	Uncertain significance	0,00003
S14-00921	RB1	chr13:48921966	G/A	SNV	1			COSM6366319	6,8	29,0	p.Cys169Tyr	427	5	NM_000321,2	c.506G>A	missense	0,493	0,93(COSM6366319)		0,00001
S14-00921	SMAD4	chr18:48584572	C/T	SNV	1	Deleterious	Loss-of-function	COSM4728228	10,1	22,0	p.Gln249Ter	217	6	NM_005359,5	c.745C>T	nonsense		0,89(COSM4728228)		0,00001
S14-00921	TP53	chr17:7579844	C/T	SNV	1	Deleterious	Loss-of-function	COSM4556687	11,7	16,0	p.Trp23Ter	137	2	NM_000546,5	c.69G>A	nonsense		0,91(COSM4556687)		0,00001
S14-00921	VHL	chr3:10191501	T/A	SNV	1			COSM17785	6,0	10,0	p.Val165Asp	168	3	NM_000551,3	c.494T>A	missense	1	0,91(COSM17785)		0,00445
S14-04316	CDKN2A	chr9:21971169	TCCGC/C	INDEL	11	Deleterious	Loss-of-function	COSM12516	12,3	241,0	p.Glu61AlafsTer55	1956	2	NM_001195132,1	c.178_188delGCGGAGCTGCT	frameshiftDeletion		1,00(COSM12516)		0,00001
S14-04316	KRAS	chr12:25398284	CC/CG	SNV	1	Hotspot	Gain-of-function	COSM518	7,4	91,0	p.Gly12Arg	1233	2	NM_033360,3	c.34G>C	missense	0,741	0,98 (COSM518)	Pathogenic	0,00001
S14-04316	MYC	chr8:128750803	C/G	SNV	1			COSM6209969	8,6	86,0	p.Leu114Val	999	2	NM_002467,5	c.340C>G	missense	0,855	0,96 (COSM6209969)		0,00001
S14-04316	SMAD4	chr18:48593406	G/A	SNV	1	Hotspot	Loss-of-function	COSM1150895	10,1	201,0	p.Gly386Asp	2000	10	NM_005359,5	c.1157G>A	missense	1	0,99 (COSM1150895)	Pathogenic/Likely pathogenic	0,00001
S14-06208	ALK	chr2:29445408	G/T	SNV	1			COSM7848119	7,2	27,0	p.Pro1142Gln	377	21	NM_004304,4	c.3425C>A	missense	0,996	0,98(COSM7848119)		0,00001
S14-06208	APC	chr5:112175521	TGG/TGA	SNV	1			COSM6602036	18,4	75,0	p.Gly1412Glu	408	16	NM_000038,5	c.4235G>A	missense	0,994	0,85(COSM19592)		0,00001
S14-06208	APC	chr5:112154942	C/T	SNV	1	Deleterious	Loss-of-function	COSM19209	6,4	63,0	p.Arg405Ter	980	10	NM_000038,5	c.1213C>T	nonsense		0,96(COSM19209)	Pathogenic	0,00001
S14-06208	ARID2	chr12:46242663	G/A	SNV	1			COSM4663267	7,1	106,0	p.Arg542Gln	1493	13	NM_152641,3	c.1625G>A	missense	0,999	0,99(COSM4663267)		0,00001
S14-06208	ARID2	chr12:46230406	G/A	SNV	1			COSM69715	7,5	82,0	p.Arg247His	1099	7	NM_152641,3	c.740G>A	missense	1	0,99(COSM69715)		0,00001
S14-06208	ARID2	chr12:46244724	G/A	SNV	1			COSM7339283	7,0	53,0	p.Ala940Thr	761	15	NM_152641,3	c.2818G>A	missense	0,996	0,91(COSM7339283)		0,00001
S14-06208	ARID2	chr12:46231437	C/T	SNV	1			COSM1361683	16,8	52,0	p.Thr426Met	309	10	NM_152641,3	c.1277C>T	missense	0,999	1,00(COSM1361683)		0,00001
S14-06208	ARID2	chr12:46244796	G/T	SNV	1	Deleterious	Loss-of-function	COSM3460972	10,5	33,0	p.Gly964Ter	314	15	NM_152641,3	c.2890G>T	nonsense		0,98(COSM3460972)		0,00001
S14-06208	ATM	chr11:108172385	C/T	SNV	1	Deleterious	Loss-of-function	COSM172204	10,8	100,0	p.Arg1730Ter	930	35	NM_000051,3	c.5188C>T	nonsense		0,85(COSM172204)	Pathogenic/Likely pathogenic	0,00001
S14-06208	ATM	chr11:108170548	G/A	SNV	1			COSM7167035	8,1	88,0	p.Asp1705Asn	1092	34	NM_000051,3	c.5113G>A	missense	0,034	0,94(COSM7167035)		0,00001

ID	Genes	Locus	Genotype	Type	Length	Oncomine Variant Class	Oncomine Gene Class	Variant ID	% Frequency	Mutated Alleles	Amino Acid Change	Coverage	Exon	Transcript	Coding	Variant Effect	PolyPhen	FATHMM	ClinVar	p-value
S14-06208	ATM	chr11:108165729	C/T	SNV	1	Deleterious	Loss-of-function	COSM1350874	11,7	81,0	p.Arg1618Ter	693	32	NM_000051,3	c.4852C>T	nonsense		0,85(COSM1350874)	Pathogenic	0,00001
S14-06208	ATM	chr11:108178641	C/T	SNV	1	Deleterious	Loss-of-function	COSM6904314	9,6	62,0	p.Arg1898Ter	646	38	NM_000051,3	c.5692C>T	nonsense		0,74(COSM6904314)	Pathogenic	0,00001
S14-06208	ATM	chr11:108196825	C/A	SNV	1	Deleterious	Loss-of-function	COSM8854315	5,2	50,0	p.Ser2283Ter	965	47	NM_000051,3	c.6848C>A	nonsense		0,86(COSM8854315)		0,00001
S14-06208	ATM	chr11:108196197	G/T	SNV	1	Deleterious	Loss-of-function	COSM6946276	8,6	45,0	p.Glu2245Ter	522	46	NM_000051,3	c.6733G>T	nonsense		0,90(COSM6946276)		0,00001
S14-06208	ATM	chr11:108155074	G/T	SNV	1			COSM922719	5,8	42,0	p.Lys1289Asn	727	26	NM_000051,3	c.3867G>T	missense	0,995	0,98(COSM922719)		0,00001
S14-06208	ATM	chr11:108196789	C/A	SNV	1			COSM6519562	5,6	38,0	p.Pro2271His	681	47	NM_000051,3	c.6812C>A	missense	1	1,00(COSM6519562)		0,00001
S14-06208	BAP1	chr3:52442570	G/A	SNV	1			COSM5490570	11,7	181,0	p.Arg59Trp	1547	4	NM_004656,3	c.175C>T	missense	1	0,98(COSM5490570)		0,00001
S14-06208	BAP1	chr3:52441283	G/A	SNV	1			COSM6609923	7,7	60,0	p.Arg163Trp	782	7	NM_004656,3	c.487C>T	missense	1	0,98(COSM6609923)		0,00001
S14-06208	BAP1	chr3:52436392	C/T	SNV	1			COSM9231255	8,8	25,0	p.Arg701His	285	17	NM_004656,3	c.2102G>A	missense	0,995	0,96(COSM9231255)		0,00001
S14-06208	CTNNB1	chr3:41266888	G/A	SNV	1			COSM327070	5,1	62,0	p.Ala187Thr	1207	5	NM_001904,3	c.559G>A	missense	0,999	0,98(COSM327070)		0,00001
S14-06208	CTNNB1	chr3:41275179	C/T	SNV	1			COSM317204	11,3	43,0	p.Arg449Cys	380	9	NM_001904,3	c.1345C>T	missense	0,822	0,99(COSM317204)		0,00001
S14-06208	CTNNB1	chr3:41266097	G/T	SNV	1	Hotspot	Gain-of-function	COSM5661	6,8	22,0	p.Asp32Tyr	322	3	NM_001904,3	c.94G>T	missense	1	0,98(COSM5661)	Pathogenic/Likely pathogenic, ot	0,00017
S14-06208	EGFR	chr7:55266472	G/A	SNV	1			COSM3639753	10,5	52,0	p.Glu922Lys	494	23	NM_005228,4	c.2764G>A	missense	0,927	0,99(COSM3639753)		0,00001
S14-06208	EGFR	chr7:55268059	G/A	SNV	1			COSM5574310	10,6	52,0	p.Glu967Lys	493	24	NM_005228,4	c.2899G>A	missense	0,999	0,99(COSM5574310)		0,00001
S14-06208	GNAQ	chr9:80412542	C/T	SNV	1			COSM1253350	7,2	15,0	p.Val167Ile	209	4	NM_002072,4	c.499G>A	missense	0	0,80(COSM1253350)		0,00012
S14-06208	KIT	chr4:55599308	G/A	SNV	1			COSM7335268	8,0	89,9	p.Gly812Ser	1130	17	NM_000222,2	c.2434G>A	missense	1	0,99(COSM7335268)		0,00001
S14-06208	KIT	chr4:55593644	CAA/AAA	SNV	1			COSM133754	7,3	47,0	p.Pro573Gln	640	11	NM_000222,2	c.1718C>A	missense	0,991	0,90(COSM79375)		0,00001
S14-06208	KIT	chr4:55597518	G/A	SNV	1			COSM6909331	12,9	41,0	p.Met722Ile	318	15	NM_000222,2	c.2166G>A	missense	0,363	0,99(COSM6909331)		0,00001
S14-06208	KRAS	chr12:25398300	C/T	SNV	1			COSM30620	6,7	20,0	p.Val7Met	299	2	NM_033360,3	c.19G>A	missense	1	0,98(COSM30620)		0,00004
S14-06208	KRAS	chr12:25398219	G/A	SNV	1			COSM27637	5,1	15,0	p.Pro34Ser	295	2	NM_033360,3	c.100C>T	missense	1	0,99(COSM27637)		0,00401
S14-06208	NF1	chr17:29653016	G/A	SNV	1			COSM4706551	7,6	94,0	p.Asp1672Asn	1238	37	NM_001042492,2	c.5014G>A	missense	0,002	0,99(COSM4706551)	Uncertain significance	0,00001
S14-06208	NF1	chr17:29585404	G/A	SNV	1			COSM7463365	6,2	83,1	p.Gly1406Arg	1331	32	NM_001042492,2	c.4216G>A	missense	1	0,99(COSM7463365)		0,00001
S14-06208	NF1	chr17:29654857	G/A	SNV	1			COSM977479	6,2	78,1	p.Arg1870Gln	1261	38	NM_001042492,2	c.5609G>A	missense	0,998	0,90(COSM977479)	Pathogenic	0,00001
S14-06208	NF1	chr17:29587481	C/T	SNV	1			COSM977443	8,7	74,0	p.Arg1509Cys	855	34	NM_001042492,2	c.4525C>T	missense	1	0,95(COSM977443)	Uncertain significance	0,00001
S14-06208	NF1	chr17:29687626	C/T	SNV	1			COSM4990967	24,9	61,0	p.Pro2761Leu	245	57	NM_001042492,2	c.8282C>T	missense	0,998	0,98(COSM4990967)		0,00001
S14-06208	NF1	chr17:29559810	G/A	SNV	1			COSM1610050	6,4	34,0	p.Arg1136Gln	528	26	NM_001042492,2	c.3407G>A	missense	0,094	0,99(COSM1610050)		0,00001
S14-06208	PALB2	chr16:23646627	G/A	SNV	1	Deleterious	Loss-of-function	COSM6912572	10,2	76,0	p.Arg414Ter	748	4	NM_024675,3	c.1240C>T	nonsense		0,97(COSM6912572)	Pathogenic	0,00001
S14-06208	PIK3CA	chr3:178952097	G/A	SNV	1			COSM308549	19,5	101,0	p.Trp1051Ter	518	21	NM_006218,3	c.3152G>A	nonsense		0,97(COSM308549)		0,00001
S14-06208	PIK3CA	chr3:178952078	G/A	SNV	1	Hotspot	Gain-of-function	COSM25086	13,1	68,0	p.Asp1045Asn	518	21	NM_006218,3	c.3133G>A	missense	0,418	0,97(COSM25086)		0,00001
S14-06208	PIK3CA	chr3:178942501	C/T	SNV	1			COSM6191611	8,9	67,0	p.Arg770Ter	750	16	NM_006218,3	c.2308C>T	nonsense		0,94(COSM6191611)		0,00001
S14-06208	PIK3CA	chr3:178952100	C/A	SNV	1			COSM17447	6,8	35,0	p.Thr1052Lys	516	21	NM_006218,3	c.3155C>A	missense	0,305	0,96(COSM17447)		0,00001
S14-06208	POLD1	chr19:50909557	G/A	SNV	1			COSM6751939	15,6	66,0	p.Arg454His	424	11	NM_001256849,1	c.1361G>A	missense	1	0,99(COSM6751939)	Uncertain significance	0,00001
S14-06208	POLD1	chr19:50906355	C/T	SNV	1			COSM4456631	11,6	33,0	p.Ser339Leu	285	9	NM_001256849,1	c.1016C>T	missense	0,41	0,91(COSM4456631)		0,00001
S14-06208	POLE	chr12:133219425	C/T	SNV	1			COSM6909663	15,7	69,0	p.Arg1570Gln	439	36	NM_006231,3	c.4709G>A	missense	0,977	1,00(COSM6909663)		0,00001
S14-06208	POLE	chr12:133220551	G/T	SNV	1			COSM4040365	10,0	48,0	p.Leu1388Ile	480	33	NM_006231,3	c.4162C>A	missense	0,999	0,99(COSM4040365)		0,00001
S14-06208	POLE	chr12:133249803	C/T	SNV	1			COSM4040381	6,5	41,0	p.Val474Ile	631	14	NM_006231,3	c.1420G>A	missense	0,987	0,99(COSM4040381)	Uncertain significance	0,00001
S14-06208	PTEN	chr10:89717672	C/T	SNV	1	Deleterious	Loss-of-function	COSM5154	11,3	70,0	p.Arg233Ter	619	7	NM_000314,6	c.697C>T	nonsense		0,82(COSM5154)	Pathogenic	0,00001
S14-06208	RAD51C	chr17:56787223	C/T	SNV	1	Deleterious	Loss-of-function	COSM212455	8,5	35,0	p.Arg237Ter	411	5	NM_058216,2	c.709C>T	nonsense		0,78(COSM212455)	Pathogenic	0,00001
S14-06208	RB1	chr13:49054142	C/T	SNV	1	Deleterious	Loss-of-function	COSM7685017	18,3	97,0	p.Arg908Ter	531	27	NM_000321,2	c.2722C>T	nonsense		0,89(COSM7685017)		0,00001
S14-06208	SMAD4	chr18:48593519	G/A	SNV	1			COSM6917021	13,9	84,0	p.Asp424Asn	604	10	NM_005359,5	c.1270G>A	missense	0,575	1,00(COSM6917021)		0,00001
S14-06208	SMAD4	chr18:48593543	A/G	SNV	1			COSM14192	5,7	22,0	p.Ser432Gly	383	10	NM_005359,5	c.1294A>G	missense	0,217	0,97(COSM14192)		0,00016
S14-06208	TP53	chr17:7579361	A/G	SNV	1			COSM45169	7,5	78,0	p.Phe109Ser	1041	4	NM_000546,5	c.326T>C	missense	1	0,99(COSM45169)	Uncertain significance	0,00001
S14-07541	KRAS	chr12:25380275	TT/GT	SNV	1	Hotspot	Gain-of-function	COSM554	7,9	156,9	p.Gln61His	1989	3	NM_033360,3	c.183A>C	missense	0,09	0,93(COSM554)	Pathogenic/Likely pathogenic	0,00001
S14-07541	MYC	chr8:128750803	C/G	SNV	1			COSM6209969	6,5	70,0	p.Leu114Val	1076	2	NM_002467,5	c.340C>G	missense	0,855	0,96(COSM6209969)		0,00001
S14-07541	TP53	chr17:7577022	G/A	SNV	1	Deleterious	Loss-of-function	COSM10663	11,4	201,0	p.Arg306Ter	1771	8	NM_000546,5	c.916C>T	nonsense		0,95(COSM10663)	Pathogenic	0,00001
S14-08491	MYC	chr8:128750803	C/G	SNV	1			COSM6209969	8,0	144,1	p.Leu114Val	1810	2	NM_002467,5	c.340C>G	missense	0,855	0,96(COSM6209969)		0,00001
S14-08566	APC	chr5:112175891	G/A	SNV	1			COSM18840	5,6	54,0	p.Gly1534Arg	960	16	NM_000038,5	c.4600G>A	missense	0,7	0,90(COSM18840)		0,00001
S14-08566	APC	chr5:112173704	C/T	SNV	1	Deleterious	Loss-of-function	COSM19058	12,2	36,0	p.Arg805Ter	295	16	NM_000038,5	c.2413C>T	nonsense		0,83(COSM19058)	Pathogenic	0,00001
S14-08566	APC	chr5:112151204	C/T	SNV	1	Deleterious	Loss-of-function	COSM19679	11,0	31,0	p.Arg283Ter	281	9	NM_000038,5	c.847C>T	nonsense		0,98(COSM19679)	Pathogenic	0,00001
S14-08566	APC	chr5:112177049	C/T	SNV	1	Deleterious	Loss-of-function	COSM1059619	6,7	25,0	p.Arg1920Ter	376	16	NM_000038,5	c.5758C>T	nonsense		0,87(COSM1059619)		0,00001
S14-08566	ARID2	chr12:46211569	G/A	SNV	1			COSM4041981	6,7	61,0	p.Val179Ile	906	5	NM_152641,3	c.535G>A	missense	0,999	0,99(COSM4041981)		0,00001
S14-08566	ARID2	chr12:46246629	C/T	SNV	1	Deleterious	Loss-of-function	COSM5607878	6,4	30,0	p.Gln1575Ter	468	15	NM_152641,3	c.4723C>T	nonsense		0,99(COSM5607878)		0,00001
S14-08566	ARID2	chr12:46245210	G/T	SNV	1			COSM6981296	6,4	26,0	p.Ala1102Ser	404	15	NM_152641,3	c.3304G>T	missense	0,001	0,89(COSM6981296)		0,00001
S14-08566	ATM	chr11:108175528	C/T	SNV	1	Deleterious	Loss-of-function	COSM1350896	5,6	33,0	p.Arg1875Ter	591	37	NM_000051,3	c.5623C>T	nonsense		0,86(COSM1350896)	Pathogenic	0,00001
S14-08566	BRAF	chr7:140453149	C/T	SNV	1	Hotspot	Gain-of-function	COSM7807516	8,7	69,0	p.Gly596Ser	798	15	NM_004333,5	c.1786G>A	missense	1	0,98(COSM7807516)	Likely pathogenic	0,00001
S14-08566	FGFR2	chr10:123298200	C/T	SNV	1			COSM6956507	12,2	33,0	p.Met218Ile	270	6	NM_000141,4	c.654G>A	missense	0,99	0,99(COSM6956507)		0,00001
S14-08566	GNAQ	chr9:80430566	G/A	SNV	1			COSM3908208	7,4	89,0	p.Arg148Ter	1198	3	NM_002072,4	c.442C>T	nonsense		0,90(COSM3908208)		0,00001
S14-08566	KIT	chr4:55595622	G/T	SNV	1			COSM6980630	6,3	21,0	p.Lys704Asn	333	14	NM_000222,2	c.2112G>T	missense	0,999	0,95(COSM6980630)		0,00006
S14-08566	MLH1	chr3:37038192	G/A	SNV	1			COSM1422567	5,9	115,1	p.Gly67Arg	1947	2	NM_000249,3	c.199G>A	missense	1	0,98(COSM1422567)	Pathogenic	0,00001
S14-08566	MSH2	chr2:47705472	G/A	SNV	1			COSM3581877	6,9	23,0	p.Asp758Asn	335	14	NM_000251,2	c.2272G>A	missense	1	0,99(COSM3581877)	Uncertain significance	0,00001
S14-08566	MSH2	chr2:47702409	G/T	SNV	1			COSM6269155	5,4	18,0	p.Gly669Cys	332	12	NM_000251,2	c.2005G>T	missense	1	1,00(COSM6269155)	Conflicting interpretations of pat	0,00098
S14-08566	MSH6	chr2:48018188	G/A	SNV	1			COSM4094430	11,7	29,0	p.Arg128His	248	2	NM_000179,2	c.383G>A	missense	0,606	0,94(COSM4094430)	Uncertain significance	0,00001
S14-08566	MSH6	chr2:48030612	C/T	SNV	1			COSM6715816	5,2	24,0	p.Arg1076Cys	464	5	NM_000179,2	c.3226C>T	missense	0,991	0,92(COSM6715816)	Likely pathogenic	0,00041
S14-08566	MSH6	chr2:48026896	G/C	SNV	1			COSM6978171	6,1	22,0	p.Val592Leu	360	4	NM_000179,2	c.1774G>C	missense	0,277	0,99(COSM6978171)		0,00006
S14-08566	MYC	chr8:128750687	C/A	SNV	1			COSM3316887	17,5	35,0	p.Pro75His	200	2	NM_002467,5	c.224C>A	missense	1	0,99(COSM3316887)		0,00001
S14-08566	MYC	chr8:128750695	C/A	SNV	1			COSM6206287	8,0	16,0	p.Pro78Thr	200	2	NM_002467,5	c.232C>A	missense	1	0,99(COSM6206287)		0,00002
S14-08566	NF1	chr17:29585374	C/T	SNV	1			COSM6952368	7,4	53,0	p.Arg1396Cys	713	32	NM_001042492,2	c.4186C>T	missense	1	0,94(COSM6952368)	Uncertain significance	0,00001
S14-08566	NF1	chr17:29556323	G/A	SNV	1			COSM6264621	9,4	38,0	p.Arg897Gln	406	21	NM_001042492,2	c.2690G>A	missense	0,288	0,99(COSM6264621)	Uncertain significance	0,00001
S14-08566	PIK3CA	chr3:178947088	G/A	SNV	1			COSM9494939	6,5	66,0	p.Gly842Ser	1020	18	NM_006218,3	c.2524G>A	missense	1	0,99(COSM9494939)		0,00001
S14-08566	PIK3CA	chr3:178952115	G/A	SNV	1			COSM6475611	14,7	49,0	p.Trp1057Ter	334	21	NM_006218,3	c.3170G>A	nonsense		0,97(COSM6475611)		0,00001
S14-08566	PIK3CA	chr3:178936068	G/A	SNV	1			COSM1732635	7,0	48,0	p.Arg537Gln	687	10	NM_006218,3	c.1610G>A	missense	0,564	0,97(COSM1732635)		0,00001
S14-08566	PIK3CA	chr3:178952102	A/G	SNV	1			COSM3724544	5,4	18,0	p.Thr1053Ala	334	21	NM_006218,3	c.3157A>G	missense	0,993	0,96(COSM3724544)		0,00105
S14-08566	PMS2	chr7:6029581	C/T	SNV	1			COSM1091091	6,9	46,0	p.Val332Ile	663	10	NM_000535,6	c.994G>A	missense	0,387	0,91(COSM1091091)	Uncertain significance	0,00001
S14-08566	PMS2	chr7:6038848	C/T	SNV	1			COSM6971277	12,4	44,0	p.Arg199His	355	6	NM_000535,6	c.596G>A	missense	1	0,99(COSM6971277)	Uncertain significance	0,00001
S14-08566	POLD1	chr19:50909698	C/T	SNV	1			COSM3743064	28,9	59,0	p.Thr473Met	204	12	NM_001256849,1	c.1418C>T	missense	1	0,86(COSM3743064)		0,00001
S14-08566	POLD1	chr19:50906355	C/T	SNV	1			COSM4456631	17,2	37,0	p.Ser339Leu	215	9	NM_001256849,1	c.1016C>T	missense	0,41	0,91(COSM4456631)		0,00001
S14-08566	POLD1	chr19:50912904	C/T	SNV	1			COSM6751917	16,5	26,0	p.Pro712Leu	158	17	NM_001256849,1	c.2135C>T	missense	1	0,93(COSM6751917)		0,00001
S14-08566	POLD1	chr19:50906795	G/A	SNV	1			COSM8615946	7,4	17,0	p.Gly395Ser	231	10	NM_001256849,1	c.1183G>A	missense	1	0,91(COSM8615946)	Uncertain significance	0,00004

ID	Genes	Locus	Genotype	Type	Length	Oncomine Variant Class	Oncomine Gene Class	Variant ID	% Frequency	Mutated Alleles	Amino Acid Change	Coverage	Exon	Transcript	Coding	Variant Effect	PolyPhen	FATHMM	ClinVar	p-value
S14-08566	POLD1	chr19:50902651	G/C	SNV	1			COSM6933956	5,3	15,0	p.Asp76His	281	3	NM_001256849,1	c.226G>C	missense	0,906	0,93(COSM6933956)		0,00254
S14-08566	POLE	chr12:133202801	G/A	SNV	1	Deleterious	Loss-of-function	COSM4503587	5,3	35,0	p.Arg2145Ter	659	46	NM_006231,3	c.6433C>T	nonsense		0,92(COSM4503587)		0,00002
S14-08566	POLE	chr12:133220556	C/T	SNV	1			COSM8861111	5,1	23,0	p.Arg1386Gln	453	33	NM_006231,3	c.4157G>A	missense	0,992	0,99(COSM8861111)	Uncertain significance	0,00065
S14-08566	POLE	chr12:133254219	C/T	SNV	1			COSM5704365	10,8	11,0	p.Arg222His	102	7	NM_006231,3	c.665G>A	missense	1	0,99(COSM5704365)	Uncertain significance	0,00003
S14-08566	PTEN	chr10:89711918	G/T	SNV	1			COSM5249	5,3	31,0	p.Ser179Ile	587	6	NM_000314,6	c.536G>T	missense	0,612	0,94(COSM5249)		0,00006
S14-08566	PTEN	chr10:89711888	C/A	SNV	1			COSM5252	5,2	23,0	p.Pro169His	439	6	NM_000314,6	c.506C>A	missense	1	0,96(COSM5252)		0,00043
S14-08566	SMAD4	chr18:48593465	G/A	SNV	1			COSM14103	10,1	39,0	p.Ala406Thr	386	10	NM_005359,5	c.1216G>A	missense	1	0,99(COSM14103)	Uncertain significance	0,00001
S14-08566	TP53	chr17:7578211	C/A	SNV	1	Hotspot	Loss-of-function	COSM43650	7,4	51,0	p.Arg213Leu	687	6	NM_000546,5	c.638G>T	missense	1	0,99(COSM43650)	Likely pathogenic	0,00001
S14-08566	TP53	chr17:7577539	G/A	SNV	1	Hotspot	Loss-of-function	COSM10656	9,5	43,0	p.Arg248Trp	454	7	NM_000546,5	c.742C>T	missense	1	0,94(COSM10656)	Pathogenic	0,00001
S14-09082	KRAS	chr12:25398284	CC/CG	SNV	1	Hotspot	Gain-of-function	COSM518	10,7	111,0	p.Gly12Arg	1036	2	NM_033360,3	c.34G>C	missense	0,741	0,98 (COSM518)	Pathogenic	0,00001
S14-09082	MYC	chr8:128750803	C/G	SNV	1			COSM6209969	5,9	105,0	p.Leu114Val	1777	2	NM_002467,5	c.340C>G	missense	0,855	0,96 (COSM6209969)		0,00001
S15-01661	APC	chr5:112164587	G/A	SNV	1			COSM6602007	8,8	20,0	p.Arg554Gln	228	14	NM_000038,5	c.1661G>A	missense	0,427	0,99(COSM6602007)		0,00001
S15-01661	APC	chr5:112162834	C/T	SNV	1	Deleterious	Loss-of-function	COSM201292	5,6	14,0	p.Gln480Ter	252	12	NM_000038,5	c.1438C>T	nonsense		0,99(COSM201292)		0,00229
S15-01661	ARID2	chr12:46230405	C/A	SNV	1			COSM939495	15,3	20,0	p.Arg247Ser	131	7	NM_152641,3	c.739C>A	missense	0,997	0,90(COSM939495)		0,00001
S15-01661	ATM	chr11:108196917	C/T	SNV	1	Deleterious	Loss-of-function	COSM4433762	7,8	19,0	p.Gln2314Ter	243	47	NM_000051,3	c.6940C>T	nonsense		0,97(COSM4433762)		0,00001
S15-01661	ATM	chr11:108173586	G/T	SNV	1	Deleterious	Loss-of-function	COSM6947860	6,7	13,0	p.Glu1776Ter	194	36	NM_000051,3	c.5326G>T	nonsense		0,93(COSM6947860)	Pathogenic	0,00059
S15-01661	ATM	chr11:108201105	G/A	SNV	1	Deleterious	Loss-of-function	COSM6519564	8,5	11,0	p.Trp2491Ter	129	50	NM_000051,3	c.7472G>A	nonsense		0,99(COSM6519564)		0,00017
S15-01661	CDH1	chr16:68847282	G/A	SNV	1			COSM19750	20,7	24,0	p.Asp402Asn	116	9	NM_004360,4	c.1204G>A	missense	1	0,99(COSM19750)		0,00001
S15-01661	CTNNB1	chr3:41266046	G/A	SNV	1			COSM49161	7,0	21,0	p.Glu15Lys	301	3	NM_001904,3	c.43G>A	missense	0,113	0,98(COSM49161)		0,00001
S15-01661	NF1	chr17:29562668	C/T	SNV	1			COSM436322	19,1	21,0	p.Arg1250Trp	110	28	NM_001042492,2	c.3748C>T	missense	1	0,96(COSM436322)	Uncertain significance	0,00001
S15-01661	NRAS	chr1:115251226	C/T	SNV	1			COSM6729412	7,0	23,0	p.Arg167Gln	329	5	NM_002524,4	c.500G>A	missense	0,012	0,91(COSM6729412)		0,00001
S15-01661	PTEN	chr10:89717741	G/T	SNV	1	Deleterious	Loss-of-function	COSM5326	5,8	13,0	p.Glu256Ter	224	7	NM_000314,6	c.766G>T	nonsense		0,99(COSM5326)	Pathogenic	0,00213
S15-01661	RB1	chr13:48881438	G/A	SNV	1			COSM6946312	13,4	24,0	p.Glu54Lys	179	2	NM_000321,2	c.160G>A	missense	0,181	0,72(COSM6946312)		0,00001
S15-01661	RB1	chr13:48937061	C/A	SNV	1			COSM6763478	7,7	16,0	p.Leu277Ile	207	8	NM_000321,2	c.829C>A	missense	0,993	0,94(COSM6763478)		0,00003
S15-01661	SMAD4	chr18:48603021	G/A	SNV	1			COSM6784682	6,5	21,0	p.Arg441His	321	11	NM_005359,5	c.1322G>A	missense	0,054	0,99(COSM6784682)		0,00003
S15-01661	TP53	chr17:7574003	G/A	SNV	1	Deleterious	Loss-of-function	COSM11073	21,3	77,0	p.Arg342Ter	362	10	NM_000546,5	c.1024C>T	nonsense		0,73(COSM11073)	Pathogenic	0,00001
S15-02745	APC	chr5:112177508	G/A	SNV	1			COSM8312420	11,4	28,0	p.Gly2073Ser	246	16	NM_000038,5	c.6217G>A	missense	0	0,98(COSM8312420)		0,00001
S15-02745	APC	chr5:112175996	G/A	SNV	1			COSM5990842	10,7	21,0	p.Asp1569Asn	197	16	NM_000038,5	c.4705G>A	missense	0,978	0,98(COSM5990842)	Uncertain significance	0,00001
S15-02745	ARID2	chr12:46242711	G/A	SNV	1			COSM2067816	13,7	34,0	p.Arg558His	249	13	NM_152641,3	c.1673G>A	missense	1	0,99(COSM2067816)		0,00001
S15-02745	ARID2	chr12:46287446	C/T	SNV	1	Deleterious	Loss-of-function	COSM1628616	16,8	28,0	p.Arg1769Ter	167	20	NM_152641,3	c.5305C>T	nonsense		0,87(COSM1628616)	Pathogenic	0,00001
S15-02745	ATM	chr11:108106466	G/A	SNV	1			COSM7343605	6,6	18,0	p.Gly134Asp	275	5	NM_000051,3	c.401G>A	missense	0,119	0,91(COSM7343605)	Uncertain significance	0,00011
S15-02745	BAP1	chr3:52440906	C/T	SNV	1			COSM5025953	5,6	13,0	p.Glu200Lys	232	8	NM_004656,3	c.598G>A	missense	0,998	0,99(COSM5025953)		0,00288
S15-02745	BAP1	chr3:52443592	C/A	SNV	1			COSM6945443	6,5	10,0	p.Asp34Tyr	153	3	NM_004656,3	c.100G>T. c.-3304C>A	missense, unknown	1	0,98(COSM6945443)		0,0024
S15-02745	BRIP1	chr17:59770824	G/A	SNV	1			COSM6452095	6,2	28,0	p.Arg848Cys	449	18	NM_032043,2	c.2542C>T	missense	1	0,97(COSM6452095)	Uncertain significance	0,00001
S15-02745	CDH1	chr16:68842729	G/T	SNV	1			COSM1679222	9,3	15,0	p.Arg222Ile	162	5	NM_004360,4	c.665G>T	missense	1	0,99(COSM1679222)		0,00001
S15-02745	CTNNB1	chr3:41266148	A/G	SNV	1			COSM5760	12,5	19,0	p.Lys49Glu	152	3	NM_001904,3	c.145A>G	missense	0,212	0,98(COSM5760)		0,00001
S15-02745	EGFR	chr7:55211079	A/G	SNV	1	Hotspot	Gain-of-function	COSM1451536	7,5	23,0	p.Arg108Gly	307	3	NM_005228,4	c.322A>G	missense	1	0,73(COSM1451536)	Likely pathogenic	0,00003
S15-02745	FGFR2	chr10:123298225	C/T	SNV	1			COSM174986	9,9	21,0	p.Arg210Gln	212	6	NM_000141,4	c.629G>A	missense	1	1,00(COSM174986)		0,00001
S15-02745	KIT	chr4:55602887	G/A	SNV	1			COSM6926781	5,3	18,0	p.Gly866Glu	343	19	NM_000222,2	c.2597G>A	missense	1	0,99(COSM6926781)		0,00143
S15-02745	KRAS	chr12:25398284	CC/TC	SNV	1	Hotspot	Gain-of-function	COSM521	9,2	14,0	p.Gly12Asp	153	2	NM_033360,3	c.35G>A	missense	0,517	0,98(COSM521)	Pathogenic	0,00001
S15-02745	MLH1	chr3:37090086	C/T	SNV	1	Deleterious	Loss-of-function	COSM1692535	6,9	20,0	p.Arg659Ter	291	17	NM_000249,3	c.1975C>T	nonsense		0,97(COSM1692535)	Pathogenic	0,00002
S15-02745	MLH1	chr3:37042536	C/T	SNV	1	Deleterious	Loss-of-function	COSM29727	7,5	15,0	p.Arg100Ter	201	3	NM_000249,3	c.298C>T	nonsense		0,88(COSM29727)	Pathogenic	0,00008
S15-02745	MSH2	chr2:47637299	A/G	SNV	1			COSM9496203	7,1	23,0	p.Ile145Val	324	3	NM_000251,2	c.433A>G	missense	0	0,80(COSM9496203)	Uncertain significance	0,00001
S15-02745	MSH3	chr5:80074640	G/A	SNV	1	Deleterious	Loss-of-function	COSM3856618	5,3	17,0	p.Trp807Ter	323	17	NM_002439,4	c.2420G>A	nonsense		0,99(COSM3856618)		0,00175
S15-02745	MSH6	chr2:48028105	G/A	SNV	1			COSM7346757	6,6	13,1	p.Glu995Lys	200	4	NM_000179,2	c.2983G>A	missense	0,992	0,99(COSM7346757)	Uncertain significance	0,0007
S15-02745	MYC	chr8:128750803	C/G	SNV	1			COSM6209969	6,9	15,0	p.Leu114Val	216	2	NM_002467,5	c.340C>G	missense	0,855	0,96(COSM6209969)		0,00018
S15-02745	NF1	chr17:29665096	G/A	SNV	1			COSM1189438	14,6	29,0	p.Gly2253Glu	199	45	NM_001042492,2	c.6758G>A	missense	1	0,99(COSM1189437)	Uncertain significance	0,00001
S15-02745	PALB2	chr16:23646627	G/A	SNV	1	Deleterious	Loss-of-function	COSM6912572	8,6	16,0	p.Arg414Ter	187	4	NM_024675,3	c.1240C>T	nonsense		0,97(COSM6912572)	Pathogenic	0,00001
S15-02745	PIK3CA	chr3:178917544	G/A	SNV	1			COSM7339275	27,5	28,0	p.Arg140Gln	102	3	NM_006218,3	c.419G>A	missense	1	0,99(COSM7339275)		0,00001
S15-02745	PIK3CA	chr3:178936114	G/A	SNV	1			COSM37025	5,5	18,0	p.Trp552Ter	326	10	NM_006218,3	c.1656G>A	nonsense		0,99(COSM37025)		0,00079
S15-02745	POLD1	chr19:50909526	C/T	SNV	1			COSM6908588	6,0	16,0	p.Arg444Trp	266	11	NM_001256849,1	c.1330C>T	missense	1	0,94(COSM6908588)		0,00058
S15-02745	POLE	chr12:133219215	G/T	SNV	1			COSM6980999	6,5	19,0	p.Pro1610His	291	37	NM_006231,3	c.4829C>A	missense	0,034	1,00(COSM6980999)		0,00007
S15-02745	PTEN	chr10:89692980	A/G	SNV	1	Hotspot	Loss-of-function	COSM5144	13,5	33,0	p.Tyr155Cys	245	5	NM_000314,6	c.464A>G	missense	1	0,98(COSM5144)	Conflicting interpretations of pat	0,00001
S15-02745	PTEN	chr10:89717661	C/T	SNV	1			COSM7335257	5,9	11,0	p.Ser229Leu	187	7	NM_000314,6	c.686C>T	missense	0,042	0,97(COSM7335257)		0,00361
S15-02745	RAD51C	chr17:56787223	C/T	SNV	1	Deleterious	Loss-of-function	COSM212455	13,6	16,0	p.Arg237Ter	118	5	NM_058216,2	c.709C>T	nonsense		0,78(COSM212455)	Pathogenic	0,00001
S15-02745	SMAD4	chr18:48604665	G/A	SNV	1	Hotspot	Loss-of-function	COSM14193	11,8	20,0	p.Arg496His	170	12	NM_005359,5	c.1487G>A	missense	0,996	0,99(COSM14193)	Uncertain significance	0,00001
S15-02745	TP53	chr17:7578547	G/A	SNV	1			COSM45131	8,9	23,0	p.Pro128Leu	259	5	NM_000546,5	c.383C>T	missense	0,977	0,71(COSM45131)		0,00001
S15-02745	TP53	chr17:7578503	C/A	SNV	1			COSM44904	5,2	16,0	p.Val143Leu	310	5	NM_000546,5	c.427G>T	missense	0,401	0,94(COSM44904)	Uncertain significance	0,00278
S15-04504	CDKN2A	chr9:21971036	C/A	SNV	1	Hotspot	Loss-of-function	COSM13489	20,6	141,0	p.Asp108Tyr	685	2	NM_001195132,1	c.322G>T	missense	1	0,97(COSM12484),	Likely pathogenic	0,00001
S15-04504	KRAS	chr12:25398284	CC/CG	SNV	1	Hotspot	Gain-of-function	COSM518	16,7	169,0	p.Gly12Arg	1015	2	NM_033360,3	c.34G>C	missense	0,741	0,98(COSM1135366)	Pathogenic	0,00001
S15-05230	APC	chr5:112090658	G/A	SNV	1			COSM7347128	5,2	44,0	p.Arg24Gln	850	2	NM_000038,5	c.71G>A	missense	1	0,99 (COSM7347128)	Uncertain significance	0,00001
S15-05230	BRAF	chr7:140439694	C/T	SNV	1			COSM26447	5,7	42,0	p.Arg682Gln	736	17	NM_004333,5	c.2045G>A	missense	0,963	0,96 (COSM26447)		0,00001
S15-05230	CTNNB1	chr3:41274877	G/A	SNV	1			COSM327069	5,9	32,0	p.Arg376His	540	8	NM_001904,3	c.1127G>A	missense	1	1,00 (COSM327069)		0,00001
S15-05230	KRAS	chr12:25398284	CC/TC	SNV	1	Hotspot	Gain-of-function	COSM521	37,0	84,0	p.Gly12Asp	227	2	NM_033360,3	c.35G>A	missense	0,517	0,98 (COSM521)	Pathogenic	0,00001
S15-05230	MLH1	chr3:37067495	C/T	SNV	1			COSM29727	7,2	17,0	p.Pro469Leu	237	12	NM_000249,3	c.1406C>T	missense	0,1	0,75 (COSM25912)		0,00005
S15-05230	MLH1	chr3:37042536	C/T	SNV	1	Deleterious	Loss-of-function	COSM29727	5,1	10,0	p.Arg100Ter	197	3	NM_000249,3	c.298C>T	nonsense		0,88(COSM29727)	Pathogenic	0,01335
S15-05230	PMS2	chr7:6042130	G/A	SNV	1			COSM8012087	7,3	13,0	p.Ser164Phe	179	5	NM_000535,6	c.491C>T	missense	0,101	0,80 (COSM8012087)		0,00028
S15-05230	TP53	chr17:7577538	C/T	SNV	1	Hotspot	Loss-of-function	COSM10662	28,6	324,0	p.Arg248Gln	1134	7	NM_000546,5	c.743G>A	missense	1	0,98 (COSM10662)	Pathogenic	0,00001
S15-05501	AKT3	chr1:243809308	C/T	SNV	1			COSM9937035	21,1	36,0	p.Ala106Thr	171	4	NM_005465,4	c.316G>A	missense	0,567	0,99(COSM9937035)		0,00001
S15-05501	ALK	chr2:29430090	C/A	SNV	1			COSM5575702	25,7	26,0	p.Trp1295Cys	101	26	NM_004304,4	c.3885G>T	missense	1	0,96(COSM5575702)		0,00001
S15-05501	APC	chr5:112103020	C/T	SNV	1			COSM9860758	18,3	62,0	p.Pro119Ser	339	4	NM_000038,5	c.355C>T	missense	0,002	0,91(COSM9860758)		0,00001
S15-05501	APC	chr5:112128191	C/T	SNV	1	Deleterious	Loss-of-Function	COSM13130	16,8	62,0	p.Arg232Ter	369	7	NM_000038,5	c.694C>T	nonsense		0,89(COSM13130)	Pathogenic	0,00001
S15-05501	APC	chr5:112175521	TGG/TGA	SNV	1			COSM27577	46,5	53,0	p.Val1414Ile	114	16	NM_000038,5	c.4240G>A	missense	0	0,84(COSM27577)		0,00001
S15-05501	APC	chr5:112151238	C/A	SNV	1			COSM6854129	7,3	26,0	p.Ser294Tyr	355	9	NM_000038,5	c.881C>A	missense	0,874	0,96(COSM6854129)		0,00001
S15-05501	ATM	chr11:108123557	C/T	SNV	1			COSM6928996	13,2	97,0	p.Leu606Phe	736	12	NM_000051,3	c.1816C>T	missense	0,047	0,77(COSM6928996)		0,00001
S15-05501	ATM	chr11:108100002	C/T	SNV	1	Deleterious	Loss-of-Function	COSM4745867	16,5	85,0	p.Gln95Ter	514	4	NM_000051,3	c.283C>T	nonsense		0,97(COSM4745867)	Pathogenic	0,00001
S15-05501	ATM	chr11:108199848	A/G	SNV	1			COSM5008060	27,2	68,0	p.Gln2397Arg	250	49	NM_000051,3	c.7190A>G	missense	1	0,99(COSM5008060)	Uncertain significance	0,00001
S15-05501	ATM	chr11:108173675	G/A	SNV	1	Deleterious	Loss-of-Function	COSM4551819	10,3	55,0	p.Trp1805Ter	532	36	NM_000051,3	c.5415G>A	nonsense		0,99(COSM4551819)		0,00001

ID	Genes	Locus	Genotype	Type	Length	Oncomine Variant Class	Oncomine Gene Class	Variant ID	% Frequency	Mutated Alleles	Amino Acid Change	Coverage	Exon	Transcript	Coding	Variant Effect	PolyPhen	FATHMM	ClinVar	p-value
S15-05501	ATM	chr11:108213964	C/T	SNV	1	Deleterious	Loss-of-Function	COSM3733461	11,4	49,0	p.Gln2762Ter	430	57	NM_000051,3	c.8284C>T	nonsense		0,99(COSM3733461)		0,00001
S15-05501	ATM	chr11:108155093	C/T	SNV	1			COSM4170229	11,7	34,0	p.Pro1296Ser	290	26	NM_000051,3	c.3886C>T	missense	1	0,99(COSM4170229)	Uncertain significance	0,00001
S15-05501	ATM	chr11:108119806	G/T	SNV	1			COSM6854116	9,4	29,0	p.Gln404His	309	9	NM_000051,3	c.1212G>T	missense	0,001	0,82(COSM6854116)		0,00001
S15-05501	ATM	chr11:108235935	C/T	SNV	1	Deleterious	Loss-of-Function	COSM428365	6,3	27,0	p.Arg2993Ter	431	62	NM_000051,3	c.8977C>T	nonsense		0,97(COSM428365)	Pathogenic/Likely pathogenic	0,00001
S15-05501	ATM	chr11:108141997	C/T	SNV	1			COSM5546583	7,0	22,0	p.Arg981Cys	316	20	NM_000051,3	c.2941C>T	missense	0,999	0,99(COSM5546583)	Uncertain significance	0,00001
S15-05501	BRAF	chr7:140481417	C/T	SNV	1	Hotspot	Gain-of-Function	COSM449	12,8	80,0	p.Gly464Glu	626	11	NM_004333,5	c.1391G>A	missense	1	0,99(COSM449)	Pathogenic/Likely pathogenic	0,00001
S15-05501	BRAF	chr7:140481471	G/A	SNV	1			COSM7448237	5,8	17,0	p.Ser446Leu	293	11	NM_004333,5	c.1337C>T	missense	0,274	0,99(COSM7448237)		0,00061
S15-05501	CDH1	chr16:68846144	C/A	SNV	1			COSM6624567	8,4	76,0	p.Pro372His	906	8	NM_004360,4	c.1115C>A	missense	0,999	0,70(COSM6624567)		0,00001
S15-05501	CHEK2	chr22:29092903	C/A	SNV	1			COSM6526453	9,2	37,0	p.Asp361Tyr	403	10	NM_007194,4	c.1081G>T	missense	0,006	0,86(COSM6526453)	Uncertain significance	0,00001
S15-05501	CHEK2	chr22:29091193	G/A	SNV	1	Deleterious	Loss-of-Function	COSM9180242	5,6	10,0	p.Gln433Ter	178	12	NM_007194,4	c.1297C>T	nonsense		0,85(COSM9180242)	Pathogenic	0,00672
S15-05501	CTNNB1	chr3:41266034	G/A	SNV	1			COSM6084	6,3	63,0	p.Asp11Asn	995	3	NM_001904,3	c.31G>A	missense	0,167	0,98(COSM6084)		0,00001
S15-05501	CTNNB1	chr3:41266131	C/T	SNV	1			COSM5699	12,6	14,0	p.Ala43Val	111	3	NM_001904,3	c.128C>T	missense	0,038	0,98(COSM5699)		0,00001
S15-05501	EGFR	chr7:55259512	G/A	SNV	1			COSM250050	5,5	41,0	p.Gly857Glu	752	21	NM_005228,4	c.2570G>A	missense	1	0,99(COSM250050)		0,00001
S15-05501	EGFR	chr7:55266424	G/A	SNV	1			COSM9515585	5,6	27,0	p.Glu906Lys	484	23	NM_005228,4	c.2716G>A	missense	1	0,99(COSM9515585)		0,00006
S15-05501	EGFR	chr7:55242480	AAA/GAA	SNV	1			COSM85993	11,3	25,0	p.Lys754Glu	222	19	NM_005228,4	c.2260A>G	missense	0,748	0,99(COSM85993)	Uncertain significance	0,00001
S15-05501	FANCD2	chr3:10133883	C/T	SNV	1			COSM9804315	29,0	29,0	p.Leu1266Phe	100	38	NM_033084,4	c.3796C>T. c.*44-10668G>A	missense, unknown	0,951	0,94(COSM9804315)		0,00001
S15-05501	GNAQ	chr9:80343561	G/A	SNV	1			COSM4605880	8,2	12,0	p.Ala253Val	146	6	NM_002072,4	c.758C>T	missense	0,997	0,99(COSM4605880)		0,00015
S15-05501	MSH2	chr2:47635554	C/T	SNV	1	Deleterious	Loss-of-Function	COSM8497017	7,3	39,0	p.Gln76Ter	532	2	NM_000251,2	c.226C>T	nonsense		0,91(COSM8497017)	Pathogenic	0,00001
S15-05501	MSH6	chr2:48027853	C/T	SNV	1	Deleterious	Loss-of-Function	COSM26816	38,8	94,0	p.Arg911Ter	242	4	NM_000179,2	c.2731C>T	nonsense		0,95(COSM26816)	Pathogenic	0,00001
S15-05501	NF1	chr17:29585383	C/T	SNV	1	Deleterious	Loss-of-Function	COSM4833971	80,9	110,0	p.Gln1399Ter	136	32	NM_001042492,2	c.4195C>T	nonsense		0,98(COSM4833971)	Pathogenic	0,00001
S15-05501	NF1	chr17:29528489	C/T	SNV	1	Deleterious	Loss-of-Function	COSM27353	11,2	36,0	p.Arg416Ter	322	11	NM_001042492,2	c.1246C>T	nonsense		0,90(COSM1217153)	Pathogenic	0,00001
S15-05501	NF1	chr17:29701036	G/A	SNV	1			COSM6950788	7,3	21,0	p.Asp2795Asn	286	58	NM_001042492,2	c.8383G>A	missense	0,999	0,98(COSM6950788)		0,00001
S15-05501	NF1	chr17:29657444	A/G	SNV	1			COSM3378123	7,4	19,0	p.Thr1914Ala	256	39	NM_001042492,2	c.5740A>G	missense	0,118	0,99(COSM3378123)		0,00001
S15-05501	NRAS	chr1:115251236	G/A	SNV	1			COSM7590891	12,4	69,0	p.Arg164Cys	556	5	NM_002524,4	c.490C>T	missense	0,989	0,98(COSM7590891)		0,00001
S15-05501	PIK3CA	chr3:178952074	G/A	SNV	1	Hotspot	Gain-of-Function	COSM29313	26,2	74,0	p.Met1043Ile	283	21	NM_006218,3	c.3129G>A	missense	0,138	0,97(COSM29313)	Likely pathogenic	0,00001
S15-05501	PIK3CA	chr3:178943785	C/T	SNV	1			COSM1041507	7,2	48,0	p.Arg818Cys	669	17	NM_006218,3	c.2452C>T	missense	0,992	0,91(COSM1041507)		0,00001
S15-05501	PIK3CA	chr3:178936091	G/A	SNV	1	Hotspot	Gain-of-Function	COSM763	5,3	31,0	p.Glu545Lys	581	10	NM_006218,3	c.1633G>A	missense	0,991	0,97(COSM763)	Pathogenic/Likely pathogenic	0,001
S15-05501	PIK3CA	chr3:178942555	A/G	SNV	1			COSM7455366	9,5	22,0	p.Ile788Val	232	16	NM_006218,3	c.2362A>G	missense	0,024	0,98(COSM7455366)		0,00001
S15-05501	PMS2	chr7:6038848	C/T	SNV	1			COSM6971277	21,0	31,0	p.Arg199His	148	6	NM_000535,6	c.596G>A	missense	1	0,99(COSM6971277)	Uncertain significance	0,00001
S15-05501	POLD1	chr19:50909524	G/A	SNV	1			COSM6938696	20,2	53,0	p.Arg443Gln	263	11	NM_001256849,1	c.1328G>A	missense	0,727	0,99(COSM6938696)		0,00001
S15-05501	POLE	chr12:133249853	G/A	SNV	1			COSM5031063	27,4	29,0	p.Thr457Met	106	14	NM_006231,3	c.1370C>T	missense	0,996	1,00(COSM5031,00063)	Uncertain significance	0,00001
S15-05501	PTEN	chr10:89685281	C/T	SNV	1			COSM87316	8,6	48,0	p.Ser59Leu	558	3	NM_000314,6	c.176C>T	missense	0,068	0,99(COSM87316)		0,00001
S15-05501	RB1	chr13:49050906	G/T	SNV	1	Deleterious	Loss-of-Function	COSM432458	5,7	12,0	p.Glu864Ter	211	25	NM_000321,2	c.2590G>T	nonsense		0,95(COSM432458)		0,00334
S15-05501	TP53	chr17:7574003	G/A	SNV	1	Deleterious	Loss-of-Function	COSM11073	12,8	139,0	p.Arg342Ter	1085	10	NM_000546,5	c.1024C>T	nonsense		0,73(COSM11073)	Pathogenic	0,00001
S15-05501	TP53	chr17:7578518	C/T	SNV	1	Hotspot	Loss-of-Function	COSM44821	37,8	90,0	p.Ala138Thr	238	5	NM_000546,5	c.412G>A	missense	0,998	1,00(COSM44821,00)		0,00001
S15-05501	TP53	chr17:7577093	C/T	SNV	1	Hotspot	Loss-of-Function	COSM44338	16,7	50,0	p.Arg282Gln	300	8	NM_000546,5	c.845G>A	missense	1	0,98(COSM44338)	Conflicting interpretations of pat	0,00001
S15-05501	TP53	chr17:7577121	G/A	SNV	1	Hotspot	Loss-of-Function	COSM10659	16,6	50,0	p.Arg273Cys	301	8	NM_000546,5	c.817C>T	missense	1	0,98(COSM10659)	Conflicting interpretations of pat	0,00001
S15-05501	TP53	chr17:7577556	C/T	SNV	1	Hotspot	Loss-of-Function	COSM10646	5,3	48,0	p.Cys242Tyr	913	7	NM_000546,5	c.725G>A	missense	1	0,99(COSM10646)	Pathogenic	0,0001
S15-05501	TP53	chr17:7578263	G/A	SNV	1	Deleterious	Loss-of-Function	COSM10705	5,6	32,0	p.Arg196Ter	570	6	NM_000546,5	c.586C>T	nonsense		0,96(COSM10705)	Pathogenic	0,00036
S15-05501	TP53	chr17:7578253	C/A	SNV	1			COSM44140	6,9	29,0	p.Gly199Val	423	6	NM_000546,5	c.596G>T	missense	1	0,99(COSM44140)	Likely pathogenic	0,00001
S15-05501	VHL	chr3:10191632	C/T	SNV	1	Deleterious	Loss-of-Function	COSM18382	31,1	88,0	p.Gln209Ter	283	3	NM_000551,3	c.625C>T	nonsense		0,81(COSM18382)		0,00001
S15-06320	CDKN2A	chr9:21971111	G/C	SNV	1	Hotspot	Loss-of-function	COSM4388670	17,8	295,0	p.His83Asp	1662	2	NM_001195132,1	c.247C>G	missense	1	0,98(COSM4388670)	Likely pathogenic	0,00001
S15-06320	KRAS	chr12:25380277	GA/TT	MNV	2	Hotspot	Gain-of-function	COSM549	11,4	226,9	p.Gln61Lys	1994	3	NM_033360,3	c.180_181delTCinsAA	missense	0,039	1,00(COSM87298)		0,00001
S15-06320	TP53	chr17:7577121	G/A	SNV	1	Hotspot	Loss-of-function	COSM10659	8,2	164,0	p.Arg273Cys	2000	8	NM_000546,5	c.817C>T	missense	1	0,98(COSM10659)	Conflicting interpretations of pat	0,00001
S15-06750	CDKN2A	chr9:21971111	G/C	SNV	1	Hotspot	Loss-of-function	COSM4388670	15,1	302,1	p.His83Asp	1998	2	NM_001195132,1	c.247C>G	missense	1	0,98(COSM4388670)	Likely pathogenic	0,00001
S15-06750	KRAS	chr12:25398284	CC/TC	SNV	1	Hotspot	Gain-of-function	COSM521	31,7	298,0	p.Gly12Asp	940	2	NM_033360,3	c.35G>A	missense	0,517	0,98 (COSM521)	Pathogenic	0,00001
S15-06750	TP53	chr17:7577556	C/T	SNV	1	Hotspot	Loss-of-function	COSM10646	32,6	605,0	p.Cys242Tyr	1858	7	NM_000546,5	c.725G>A	missense	1	0,99(COSM10646)	Pathogenic	0,00001
S15-06780	APC	chr5:112137039	G/A	SNV	1			COSM9106966	5,7	11,0	p.Gly265Arg	194	8	NM_000038,5	c.793G>A	missense	0,704	0,97(COSM9106966)		0,00474
S15-06780	APC	chr5:112176425	G/A	SNV	1			COSM6475471	7,1	10,0	p.Glu1712Lys	141	16	NM_000038,5	c.5134G>A	missense	0,02	0,94(COSM6475471)		0,00132
S15-06780	ARID2	chr12:46254606	C/T	SNV	1			COSM7340179	5,7	55,0	p.Pro1599Leu	974	16	NM_152641,3	c.4796C>T	missense	0,22	0,97(COSM7340179)		0,00001
S15-06780	ARID2	chr12:46245688	G/A	SNV	1			COSM6604638	5,9	20,0	p.Arg1261His	338	15	NM_152641,3	c.3782G>A	missense	1	0,99(COSM6604638)		0,00019
S15-06780	ARID2	chr12:46125084	C/T	SNV	1	Deleterious	Loss-of-Function	COSM5704452	6,5	15,0	p.Gln91Ter	231	3	NM_152641,3	c.271C>T	nonsense		0,97(COSM5704452)		0,00037
S15-06780	ATM	chr11:108186602	G/A	SNV	1			COSM5753585	5,1	32,0	p.Gly2020Asp	632	41	NM_000051,3	c.6059G>A	missense	1	0,99(COSM5753585)		0,00009
S15-06780	ATM	chr11:108186608	G/A	SNV	1			COSM4800231	5,1	32,0	p.Gly2022Asp	632	41	NM_000051,3	c.6065G>A	missense	1	0,99(COSM4800231)		0,00009
S15-06780	ATM	chr11:108180999	G/A	SNV	1			COSM1739752	5,7	20,0	p.Glu1959Lys	352	39	NM_000051,3	c.5875G>A	missense	1	1,00(COSM1,00739752)		0,00032
S15-06780	ATM	chr11:108121777	G/A	SNV	1			COSM8550543	7,0	15,0	p.Gly529Arg	214	10	NM_000051,3	c.1585G>A	missense	1	0,99(COSM8550543)		0,00016
S15-06780	BRIP1	chr17:59760893	C/T	SNV	1			COSM7652556	6,0	18,0	p.Glu1172Lys	300	20	NM_032043,2	c.3514G>A	missense	0,001	0,87(COSM7652556)		0,00031
S15-06780	CDH1	chr16:68847364	C/T	SNV	1			COSM3511252	5,7	22,0	p.Pro429Leu	383	9	NM_004360,4	c.1286C>T	missense	0,99	0,99(COSM3511252)	Uncertain significance	0,00016
S15-06780	FANCD2	chr3:10127575	G/A	SNV	1			COSM7154912	6,6	19,0	p.Glu1102Lys	287	33	NM_033084,4	c.3304G>A. c.*44-4360C>T	missense, unknown	0,818	O,84(COSM7154912)		0,00008
S15-06780	FGFR2	chr10:123243305	C/T	SNV	1			COSM1730961	5,4	12,0	p.Met736Ile	224	17	NM_000141,4	c.2208G>A	missense	1	0,97(COSM1730961)		0,00546
S15-06780	FGFR2	chr10:123298199	C/T	SNV	1			COSM29832	6,0	11,0	p.Glu219Lys	184	6	NM_000141,4	c.655G>A	missense	0,932	0,99(COSM29832)		0,00319
S15-06780	MSH2	chr2:47703556	G/A	SNV	1			COSM9133425	5,1	11,0	p.Val686Ile	215	13	NM_000251,2	c.2056G>A	missense	0,955	0,99(COSM9133425)		0,00978
S15-06780	MSH6	chr2:48027089	C/T	SNV	1			COSM6948227	5,6	12,0	p.Pro656Leu	215	4	NM_000179,2	c.1967C>T	missense	1	0,99(COSM6948227)		0,0039
S15-06780	NF1	chr17:29664545	C/T	SNV	1			COSM9207612	5,4	34,0	p.Thr2196Ile	628	43	NM_001042492,2	c.6587C>T	missense	0,033	0,98(COSM9207612)		0,00002
S15-06780	NF1	chr17:29663783	C/T	SNV	1			COSM1686252	5,1	32,0	p.Ser2093Phe	629	42	NM_001042492,2	c.6278C>T	missense	0,99	0,98(COSM1686252)		0,00009
S15-06780	NF1	chr17:29665080	G/A	SNV	1			COSM6915711	5,7	16,0	p.Ala2248Thr	279	45	NM_001042492,2	c.6742G>A	missense	1	0,99(COSM6915711)		0,00094
S15-06780	NF1	chr17:29562729	C/T	SNV	1			COSM5609530	5,9	15,0	p.Ser1270Phe	253	28	NM_001042492,2	c.3809C>T	missense	0,995	0,98(COSM5609530)		0,0009
S15-06780	POLE	chr12:133212606	G/A	SNV	1			COSM6282424	7,1	14,0	p.His1895Tyr	196	42	NM_006231,3	c.5683C>T	missense	0,029	0,99(COSM6282424)		0,00021
S15-06780	RAD51D	chr17:33428031	C/T	SNV	1			COSM4563742	5,2	14,0	p.Asp198Asn	268	7	NR_037714,1	c.592G>A	missense	0,988	0,90(COSM4563742)		0,00404
S15-06780	RB1	chr13:48954220	G/A	SNV	1			COSM1050	8,9	10,0	p.Ser474Asn	112	15	NM_000321,2	c.1421G>A	missense	1	0,98(COSM1050)		0,00025
S15-06780	TP53	chr17:7578406	C/T	SNV	1	Hotspot	Loss-of-Function	COSM10648	8,5	25,0	p.Arg175His	294	5	NM_000546,5	c.524G>A	missense	0,632	0,99(COSM10648	Pathogenic	0,00001
S16-01912	APC	chr5:112175207	TA/AA	SNV	1			COSM26697	42,2	831,9	p.Ile1307Lys	1970	16	NM_000038,5	c.3920T>A	missense	0,012	0,94(COSM26697)	Conflicting interpretations of pat	0,00001
S16-01912	CDH1	chr16:68847375	G/A	SNV	1			COSM239247	55,4	1107,0	p.Asp433Asn	1999	9	NM_004360,4	c.1297G>A	missense	0,135	0,86(COSM239247)	Conflicting interpretations of pat	0,00001
S16-01912	KIT	chr4:55593464	A/C	SNV	1			COSM28026	51,3	1019,0	p.Met541Leu	1988	10	NM_000222,2	c.1621A>C	missense	0,009	0,74(COSM28026)	Likely benign	0,00001
S16-01912	KRAS	chr12:25398284	CC/TC	SNV	1	Hotspot	Gain-of-function	COSM521	18,8	227,0	p.Gly12Asp	1205	2	NM_033360,3	c.35G>A	missense	0,517	0,98(COSM521)	Pathogenic	0,00001
S16-01912	MYC	chr8:128750803	C/G	SNV	1			COSM6209969	6,4	85,1	p.Leu114Val	1325	2	NM_002467,5	c.340C>G	missense	0,855	0,96(COSM6209969)		0,00001
S16-01912	TP53	chr17:7578416	C/A	SNV	1	Hotspot	Loss-of-function	COSM44240	15,8	57,0	p.Val172Phe	361	5	NM_000546,5	c.514G>T	missense	1	0,99(COSM44240)	Likely pathogenic	0,00001
S16-02610	APC	chr5:112102095	G/A	SNV	1			COSM5356439	15,3	17,0	p.Glu70Lys	111	3	NM_000038,5	c.208G>A	missense	0,99	0,99(COSM5356439)		0,00001

ID	Genes	Locus	Genotype	Type	Length	Oncomine Variant Class	Oncomine Gene Class	Variant ID	% Frequency	Mutated Alleles	Amino Acid Change	Coverage	Exon	Transcript	Coding	Variant Effect	PolyPhen	FATHMM	ClinVar	p-value
S16-02610	MSH3	chr5:80063775	G/T	SNV	1			COSM5043262	17,2	20,0	p.Leu640Phe	116	14	NM_002439,4	c.1920G>T	missense	0,074	0,88(COSM5043262)		0,00001
S16-05115	ALK	chr2:29443675	C/T	SNV	1			COSM6226578	5,8	107,9	p.Arg1181His	1867	23	NM_004304,4	c.3542G>A	missense	0,966	0,98(COSM6226578)	Uncertain significance	0,00001
S16-05115	APC	chr5:112174580	G/T	SNV	1	Deleterious	Loss-of-function	COSM5009571	6,7	90,0	p.Glu1097Ter	1343	16	NM_000038,5	c.3289G>T	nonsense		0,97(COSM5009571)		0,00001
S16-05115	APC	chr5:112178211	C/T	SNV	1			COSM1183194	5,8	25,0	p.Ser2307Leu	432	16	NM_000038,5	c.6920C>T	missense	0,999	0,93(COSM1183194)		0,00006
S16-05115	ARID2	chr12:46287315	C/T	SNV	1	Deleterious	Loss-of-function	COSM1628614	5,7	106,0	p.Arg1754Ter	1869	19	NM_152641,3	c.5260C>T	nonsense		0,90(COSM1628614)		0,00001
S16-05115	ARID2	chr12:46244924	G/A	SNV	1			COSM8741210	6,4	95,0	p.Met1006Ile	1496	15	NM_152641,3	c.3018G>A	missense	0,801	0,93(COSM8741210)		0,00001
S16-05115	ARID2	chr12:46230572	G/A	SNV	1			COSM939496	9,1	87,0	p.Arg274Gln	955	8	NM_152641,3	c.821G>A	missense	1	1,00(COSM939496)		0,00001
S16-05115	ARID2	chr12:46231337	G/T	SNV	1	Deleterious	Loss-of-function	COSM131488	11,6	62,0	p.Glu393Ter	533	10	NM_152641,3	c.1177G>T	nonsense		0,99(COSM131488)		0,00001
S16-05115	ATM	chr11:108186751	G/T	SNV	1	Deleterious	Loss-of-function	COSM4411297	6,0	50,0	p.Glu2037Ter	836	42	NM_000051,3	c.6109G>T	nonsense		0,99(COSM4411297)		0,00001
S16-05115	EGFR	chr7:55249022	G/A	SNV	1			COSM13006	6,4	57,0	p.Val774Met	888	20	NM_005228,4	c.2320G>A	missense	1	0,97(COSM13006)		0,00001
S16-05115	EGFR	chr7:55249157	G/A	SNV	1			COSM7410341	6,1	42,0	p.Val819Met	685	20	NM_005228,4	c.2455G>A	missense	1	0,96(COSM7410341)		0,00001
S16-05115	FANCD2	chr3:10094114	G/A	SNV	1			COSM249507	8,2	80,0	p.Arg530Gln	972	18	NM_033084,4	c.1589G>A	missense	1	0,94(COSM249507)		0,00001
S16-05115	GNA11	chr19:3110285	G/A	SNV	1			COSM6672102	5,5	109,1	p.Arg92Gln	1998	2	NM_002067,4	c.275G>A	missense	0,008	0,97(COSM6672102)		0,00001
S16-05115	KIT	chr4:55598137	G/T	SNV	1			COSM1651756	5,5	57,9	p.Lys778Asn	1046	16	NM_000222,2	c.2334G>T	missense	1	0,97(COSM1651756)		0,00001
S16-05115	KRAS	chr12:25398284	CC/TC	SNV	1	Hotspot	Gain-of-function	COSM521	7,7	36,0	p.Gly12Asp	470	2	NM_033360,3	c.35G>A	missense	0,517	0,98(COSM521)	Pathogenic	0,00001
S16-05115	MLH1	chr3:37042530	G/T	SNV	1			COSM6097410	5,7	38,0	p.Gly98Cys	667	3	NM_000249,3	c.292G>T	missense	1	0,99(COSM6097410)		0,00001
S16-05115	MSH2	chr2:47630442	G/A	SNV	1			COSM9113785	6,5	35,0	p.Asp38Asn	539	1	NM_000251,2	c.112G>A	missense	0,778	0,97(COSM9113785)		0,00001
S16-05115	MSH3	chr5:79974803	C/T	SNV	1			COSM1695912	6,7	65,0	p.Arg411Cys	975	8	NM_002439,4	c.1231C>T	missense	1	0,99(COSM1695912)		0,00001
S16-05115	MSH3	chr5:80160759	C/T	SNV	1			COSM9372944	6,3	55,0	p.Pro1043Leu	867	22	NM_002439,4	c.3128C>T	missense	0,125	0,82(COSM9372944)		0,00001
S16-05115	NF1	chr17:29665110	C/T	SNV	1	Deleterious	Loss-of-function	COSM215676	14,1	76,0	p.Arg2258Ter	540	45	NM_001042492,2	c.6772C>T	nonsense		0,94(COSM215676)	Pathogenic	0,00001
S16-05115	NF1	chr17:29562981	C/T	SNV	1	Deleterious	Loss-of-function	COSM24478	5,9	45,0	p.Arg1306Ter	759	29	NM_001042492,2	c.3916C>T	nonsense		0,91(COSM24478)	Pathogenic	0,00001
S16-05115	NF1	chr17:29559809	C/T	SNV	1			COSM96330	7,2	42,0	p.Arg1136Trp	580	26	NM_001042492,2	c.3406C>T	missense	0,993	0,96(COSM96330)		0,00001
S16-05115	PIK3CA	chr3:178951920	G/A	SNV	1			COSM249138	15,5	65,0	p.Arg992Gln	420	21	NM_006218,3	c.2975G>A	missense	0,999	0,99(COSM249138)		0,00001
S16-05115	POLD1	chr19:50918831	G/A	SNV	1			COSM6936628	6,1	31,0	p.Val901Met	510	21	NM_001256849,1	c.2701G>A	missense	1	0,96(COSM6936628)		0,00001
S16-05115	RB1	chr13:48881512	G/A	SNV	1	Deleterious	Loss-of-function	COSM13406	7,0	67,0	p.Trp78Ter	964	2	NM_000321,2	c.234G>A	nonsense		0,79(COSM13406)		0,00001
S16-05115	TP53	chr17:7578263	G/A	SNV	1	Deleterious	Loss-of-function	COSM10705	5,3	54,0	p.Arg196Ter	1018	6	NM_000546,5	c.586C>T	nonsense		0,96(COSM10705)	Pathogenic	0,00003
S16-05330	APC	chr5:112151261	C/T	SNV	1	Deleterious	Loss-of-function	COSM13862	11,4	53,0	p.Arg302Ter	467	9	NM_000038,5	c.904C>T	nonsense		0,97(COSM13862)	Pathogenic	0,00001
S16-05330	APC	chr5:112175457	C/A	SNV	1			COSM6940327	6,4	27,0	p.Ser1389Tyr	420	16	NM_000038,5	c.4166C>A	missense	1	0,97(COSM6940327)		0,00001
S16-05330	APC	chr5:112173995	G/T	SNV	1	Deleterious	Loss-of-function	COSM1178881	7,1	25,0	p.Glu902Ter	354	16	NM_000038,5	c.2704G>T	nonsense		1,00(COSM1178881)		0,00001
S16-05330	ARID2	chr12:46230679	C/T	SNV	1			COSM4041985	23,0	88,0	p.Arg310Cys	383	8	NM_152641,3	c.928C>T	missense	1	0,98(COSM4041985)		0,00001
S16-05330	ARID2	chr12:46287315	C/T	SNV	1	Deleterious	Loss-of-function	COSM1628614	10,5	46,0	p.Arg1754Ter	440	19	NM_152641,3	c.5260C>T	nonsense		0,90(COSM1628614)		0,00001
S16-05330	ATM	chr11:108115594	C/T	SNV	1	Deleterious	Loss-of-function	COSM1506643	38,8	107,0	p.Arg248Ter	276	7	NM_000051,3	c.742C>T	nonsense		0,95(COSM1506643)	Pathogenic/Likely pathogenic	0,00001
S16-05330	ATM	chr11:108121493	C/T	SNV	1			COSM6935823	23,2	68,0	p.Pro434Leu	293	10	NM_000051,3	c.1301C>T	missense	0,014	0,91(COSM6935823)		0,00001
S16-05330	CDKN2A	chr9:21971108	C/A	SNV	1	Hotspot	Loss-of-function	COSM13299	14,5	42,0	p.Asp84Tyr	289	2	NM_001195132,1	c.250G>T	missense	1	0,97(COSM13299)	Uncertain significance	0,00001
S16-05330	CTNNB1	chr3:41267237	G/A	SNV	1			COSM6854098	5,1	95,1	p.Arg274His	1868	6	NM_001904,3	c.821G>A	missense	1	0,98(COSM6854098)		0,00001
S16-05330	EGFR	chr7:55242480	CCG/CTG	SNV	1			COSM1168015	5,9	46,0	p.Pro753Leu	775	19	NM_005228,4	c.2258C>T	missense	0,995	0,99(COSM1168015)		0,00001
S16-05330	GNAQ	chr9:80412470	C/T	SNV	1			COSM6342236	17,8	86,0	p.Glu191Lys	482	4	NM_002072,4	c.571G>A	missense	1	0,98(COSM6342236)		0,00001
S16-05330	KIT	chr4:55603365	G/T	SNV	1			COSM5784409	19,2	51,0	p.Trp907Cys	266	20	NM_000222,2	c.2721G>T	missense	1	1,00(COSM5784409)		0,00001
S16-05330	KRAS	chr12:25380309	G/A	SNV	1			COSM6006382	10,4	51,0	p.Thr50Ile	490	3	NM_033360,3	c.149C>T	missense	0,022	0,99(COSM6006382)		0,00001
S16-05330	KRAS	chr12:25398284	CC/TC	SNV	1	Hotspot	Gain-of-function	COSM521	30,6	34,0	p.Gly12Asp	111	2	NM_033360,3	c.35G>A	missense	0,517	0,98(COSM521)	Pathogenic	0,00001
S16-05330	MLH1	chr3:37048546	C/T	SNV	1	Deleterious	Loss-of-function	COSM8199824	13,7	126,0	p.Gln149Ter	917	5	NM_000249,3	c.445C>T	nonsense		0,99(COSM8199824)	Pathogenic	0,00001
S16-05330	MSH2	chr2:47672695	C/T	SNV	1	Deleterious	Loss-of-function	COSM3185924	13,0	52,0	p.Gln429Ter	400	8	NM_000251,2	c.1285C>T	nonsense		0,99(COSM3185924)	Pathogenic	0,00001
S16-05330	MSH2	chr2:47635624	G/T	SNV	1			COSM6923715	14,3	29,0	p.Arg99Ile	203	2	NM_000251,2	c.296G>T	missense	1	0,99(COSM6923715)		0,00001
S16-05330	MSH6	chr2:48028048	C/T	SNV	1			COSM6475181	11,7	15,0	p.Arg976Cys	128	4	NM_000179,2	c.2926C>T	missense	1	0,98(COSM6475181)	Conflicting interpretations of pat	0,00001
S16-05330	NF1	chr17:29562750	G/T	SNV	1			COSM7379433	6,6	49,0	p.Gly1277Val	739	28	NM_001042492,2	c.3830G>T	missense	0,997	0,99(COSM7379433)	Uncertain significance	0,00001
S16-05330	NF1	chr17:29701088	G/A	SNV	1			COSM4560429	34,5	48,0	p.Arg2812Gln	139	58	NM_001042492,2	c.8435G>A	missense	0,994	0,99(COSM4560429)		0,00001
S16-05330	PIK3CA	chr3:178952048	G/A	SNV	1	Hotspot	Gain-of-function	COSM27375	6,5	24,0	p.Ala1035Thr	371	21	NM_006218,3	c.3103G>A	missense	1	0,99(COSM27375)		0,00024
S16-05330	PMS2	chr7:6038813	G/A	SNV	1	Deleterious	Loss-of-function	COSM6751159	11,6	28,0	p.Arg211Ter	242	6	NM_000535,6	c.631C>T	nonsense		0,92(COSM6751159)	Pathogenic	0,00001
S16-05330	POLD1	chr19:50916702	G/A	SNV	1			COSM4444684	7,4	50,0	p.Arg725His	675	18	NM_001256849,1	c.2174G>A	missense	1	0,98(COSM4444684)		0,00001
S16-05330	POLE	chr12:133234459	G/A	SNV	1	Deleterious	Loss-of-function	COSM5881470	10,0	27,0	p.Arg1125Ter	269	27	NM_006231,3	c.3373C>T	nonsense		0,99(COSM5881470)	Uncertain significance	0,00001
S16-05330	RB1	chr13:48936983	C/T	SNV	1	Deleterious	Loss-of-function	COSM878	18,9	53,0	p.Arg251Ter	281	8	NM_000321,2	c.751C>T	nonsense		0,82(COSM878)	Pathogenic	0,00001
S16-05330	TP53	chr17:7578550	G/T	SNV	1	Hotspot	Loss-of-function	COSM43970	7,7	68,0	p.Ser127Tyr	879	5	NM_000546,5	c.380C>A	missense	1	1,00(COSM43970)		0,00001
S16-05330	VHL	chr3:10191566	G/A	SNV	1			COSM1757302	17,3	28,0	p.Asp187Asn	162	3	NM_000551,3	c.559G>A	missense	0,999	0,92(COSM1757302)		0,00001
S16-09088	ALK	chr2:29445439	C/T	SNV	1			COSM3962765	5,2	15,0	p.Glu1132Lys	291	21	NM_004304,4	c.3394G>A	missense	0,977	0,99(COSM3962765)		0,00351
S16-09088	APC	chr5:112176413	G/A	SNV	1			COSM9495228	6,2	18,0	p.Val1708Ile	291	16	NM_000038,5	c.5122G>A	missense	0	0,92(COSM9495228)		0,00021
S16-09088	CDH1	chr16:68867274	G/A	SNV	1			COSM972806	6,6	13,0	p.Glu841Lys	196	16	NM_004360,4	c.2521G>A	missense	0,999	0,97(COSM972806)	Uncertain significance	0,00064
S16-09088	FANCD2	chr3:10085242	G/A	SNV	1			COSM8308097	8,0	11,0	p.Arg355Lys	137	13	NM_033084,4	c.1064G>A	missense	0,725	0,96(COSM8308097)		0,00031
S16-09088	KIT	chr4:55602952	C/T	SNV	1			COSM6919154	7,8	16,0	p.Arg888Trp	204	19	NM_000222,2	c.2662C>T	missense	1	0,88(COSM6919154)		0,00003
S16-09088	KRAS	chr12:25398284	CC/TC	SNV	1	Hotspot	Gain-of-function	COSM521	21,7	28,0	p.Gly12Asp	129	2	NM_033360,3	c.35G>A	missense	0,517	0,98 (COSM521)	Pathogenic	0,00001
S16-09088	RB1	chr13:49039378	G/A	SNV	1			COSM4047930	6,9	15,0	p.Ser788Asn	218	23	NM_000321,2	c.2363G>A	missense	1	0,99(COSM4047930)		0,0002
S16-09088	SMAD4	chr18:48604809	C/T	SNV	1			COSM6985223	5,6	17,0	p.Pro544Leu	303	12	NM_005359,5	c.1631C>T	missense	0,154	0,99(COSM6985223)		0,0009
S17-00048	AKT3	chr1:243858916	G/A	SNV	1			COSM6982201	5,1	50,0	p.Pro50Leu	977	2	NM_005465,4	c.149C>T	missense	0,999	0,99(COSM6982201)		0,00001
S17-00048	APC	chr5:112177613	G/A	SNV	1			COSM6948248	5,9	68,0	p.Ala2108Thr	1144	16	NM_000038,5	c.6322G>A	missense	1	0,99(COSM6948248)	Uncertain significance	0,00001
S17-00048	APC	chr5:112175996	G/A	SNV	1			COSM5990842	7,7	64,0	p.Asp1569Asn	830	16	NM_000038,5	c.4705G>A	missense	0,978	0,98(COSM5990842)	Uncertain significance	0,00001
S17-00048	APC	chr5:112175639	C/T	SNV	1	Deleterious	Loss-of-Function	COSM13127	5,3	45,0	p.Arg1450Ter	856	16	NM_000038,5	c.4348C>T	nonsense		0,90(COSM13127)	Pathogenic	0,00016
S17-00048	APC	chr5:112175939	G/A	SNV	1			COSM5990838	5,3	45,0	p.Glu1550Lys	856	16	NM_000038,5	c.4648G>A	missense	0	0,86(COSM5990838)		0,00001
S17-00048	APC	chr5:112175945	G/T	SNV	1	Deleterious	Loss-of-Function	COSM41616	5,1	44,0	p.Glu1552Ter	859	16	NM_000038,5	c.4654G>T	nonsense		0,86(COSM41616)		0,00001
S17-00048	APC	chr5:112174658	C/T	SNV	1	Deleterious	Loss-of-Function	COSM1432255	5,1	21,0	p.Gln1123Ter	410	16	NM_000038,5	c.3367C>T	nonsense		0,83(COSM1432255)	Pathogenic	0,00092
S17-00048	ARID2	chr12:46240650	G/A	SNV	1			COSM5990677	8,5	74,0	p.Val504Ile	867	12	NM_152641,3	c.1510G>A	missense	0,231	0,97(COSM5990677)		0,00001
S17-00048	ATM	chr11:108155022	C/A	SNV	1			COSM6854120	5,2	103,1	p.Ala1272Asp	1998	26	NM_000051,3	c.3815C>A	missense	0,999	0,95(COSM6854120)		0,00001
S17-00048	ATM	chr11:108127029	G/A	SNV	1			COSM5024937	5,6	45,0	p.Glu738Lys	806	14	NM_000051,3	c.2212G>A	missense	0,045	0,97(COSM5024937)		0,00001
S17-00048	ATM	chr11:108196860	G/A	SNV	1			COSM8101523	5,2	34,0	p.Glu2295Lys	652	47	NM_000051,3	c.6883G>A	missense	1	0,99(COSM8101523)		0,00003
S17-00048	BAP1	chr3:52443589	G/A	SNV	1			COSM4991930	5,7	35,0	p.Leu35Phe, p.?	611	3	NM_004656,3	c.103C>T. c.-3307G>A	missense, unknown	1	0,97(COSM4991930)		0,00001
S17-00048	BAP1	chr3:52436848	C/T	SNV	1			COSM7335246	7,4	30,0	p.Ala644Thr	408	15	NM_004656,3	c.1930G>A	missense	0,916	0,95(COSM7335246)		0,00001
S17-00048	BAP1	chr3:52436341	C/T	SNV	1			COSM8548881	5,9	11,0	p.Arg718Gln	186	17	NM_004656,3	c.2153G>A	missense	0,105	0,98(COSM8548881)		0,00346
S17-00048	CDKN2A	chr9:21971066	G/A	SNV	1			COSM13787	9,5	19,0	p.His98Tyr	201	2	NM_001195132,1	c.292C>T	missense	0,999	0,88(COSM13787)	Uncertain significance	0,00001
S17-00048	EGFR	chr7:55249155	G/A	SNV	1			COSM23611	12,9	61,0	p.Cys818Tyr	472	20	NM_005228,4	c.2453G>A	missense	1	0,97(COSM23611)		0,00001
S17-00048	FGFR2	chr10:123243305	C/T	SNV	1			COSM1730961	5,2	35,0	p.Met736Ile	668	17	NM_000141,4	c.2208G>A	missense	1	0,97(COSM1730961)		0,00002

ID	Genes	Locus	Genotype	Type	Length	Oncomine Variant Class	Oncomine Gene Class	Variant ID	% Frequency	Mutated Alleles	Amino Acid Change	Coverage	Exon	Transcript	Coding	Variant Effect	PolyPhen	FATHMM	ClinVar	p-value
S17-00048	GNA11	chr19:3119306	G/A	SNV	1			COSM6944083	5,5	19,0	p.Glu280Lys	348	6	NM_002067,4	c.838G>A	missense	0,006	0,97(COSM6944083)		0,00071
S17-00048	KIT	chr4:55594177	C/T	SNV	1			COSM19323	5,2	30,0	p.Pro627Leu	583	13	NM_000222,2	c.1880C>T	missense	0,996	0,95(COSM19323)		0,00011
S17-00048	KRAS	chr12:25398284	CC/TC	SNV	1	Hotspot	Gain-of-Function	COSM521	6,7	37,0	p.Gly12Asp	552	2	NM_033360,3	c.35G>A	missense	0,517	0,98(COSM521)	Pathogenic	0,00001
S17-00048	MSH2	chr2:47635614	C/T	SNV	1			COSM3939104	6,4	65,0	p.Arg96Cys	1023	2	NM_000251,2	c.286C>T	missense	1	0,89(COSM3939104)	Uncertain significance	0,00001
S17-00048	MSH2	chr2:47707933	G/A	SNV	1			COSM6961836	5,2	34,0	p.Glu853Lys	651	15	NM_000251,2	c.2557G>A	missense	0,12	0,99(COSM6961836)		0,00003
S17-00048	MSH3	chr5:80109475	G/A	SNV	1			COSM6475019	5,6	16,0	p.Ala910Thr	284	20	NM_002439,4	c.2728G>A	missense	0,474	0,99(COSM6475019)		0,00112
S17-00048	NF1	chr17:29562654	C/T	SNV	1			COSM7344871	6,8	115,0	p.Thr1245Ile	1681	28	NM_001042492,2	c.3734C>T	missense	0,792	0,98(COSM7344871)		0,00001
S17-00048	NF1	chr17:29665096	G/A	SNV	1			COSM1189437	7,2	98,0	p.Gly2253Glu	1354	45	NM_001042492,2	c.6758G>A	missense	1	0,99(COSM1189437)	Uncertain significance	0,00001
S17-00048	NRAS	chr1:115256524	C/T	SNV	1			COSM4385830	5,9	12,0	p.Glu63Lys	205	3	NM_002524,4	c.187G>A	missense	0,989	0,99(COSM4385830)		0,00264
S17-00048	NRAS	chr1:115256551	C/A	SNV	1			COSM8294045	5,5	11,0	p.Asp54Tyr	201	3	NM_002524,4	c.160G>T	missense	1	0,99(COSM8294045)		0,006
S17-00048	PIK3CA	chr3:178937006	G/A	SNV	1			COSM5347016	6,8	56,0	p.Glu563Lys	830	11	NM_006218,3	c.1687G>A	missense	0,988	0,99(COSM5347016)		0,00001
S17-00048	PMS2	chr7:6042259	G/A	SNV	1			COSM9348046	31,6	37,0	p.Thr121Ile	117	5	NM_000535,6	c.362C>T	missense	0,93	0,88(COSM9348046)		0,00001
S17-00048	PMS2	chr7:6042221	G/A	SNV	1	Deleterious	Loss-of-Function	COSM7665273	20,0	23,0	p.Arg134Ter	115	5	NM_000535,6	c.400C>T	nonsense		0,97(COSM7665273)	Pathogenic	0,00001
S17-00048	POLD1	chr19:50912456	C/T	SNV	1			COSM7340145	7,4	53,0	p.Pro657Leu	713	16	NM_001256849,1	c.1970C>T	missense	0,999	0,98(COSM7340145)		0,00001
S17-00048	POLD1	chr19:50906751	C/T	SNV	1			COSM5028601	6,4	25,0	p.Ala380Val	388	10	NM_001256849,1	c.1139C>T	missense	0,915	0,93(COSM5028601)		0,00001
S17-00048	POLD1	chr19:50918998	C/T	SNV	1			COSM8895765	10,7	13,0	p.Pro912Leu	121	22	NM_001256849,1	c.2735C>T	missense	0,531	0,99(COSM8895765)	Uncertain significance	0,00001
S17-00048	POLD1	chr19:50919007	C/T	SNV	1			COSM6964406	9,1	11,0	p.Ala915Val	121	22	NM_001256849,1	c.2744C>T	missense	0,999	0,99(COSM6964406)	Uncertain significance	0,0001
S17-00048	POLD1	chr19:50918997	C/T	SNV	1			COSM40483	9,1	11,0	p.Pro912Ser	121	22	NM_001256849,1	c.2734C>T	missense	0,576	0,99(COSM40483)	Uncertain significance	0,0001
S17-00048	PTEN	chr10:89720822	C/T	SNV	1			COSM1349616	5,9	69,0	p.Leu325Phe	1167	8	NM_000314,6	c.973C>T	missense	0,971	0,72(COSM1349616)		0,00001
S17-00048	PTEN	chr10:89725081	C/T	SNV	1			COSM5945178	5,6	55,0	p.Ser355Leu	984	9	NM_000314,6	c.1064C>T	missense	0,041	0,98(COSM5945178)		0,00001
S17-00048	RAD51D	chr17:33428031	C/T	SNV	1			COSM4563742	7,6	34,0	p.Asp198Asn	445	7	NR_037714,1	c.592G>A	missense	0,988	0,90(COSM4563742)		0,00001
S17-00048	RB1	chr13:49039501	C/T	SNV	1			COSM6939091	7,0	25,0	p.Ser829Leu	358	23	NM_000321,2	c.2486C>T	missense	0,998	0,99(COSM6939091)		0,00001
S17-00048	SMAD4	chr18:48581307	C/T	SNV	1			COSM6438211	10,9	41,0	p.Ser204Phe	375	5	NM_005359,5	c.611C>T	missense	0,242	0,99(COSM6438211)		0,00001
S17-00048	SMAD4	chr18:48591808	G/A	SNV	1			COSM14189	6,5	30,0	p.Cys324Tyr	462	9	NM_005359,5	c.971G>A	missense	1	1,00(COSM1,0041,0089)		0,00001
S17-00048	SMAD4	chr18:48575065	C/T	SNV	1			COSM9992576	6,6	14,0	p.Arg87Trp	213	3	NM_005359,5	c.259C>T	missense	1	0,99(COSM9992576)		0,00046
S17-00048	SMAD4	chr18:48575095	C/T	SNV	1			COSM8273522	6,6	14,0	p.Arg97Cys	213	3	NM_005359,5	c.289C>T	missense	1	0,98(COSM8273522)	Uncertain significance	0,00046
S17-00048	TP53	chr17:7577574	T/C	SNV	1	Hotspot	Loss-of-Function	COSM10731	7,3	53,0	p.Tyr236Cys	724	7	NM_000546,5	c.707A>G	missense	0,999	0,90(COSM10731)	Likely pathogenic	0,00001
S17-00048	TP53	chr17:7577584	G/A	SNV	1			COSM44705	6,6	48,0	p.His233Tyr	724	7	NM_000546,5	c.697C>T	missense	0,804	0,96(COSM44705)		0,00001
S17-00048	TP53	chr17:7578274	TGA/TAA	SNV	1	Deleterious	Loss-of-Function	COSM10733	6,8	23,0	p.Gln192Ter	340	6	NM_000546,5	c.574C>T	nonsense		0,93(COSM10733)	Pathogenic	0,01636
S17-04055	ARID2	chr12:46230679	C/T	SNV	1			COSM4041985	5,6	32,0	p.Arg310Cys	567	8	NM_152641,3	c.928C>T	missense	1	0,98(COSM4041985)		0,00001
S17-04055	CDK4	chr12:58144521	C/T	SNV	1			COSM6914569	6,8	20,0	p.Glu184Lys	293	5	NM_000075,3	c.550G>A	missense	1	0,95(COSM6914569)	Uncertain significance	0,00003
S17-04055	KRAS	chr12:25398284	CC/AC	SNV	1	Hotspot	Gain-of-function	COSM520	15,0	27,0	p.Gly12Val	180	2	NM_033360,3	c.35G>T	missense	0,999	0,98(COSM520)	Pathogenic	0,00001
S17-04055	MLH1	chr3:37042536	C/T	SNV	1	Deleterious	Loss-of-function	COSM29727	9,5	29,0	p.Arg100Ter	304	3	NM_000249,3	c.298C>T	nonsense		0,88(COSM29727)	Pathogenic	0,00001
S17-04055	NF1	chr17:29562668	C/T	SNV	1			COSM436322	16,4	211,0	p.Arg1250Trp	1290	28	NM_001042492,2	c.3748C>T	missense	1	0,96(COSM436322)	Uncertain significance	0,00001
S17-04055	PTEN	chr10:89720727	G/T	SNV	1			COSM1349605	7,6	26,0	p.Gly293Val	341	8	NM_000314,6	c.878G>T	missense	1	1,00(COSM1,00349605)		0,00001
S17-04055	PTEN	chr10:89720768	G/T	SNV	1	Deleterious	Loss-of-function	COSM4718706	5,2	18,0	p.Glu307Ter	345	8	NM_000314,6	c.919G>T	nonsense		0,99(COSM4718706)	Pathogenic	0,00153
S17-04055	RB1	chr13:49054142	C/T	SNV	1	Deleterious	Loss-of-function	COSM7685017	5,8	50,0	p.Arg908Ter	867	27	NM_000321,2	c.2722C>T	nonsense		0,89(COSM7685017)		0,00001
S17-04055	SMAD4	chr18:48575194	C/T	SNV	1			COSM33139	7,1	20,0	p.Pro130Ser	281	3	NM_005359,5	c.388C>T	missense	1	0,98(COSM33139)	Uncertain significance	0,00001
S17-04055	TP53	chr17:7579358	CG/C	INDEL	1	Deleterious	Loss-of-function	COSM44669	22,7	443,0	p.Arg110ValfsTer13	1950	4	NM_000546,5	c.328delC	frameshiftDeletion		1,00(COSM44669)	Pathogenic	0,00001
S17-05387	APC	chr5:112173798	C/A	SNV	1	Deleterious	Loss-of-function	COSM4168332	5,4	18,0	p.Ser836Ter	334	16	NM_000038,5	c.2507C>A	nonsense		0,97(COSM4168332)		0,00105
S17-05387	KRAS	chr12:25398284	CC/TC	SNV	1	Hotspot	Gain-of-function	COSM521	35,9	35,9	p.Gly12Asp	100	2	NM_033360,3	c.35G>A	missense	0,517	0,98(COSM521)	Pathogenic	0,00001
S17-05387	PIK3CA	chr3:178948079	C/T	SNV	1			COSM5074888	9,9	24,0	p.Arg951Cys	243	20	NM_006218,3	c.2851C>T	missense	1	0,96(COSM5074888)		0,00001
S17-05387	PTEN	chr10:89720730	G/A	SNV	1			COSM1717478	11,7	12,0	p.Ser294Asn	103	8	NM_000314,6	c.881G>A	missense	0,175	0,97(COSM1717478)	Uncertain significance	0,00001
S17-05387	TP53	chr17:7577539	G/A	SNV	1	Hotspot	Loss-of-function	COSM10656	8,6	10,0	p.Arg248Trp	116	7	NM_000546,5	c.742C>T	missense	1	0,94(COSM10656)	Pathogenic	0,00117
S17-06560	EGFR	chr7:55259448	C/T	SNV	1	Hotspot	Gain-of-Function	COSM28604	8,8	14,0	p.Arg836Cys	159	21	NM_005228,4	c.2506C>T	missense	1	0,95(COSM28604)		0,00016
S17-06560	FGFR2	chr10:123279618	C/T	SNV	1			COSM6197915	6,9	21,0	p.Gly272Arg	303	7	NM_000141,4	c.814G>A	missense	0,007	0,90(COSM6197915)		0,00002
S17-06560	NF1	chr17:29562669	G/A	SNV	1			COSM6030784	8,8	14,0	p.Arg1250Gln	160	28	NM_001042492,2	c.3749G>A	missense	0,995	0,99(COSM6030784)	Uncertain significance	0,00002
S17-07604	KRAS	chr12:25398284	CC/TC	SNV	1	Hotspot	Gain-of-function	COSM521	49,0	116,0	p.Gly12Asp	237	2	NM_033360,3	c.35G>A	missense	0,517	0,98(COSM521)	Pathogenic	0,00001
S17-08807	AKT3	chr1:243777028	G/A	SNV	1			COSM4990545	6,7	41,0	p.Ser214Phe	613	7	NM_005465,4	c.641C>T	missense	1	0,98(COSM4990545)		0,00001
S17-08807	ALK	chr2:29432665	G/A	SNV	1			COSM442798	6,2	34,0	p.Arg1275Ter	545	25	NM_004304,4	c.3823C>T	nonsense		0,93(COSM442798)	Uncertain significance	0,00001
S17-08807	APC	chr5:112175303	C/T	SNV	1	Deleterious	Loss-of-Function	COSM13129	5,3	83,0	p.Gln1338Ter	1560	16	NM_000038,5	c.4012C>T	nonsense		0,90(COSM13129)	Pathogenic	0,00001
S17-08807	APC	chr5:112090719	G/A	SNV	1			COSM6475381	5,1	82,0	p.Met44Ile	1605	2	NM_000038,5	c.132G>A	missense	0,99	0,99(COSM6475381)		0,00001
S17-08807	APC	chr5:112173465	C/T	SNV	1			COSM1059565	5,4	77,0	p.Ala725Val	1436	16	NM_000038,5	c.2174C>T	missense	1	0,99(COSM1059565)		0,00001
S17-08807	APC	chr5:112175376	C/T	SNV	1			COSM30778	6,2	76,0	p.Ser1362Phe	1234	16	NM_000038,5	c.4085C>T	missense	0,769	0,94(COSM30778)		0,00001
S17-08807	APC	chr5:112151204	C/T	SNV	1	Deleterious	Loss-of-Function	COSM19679	15,4	68,0	p.Arg283Ter	441	9	NM_000038,5	c.847C>T	nonsense		0,98(COSM19679)	Pathogenic	0,00001
S17-08807	APC	chr5:112175876	C/T	SNV	1	Deleterious	Loss-of-Function	COSM1183187	10,3	54,0	p.Gln1529Ter	522	16	NM_000038,5	c.4585C>T	nonsense		0,87(COSM1183187)	Pathogenic	0,00001
S17-08807	APC	chr5:112137039	G/A	SNV	1			COSM9106966	11,1	53,0	p.Gly265Arg	477	8	NM_000038,5	c.793G>A	missense	0,704	0,97(COSM9106966)		0,00001
S17-08807	APC	chr5:112170811	G/A	SNV	1			COSM6475370	10,4	43,0	p.Gly636Asp	413	15	NM_000038,5	c.1907G>A	missense	1	0,99(COSM6475370)		0,00001
S17-08807	APC	chr5:112178211	C/T	SNV	1			COSM1183194	8,7	40,0	p.Ser2307Leu	462	16	NM_000038,5	c.6920C>T	missense	0,999	0,93(COSM1183194)	Uncertain significance	0,00001
S17-08807	APC	chr5:112173893	G/T	SNV	1	Deleterious	Loss-of-Function	COSM98383	9,6	36,0	p.Glu868Ter	374	16	NM_000038,5	c.2602G>T	nonsense		1(COSM98383)		0,00001
S17-08807	APC	chr5:112179632	G/A	SNV	1			COSM6475451	8,7	31,0	p.Val2781Met	356	16	NM_000038,5	c.8341G>A	missense	1	0,99(COSM6475451)		0,00001
S17-08807	ARID2	chr12:46245067	C/T	SNV	1			COSM5922947	7,2	80,0	p.Ser1054Phe	1114	15	NM_152641,3	c.3161C>T	missense	0,911	0,96(COSM5922947)		0,00001
S17-08807	ARID2	chr12:46230549	G/A	SNV	1	Deleterious	Loss-of-Function	COSM4990225	5,8	74,0	p.Trp266Ter	1280	8	NM_152641,3	c.798G>A	nonsense		0,98(COSM4990225)		0,00001
S17-08807	ARID2	chr12:46230676	A/G	SNV	1			COSM266035	7,6	72,0	p.Asn309Asp	945	8	NM_152641,3	c.925A>G	missense	0,997	0,97(COSM266035)		0,00001
S17-08807	ARID2	chr12:46244931	G/A	SNV	1			COSM1245457	7,7	70,0	p.Val1009Met	908	15	NM_152641,3	c.3025G>A	missense	0,493	0,93(COSM1245457)		0,00001
S17-08807	ARID2	chr12:46205322	C/T	SNV	1			COSM6948449	6,6	56,0	p.His136Tyr	854	4	NM_152641,3	c.406C>T	missense	0,92	0,99(COSM6948449)		0,00001
S17-08807	ARID2	chr12:46205332	C/A	SNV	1	Deleterious	Loss-of-Function	COSM6974469	5,4	46,0	p.Ser139Ter	855	4	NM_152641,3	c.416C>A	nonsense		0,99(COSM6974469)		0,00001
S17-08807	ARID2	chr12:46243442	G/A	SNV	1			COSM4663268	10,9	38,0	p.Val599Ile	350	14	NM_152641,3	c.1795G>A	missense	0,894	0,95(COSM4663268)		0,00001
S17-08807	ARID2	chr12:46243487	C/T	SNV	1	Deleterious	Loss-of-Function	COSM6972311	6,6	36,0	p.Gln614Ter	546	14	NM_152641,3	c.1840C>T	nonsense		0,99(COSM6972311)		0,00001
S17-08807	ATM	chr11:108214004	C/T	SNV	1			COSM5413308	7,2	144,0	p.Pro2775Leu	2000	57	NM_000051,3	c.8324C>T	missense	1	0,99(COSM5413308)		0,00001
S17-08807	BAP1	chr3:52440858	G/A	SNV	1			COSM5527250	16,1	77,0	p.Leu216Phe	479	8	NM_004656,3	c.646C>T	missense	0,997	0,99(COSM5527250)		0,00001
S17-08807	BRIP1	chr17:59770827	C/T	SNV	1			COSM6855320	6,7	79,0	p.Asp847Asn	1181	18	NM_032043,2	c.2539G>A	missense	0,045	0,96(COSM6855320)	Uncertain significance	0,00001
S17-08807	CHEK2	chr22:29121084	G/A	SNV	1			COSM6432552	5,2	104,0	p.Ala158Val	2000	4	NM_007194,4	c.473C>T	missense	0,001	0,93(COSM6432552)	Uncertain significance	0,00001
S17-08807	EGFR	chr7:55221843	C/T	SNV	1			COSM9446911	7,0	32,0	p.Pro296Leu	457	7	NM_005228,4	c.887C>T	missense	1	0,99(COSM9446911)		0,00001
S17-08807	GNAQ	chr9:80336364	C/T	SNV	1			COSM4834569	9,3	37,0	p.Asp319Asn	397	7	NM_002072,4	c.955G>A	missense	0,004	0,98(COSM4834569)		0,00001
S17-08807	KIT	chr4:55593644	ACA/ATA	SNV	1			COSM1287	9,3	114,0	p.Thr574Ile	1223	11	NM_000222,2	c.1721C>T	missense	0,971	0,93(COSM1287)	Uncertain significance	0,00001
S17-08807	KIT	chr4:55602694	G/A	SNV	1			COSM1324	5,1	47,0	p.Glu839Lys	927	18	NM_000222,2	c.2515G>A	missense	1	0,99(COSM1324)	Pathogenic/Likely pathogenic	0,00001
S17-08807	KRAS	chr12:25398284	CC/AC	SNV	1	Hotspot	Gain-of-Function	COSM520	5,2	20,0	p.Gly12Val	387	2	NM_033360,3	c.35G>T	missense	0,999	0,98(COSM520)	Pathogenic	0,00803

ID	Genes	Locus	Genotype	Type	Length	Oncomine Variant Class	Oncomine Gene Class	Variant ID	% Frequency	Mutated Alleles	Amino Acid Change	Coverage	Exon	Transcript	Coding	Variant Effect	PolyPhen	FATHMM	ClinVar	p-value
S17-08807	MLH1	chr3:37089095	G/A	SNV	1			COSM5685374	5,4	78,1	p.Gly606Glu	1443	16	NM_000249,3	c.1817G>A	missense	0,004	0,94(COSM5685374)		0,00001
S17-08807	MLH1	chr3:37061853	G/T	SNV	1	Deleterious	Loss-of-Function	COSM730237	5,9	14,0	p.Glu313Ter	238	11	NM_000249,3	c.937G>T	nonsense		1(COSM730237)		0,00131
S17-08807	MSH2	chr2:47657029	C/T	SNV	1	Deleterious	Loss-of-Function	COSM7508782	9,5	124,1	p.Gln409Ter	1310	7	NM_000251,2	c.1225C>T	nonsense		0,93(COSM7508782)	Pathogenic	0,00001
S17-08807	MSH6	chr2:48025901	G/T	SNV	1			COSM6542559	5,8	43,0	p.Gly260Val	738	4	NM_000179,2	c.779G>T	missense	0,999	0,94(COSM6542559)		0,00001
S17-08807	MSH6	chr2:48018188	G/A	SNV	1			COSM4094430	5,3	22,0	p.Arg128His	418	2	NM_000179,2	c.383G>A	missense	0,606	0,94(COSM4094430)	Uncertain significance	0,00052
S17-08807	NF1	chr17:29560088	C/T	SNV	1	Deleterious	Loss-of-Function	COSM6963110	7,3	117,0	p.Gln1189Ter	1607	27	NM_001042492,2	c.3565C>T	nonsense		0,99(COSM6963110)	Pathogenic	0,00001
S17-08807	NF1	chr17:29557384	C/T	SNV	1	Deleterious	Loss-of-Function	COSM5049807	13,9	58,0	p.Gln1033Ter	417	23	NM_001042492,2	c.3097C>T	nonsense		0,99(COSM5049807)	Pathogenic	0,00001
S17-08807	NRAS	chr1:115252345	G/A	SNV	1			COSM6903912	8,7	46,0	p.Gln99Ter	530	4	NM_002524,4	c.295C>T	nonsense		0,98(COSM6903912)		0,00001
S17-08807	PIK3CA	chr3:178917681	G/A	SNV	1			COSM4991881	15,6	88,0	p.Asp186Asn	566	3	NM_006218,3	c.556G>A	missense	0,921	0,91(COSM4991881)		0,00001
S17-08807	PIK3CA	chr3:178952100	C/T	SNV	1			COSM33601	5,2	64,0	p.Thr1052Ile	1221	21	NM_006218,3	c.3155C>T	missense	0,972	0,95(COSM33601)		0,00001
S17-08807	POLD1	chr19:50912919	C/T	SNV	1			COSM6955592	9,8	20,0	p.Ser717Leu	204	17	NM_001256849,1	c.2150C>T	missense	0,998	0,91(COSM6955592)		0,00001
S17-08807	POLE	chr12:133219848	G/A	SNV	1			COSM9678406	11,5	67,0	p.Pro1505Ser	581	35	NM_006231,3	c.4513C>T	missense	0,129	1(COSM9678406)		0,00001
S17-08807	POLE	chr12:133210787	G/A	SNV	1			COSM9798411	6,7	42,0	p.Leu1997Phe	629	43	NM_006231,3	c.5989C>T	missense	0,964	0,96(COSM9798411)		0,00001
S17-08807	POLE	chr12:133220098	CCA/C	INDEL	2	Deleterious	Loss-of-Function	COSM1745059	7,1	25,0	p.Val1446GlyfsTer3	354	34	NM_006231,3	c.4337_4338delTG	frameshiftDeletion		1,00(COSM1745059)	Uncertain significance	0,00004
S17-08807	POLE	chr12:133219913	C/T	SNV	1			COSM5840587	6,5	16,0	p.Ser1483Asn	245	35	NM_006231,3	c.4448G>A	missense	0,012	0,98(COSM5840587)		0,00023
S17-08807	POLE	chr12:133254205	G/A	SNV	1			COSM9806552	6,4	13,0	p.Pro227Ser	202	7	NM_006231,3	c.679C>T	missense	0,998	1(COSM9806552)		0,00082
S17-08807	PTEN	chr10:89711888	C/T	SNV	1			COSM4990009	12,4	73,0	p.Pro169Leu	588	6	NM_000314,6	c.506C>T	missense	1	0,95(COSM4990009)		0,00001
S17-08807	PTEN	chr10:89692995	C/T	SNV	1			COSM14245	5,7	27,0	p.Thr160Ile	476	5	NM_000314,6	c.479C>T	missense	1	0,98(COSM14245)	Likely pathogenic	0,00004
S17-08807	PTEN	chr10:89692805	C/T	SNV	1	Deleterious	Loss-of-Function	COSM6868591	5,7	24,0	p.Gln97Ter	423	5	NM_000314,6	c.289C>T	nonsense		0,98(COSM6868591)	Pathogenic	0,00011
S17-08807	PTEN	chr10:89653800	T/C	SNV	1			COSM4408405	6,0	23,0	p.Ile33Thr	381	2	NM_000314,6	c.98T>C	missense	0,997	0,97(COSM4408405)	Uncertain significance	0,00006
S17-08807	RAD51C	chr17:56774132	G/T	SNV	1			COSM6934992	5,9	25,0	p.Glu161Asp	422	3	NM_058216,2	c.483G>T	missense	0,995	0,75(COSM6934992)		0,00004
S17-08807	RB1	chr13:49030410	G/A	SNV	1			COSM6966618	9,8	32,0	p.Glu629Lys	326	19	NM_000321,2	c.1885G>A	missense	0,146	0,97(COSM6966618)		0,00001
S17-08807	SMAD4	chr18:48604709	C/T	SNV	1			COSM9233224	5,2	92,9	p.Pro511Ser	1801	12	NM_005359,5	c.1531C>T	missense	0,005	0,98(COSM9233224)		0,00001
S17-08807	SMAD4	chr18:48575225	G/A	SNV	1			COSM5610595	10,5	58,0	p.Gly140Glu	554	3	NM_005359,5	c.419G>A	missense	0,998	1,00(COSM561,000595)		0,00001
S17-08807	TP53	chr17:7578508	C/T	SNV	1	Hotspot	Loss-of-Function	COSM43708	11,5	81,0	p.Cys141Tyr	706	5	NM_000546,5	c.422G>A	missense	1	0,99(COSM43708)	Pathogenic/Likely pathogenic	0,00001
S17-08807	TP53	chr17:7577093	C/T	SNV	1	Hotspot	Loss-of-Function	COSM44338	5,8	77,1	p.Arg282Gln	1340	8	NM_000546,5	c.845G>A	missense	1	0,98(COSM44338)	Conflicting interpretations of pat	0,00001
S17-08807	TP53	chr17:7577105	G/A	SNV	1	Hotspot	Loss-of-Function	COSM10863	5,8	77,0	p.Pro278Leu	1337	8	NM_000546,5	c.833C>T	missense	1	1,00(COSM1,000863)	Pathogenic	0,00001
S17-08807	TP53	chr17:7577139	G/A	SNV	1			COSM11183	12,9	76,0	p.Arg267Trp	588	8	NM_000546,5	c.799C>T	missense	0,979	0,98(COSM11183)	Conflicting interpretations of pat	0,00001
S17-08807	TP53	chr17:7578212	G/A	SNV	1	Deleterious	Loss-of-Function	COSM10654	13,8	60,0	p.Arg213Ter	436	6	NM_000546,5	c.637C>T	nonsense		0,95(COSM10654)	Pathogenic	0,00001
S17-08807	TP53	chr17:7577599	C/A	SNV	1			COSM45786	10,9	50,0	p.Asp228Tyr	459	7	NM_000546,5	c.682G>T	missense	0,912	1,00(COSM45786)		0,00001
S17-08807	VHL	chr3:10191497	C/T	SNV	1	Deleterious	Loss-of-Function	COSM14397	5,3	52,0	p.Gln164Ter	975	3	NM_000551,3	c.490C>T	nonsense		0,90(COSM14397)	Pathogenic	0,00001
S18-00115	MYC	chr8:128750803	C/G	SNV	1			COSM6209969	6,1	13,0	p.Leu114Val	213	2	NM_002467,5	c.340C>G	missense	0,855	0,96(COSM6209969)		0,00134
S18-00728	APC	chr5:112174888	G/T	SNV	1			COSM6602058	5,3	48,0	p.Lys1199Asn	901	16	NM_000038,5	c.3597G>T	missense	0,006	0,91(COSM6602058)		0,00001
S18-00728	KRAS	chr12:25398284	CC/AC	SNV	1	Hotspot	Gain-of-function	COSM520	8,0	40,0	p.Gly12Val	498	2	NM_033360,3	c.35G>T	missense	0,999	0,98(COSM520)	Pathogenic	0,00001
S18-00728	SMAD4	chr18:48604747	C/A	SNV	1	Deleterious	Loss-of-function	COSM9101276	5,7	48,0	p.Cys523Ter	846	12	NM_005359,5	c.1569C>A	nonsense		0,94(COSM9101276)		0,00001
S18-00728	TP53	chr17:7574003	G/A	SNV	1	Deleterious	Loss-of-function	COSM11073	6,2	124,0	p.Arg342Ter	2000	10	NM_000546,5	c.1024C>T	nonsense		0,73(COSM11073)	Pathogenic	0,00001
S18-01550	MYC	chr8:128750803	C/G	SNV	1			COSM6209969	7,2	104,0	p.Leu114Val	1438	2	NM_002467,5	c.340C>G	missense	0,855	0,96(COSM6209969)		0,00001
S18-02626	CDKN2A	chr9:21970971	G/T	SNV	1	Deleterious	Loss-of-function	COSM28562	46,6	234,0	p.Tyr129Ter	502	2	NM_001195132,1	c.387C>A	nonsense		0,89(COSM28562)		0,00001
S18-02626	KRAS	chr12:25398284	CC/AC	SNV	1	Hotspot	Gain-of-function	COSM520	46,1	583,0	p.Gly12Val	1264	2	NM_033360,3	c.35G>T	missense	0,999	0,98(COSM520)	Pathogenic	0,00001
S18-02626	MYC	chr8:128750803	C/G	SNV	1			COSM6209969	6,2	81,0	p.Leu114Val	1303	2	NM_002467,5	c.340C>G	missense	0,855	0,96(COSM6209969)		0,00001
S18-02626	TP53	chr17:7577539	G/A	SNV	1	Hotspot	Loss-of-function	COSM10656	54,0	1080,0	p.Arg248Trp	2000	7	NM_000546,5	c.742C>T	missense	1	0,94(COSM10656)	Pathogenic	0,00001
S18-02712	ALK	chr2:29436869	C/T	SNV	1			COSM1690369	5,8	26,0	p.Glu1242Lys	449	24	NM_004304,4	c.3724G>A	missense	0,999	0,99(COSM1690369)		0,00004
S18-02712	APC	chr5:112162891	C/T	SNV	1	Deleterious	Loss-of-function	COSM29364	12,8	41,0	p.Arg499Ter	320	12	NM_000038,5	c.1495C>T	nonsense		0,91(COSM29364)	Pathogenic	0,00001
S18-02712	APC	chr5:112173949	G/T	SNV	1			COSM1432214	6,2	23,0	p.Gln886His	372	16	NM_000038,5	c.2658G>T	missense	0,997	0,97(COSM1432214)	Conflicting interpretations of pat	0,00004
S18-02712	APC	chr5:112173648	G/A	SNV	1			COSM8516283	9,3	21,0	p.Arg786His	227	16	NM_000038,5	c.2357G>A	missense	0,998	0,99 (COSM8516283)	Uncertain significance	0,00001
S18-02712	ARID2	chr12:46230604	C/T	SNV	1			COSM189021	9,0	40,0	p.Arg285Trp	443	8	NM_152641,3	c.853C>T	missense	1	0,98(COSM189021)		0,00001
S18-02712	ARID2	chr12:46287446	C/T	SNV	1	Deleterious	Loss-of-function	COSM1628616	11,0	26,0	p.Arg1769Ter	236	20	NM_152641,3	c.5305C>T	nonsense		0,87(COSM1628616)	Pathogenic	0,00001
S18-02712	ATM	chr11:108188115	G/A	SNV	1			COSM6916987	8,6	27,0	p.Gly2072Arg	314	43	NM_000051,3	c.6214G>A	missense	1	0,98(COSM6916987)	Uncertain significance	0,00001
S18-02712	ATM	chr11:108236086	C/T	SNV	1	Hotspot	Loss-of-function	COSM21642	5,8	23,0	p.Arg3008Cys	395	63	NM_000051,3	c.9022C>T	missense	1	0,98(COSM21642)	Pathogenic/Likely pathogenic	0,00118
S18-02712	ATM	chr11:108137973	G/T	SNV	1	Deleterious	Loss-of-function	COSM6201645	7,3	19,0	p.Glu848Ter	259	17	NM_000051,3	c.2542G>T	nonsense		0,95(COSM6201645)	Likely pathogenic	0,00002
S18-02712	BAP1	chr3:52436392	C/T	SNV	1			COSM9231255	10,2	24,0	p.Arg701His	236	17	NM_004656,3	c.2102G>A	missense	0,995	0,96 (COSM9231255)		0,00001
S18-02712	BRAF	chr7:140482874	C/T	SNV	1			COSM4188569	8,1	24,0	p.Gly421Arg	298	10	NM_004333,5	c.1261G>A	missense	0,086	0,94(COSM4188569)		0,00001
S18-02712	CDH1	chr16:68863587	C/A	SNV	1			COSM8202963	5,4	17,0	p.Leu776Met	317	15	NM_004360,4	c.2326C>A	missense	1	0,98 (COSM8202963)		0,00146
S18-02712	CDKN2A	chr9:21971166	C/CTGCT	INDEL	4	Deleterious	Loss-of-function	(COSM6985610	19,4	39,0	p.Leu65AlafsTer56	201	2	NM_001195132,1	c.191_192insAGCA	frameshiftInsertion		1,00(COSM6985610)		0,00001
S18-02712	EGFR	chr7:55211097	G/A	SNV	1			COSM174732	49,5	51,0	p.Glu114Lys	103	3	NM_005228,4	c.340G>A	missense	0,019	0,98(COSM174732)		0,00001
S18-02712	EGFR	chr7:55227854	G/A	SNV	1			COSM1090874	13,0	41,0	p.Val441Ile	316	12	NM_005228,4	c.1321G>A	missense	0,072	0,92(COSM1090874)		0,00001
S18-02712	EGFR	chr7:55242451	C/T	SNV	1			COSM6196862	5,3	31,0	p.Pro741Ser	582	19	NM_005228,4	c.2221C>T	missense	1	0,99(COSM6196862)		0,00005
S18-02712	FANCD2	chr3:10138146	G/A	SNV	1			COSM6880911	23,2	29,0	p.Arg1392Gln	125	42	NM_033084,4	c.4175G>A.	missense, unknown	1	0,99(COSM6880911)		0,00001
S18-02712	FGFR2	chr10:123298199	C/T	SNV	1			COSM29832	5,3	24,0	p.Glu219Lys	456	6	NM_000141,4	c.655G>A	missense	0,932	0,99(COSM29832)		0,00032
S18-02712	GNAQ	chr9:80336333	G/A	SNV	1			COSM3218443	13,6	36,0	p.Thr329Met	265	7	NM_002072,4	c.986C>T	missense	1	0,99 (COSM3218443)		0,00001
S18-02712	GNAQ	chr9:80343561	G/A	SNV	1			COSM4605880	7,9	14,0	p.Ala253Val	178	6	NM_002072,4	c.758C>T	missense	0,997	0,99(COSM4605880)		0,00008
S18-02712	MSH2	chr2:47637430	G/T	SNV	1			COSM7795183	11,9	54,0	p.Glu188Asp	454	3	NM_000251,2	c.564G>T	missense	1	0,94 (COSM7795183)		0,00001
S18-02712	MSH2	chr2:47690259	G/T	SNV	1			COSM1668896	6,0	23,0	p.Met492Ile	383	9	NM_000251,2	c.1476G>T	missense	0,019	0,99(COSM1668896)		0,00006
S18-02712	MSH6	chr2:48026881	G/A	SNV	1			COSM6475150	11,1	43,0	p.Ala587Thr	386	4	NM_000179,2	c.1759G>A	missense	0,972	0,99(COSM6475150)	Uncertain significance	0,00001
S18-02712	MYC	chr8:128753188	G/A	SNV	1			COSM6915925	7,8	80,0	p.Arg450Gln	1022	3	NM_002467,5	c.1349G>A	missense	0,362	0,87(COSM6915925)		0,00001
S18-02712	NF1	chr17:29684077	C/T	SNV	1			COSM5484087	26,5	36,0	p.Pro2613Leu	136	53	NM_001042492,2	c.7838C>T	missense	0,015	0,98(COSM5484087)	Uncertain significance	0,00001
S18-02712	NF1	chr17:29661899	G/C	SNV	1			COSM6968058	6,6	35,0	p.Trp1952Cys	530	40	NM_001042492,2	c.5856G>C	missense	1	0,99(COSM6968058)		0,00001
S18-02712	NF1	chr17:29533378	C/T	SNV	1	Deleterious	Loss-of-function	COSM24464	14,1	33,0	p.Arg461Ter	234	12	NM_001042492,2	c.1381C>T	nonsense		0,99(COSM24464)	Pathogenic	0,00001
S18-02712	NRAS	chr1:115251226	C/T	SNV	1			COSM6729412	6,4	108,0	p.Arg167Gln	1695	5	NM_002524,4	c.500G>A	missense	0,012	0,91(COSM6729412)		0,00001
S18-02712	PIK3CA	chr3:178938835	C/T	SNV	1			COSM6917753	7,8	38,0	p.Arg693Cys	488	14	NM_006218,3	c.2077C>T	missense	1	0,92(COSM6917753)	Uncertain significance	0,00001
S18-02712	PIK3CA	chr3:178916648	G/T	SNV	1			COSM4998503	21,1	36,0	p.Gly12Val	171	2	NM_006218,3	c.35G>T	missense	1	1,00(COSM4998503)		0,00001
S18-02712	POLD1	chr19:50910258	C/T	SNV	1			COSM1681235	10,9	55,0	p.Arg505Cys	503	13	NM_001256849,1	c.1513C>T	missense	0,999	0,99(COSM1681235)	Uncertain significance	0,00001
S18-02712	POLE	chr12:133254004	C/T	SNV	1			COSM6920439	8,9	72,0	p.Arg249Gln	809	8	NM_006231,3	c.746G>A	missense	0,806	0,99(COSM6920439)	Uncertain significance	0,00001
S18-02712	POLE	chr12:133252337	C/T	SNV	1			COSM6752010	17,7	39,0	p.Gly364Arg	220	11	NM_006231,3	c.1090G>A	missense	1	0,99(COSM6752010)		0,00001
S18-02712	POLE	chr12:133214706	G/A	SNV	1			COSM6934810	11,9	36,0	p.Arg1858Cys	303	41	NM_006231,3	c.5572C>T	missense	1	0,99(COSM6934810)	Uncertain significance	0,00001
S18-02712	POLE	chr12:133237689	G/A	SNV	1			COSM6752020	7,3	13,0	p.Arg976Cys	177	25	NM_006231,3	c.2926C>T	missense	1	0,98(COSM6752020)	Uncertain significance	0,00025
S18-02712	PTEN	chr10:89720772	G/A	SNV	1			COSM1561195	11,8	63,0	p.Arg308His	532	8	NM_000314,6	c.923G>A	missense	0,007	0,98(COSM1561195)	Uncertain significance	0,00001
S18-02712	RB1	chr13:48937001	C/T	SNV	1	Deleterious	Loss-of-function	COSM126899	10,1	57,0	p.Gln257Ter	567	8	NM_000321,2	c.769C>T	nonsense		0,93(COSM126899)		0,00001

ID	Genes	Locus	Genotype	Type	Length	Oncomine Variant Class	Oncomine Gene Class	Variant ID	% Frequency	Mutated Alleles	Amino Acid Change	Coverage	Exon	Transcript	Coding	Variant Effect	PolyPhen	FATHMM	ClinVar	p-value
S18-02712	RB1	chr13:48936995	C/T	SNV	1	Deleterious	Loss-of-function	COSM943	10,1	57,0	p.Arg255Ter	567	8	NM_000321,2	c.763C>T	nonsense		0,76(COSM943)	Pathogenic	0,00001
S18-02712	SMAD4	chr18:48604664	C/T	SNV	1	Hotspot	Loss-of-function	COSM6476007	12,5	33,0	p.Arg496Cys	265	12	NM_005359,5	c.1486C>T	missense	1	0,98(COSM6476007)	Pathogenic/Likely pathogenic	0,00001
S18-02712	TP53	chr17:7577139	G/A	SNV	1			COSM11183	7,6	37,0	p.Arg267Trp	490	8	NM_000546,5	c.799C>T	missense	0,979	0,98(COSM11183)	Conflicting interpretations of pat	0,00001
S18-03289	SMAD4	chr18:48603032	C/T	SNV	1	Deleterious	Loss-of-function	COSM14096	6,2	59,0	p.Arg445Ter	960	11	NM_005359,5	c.1333C>T	nonsense		0,94(COSM14096)	Pathogenic	0,00001
S18-04801	ALK	chr2:29419650	C/T	SNV	1			COSM4820914	8,3	16,0	p.Glu1384Lys	193	28	NM_004304,4	c.4150G>A	missense	0,001	0,83(COSM4820914)		0,00002
S18-04801	APC	chr5:112173824	C/T	SNV	1			COSM9801623	11,7	59,0	p.Arg845Cys	504	16	NM_000038,5	c.2533C>T	missense	0,998	0,96(COSM9801623)	Uncertain significance	0,00001
S18-04801	APC	chr5:112177653	C/T	SNV	1			COSM6940328	14,3	50,0	p.Ala2121Val	351	16	NM_000038,5	c.6362C>T	missense	0,997	0,97(COSM6940328)		0,00001
S18-04801	APC	chr5:112174466	G/A	SNV	1			COSM9311935	5,2	30,0	p.Glu1059Lys	581	16	NM_000038,5	c.3175G>A	missense	0,996	1(COSM9311935)		0,00012
S18-04801	APC	chr5:112174664	G/A	SNV	1			COSM6019181	6,6	17,0	p.Val1125Ile	258	16	NM_000038,5	c.3373G>A	missense	0,004	0,89(COSM6019181)		0,00014
S18-04801	APC	chr5:112174713	C/T	SNV	1			COSM8306905	6,6	17,0	p.Thr1141Ile	256	16	NM_000038,5	c.3422C>T	missense	0,999	0,94(COSM8306905)		0,00013
S18-04801	ARID2	chr12:46230679	C/T	SNV	1			COSM4041985	5,5	28,0	p.Arg310Cys	514	8	NM_152641,3	c.928C>T	missense	1	0,98(COSM4041985)		0,00007
S18-04801	ARID2	chr12:46246270	G/A	SNV	1			COSM7697639	10,4	26,0	p.Ser1455Asn	251	15	NM_152641,3	c.4364G>A	missense	0	0,97(COSM7697639)		0,00001
S18-04801	ARID2	chr12:46298829	G/A	SNV	1			COSM4990228	6,0	18,0	p.Glu1826Lys	298	21	NM_152641,3	c.5476G>A	missense	0,039	0,90(COSM4990228)		0,00028
S18-04801	ARID2	chr12:46243427	G/A	SNV	1			COSM4663268	10,7	16,0	p.Val599Ile	149	14	NM_152641,3	c.1795G>A	missense	0,894	0,95(COSM4663268)		0,00001
S18-04801	ATM	chr11:108115744	C/T	SNV	1	Deleterious	Loss-of-Function	COSM140670	6,0	52,0	p.Gln298Ter	870	7	NM_000051,3	c.892C>T	nonsense		0,92(COSM140670)		0,00001
S18-04801	ATM	chr11:108218052	G/T	SNV	1			COSM6925192	7,2	41,0	p.Leu2877Phe	572	59	NM_000051,3	c.8631G>T	missense	1	0,89(COSM6925192)		0,00001
S18-04801	ATM	chr11:108224510	G/A	SNV	1			COSM686476	9,6	40,0	p.Gly2897Ser	415	60	NM_000051,3	c.8689G>A	missense	1	0,99(COSM686476)		0,00001
S18-04801	ATM	chr11:108199889	G/A	SNV	1			COSM6928530	15,7	39,0	p.Glu2411Lys	248	49	NM_000051,3	c.7231G>A	missense	1	0,99(COSM6928530)		0,00001
S18-04801	ATM	chr11:108218092	G/A	SNV	1			COSM6921577	6,8	39,0	p.Gly2891Ser	572	59	NM_000051,3	c.8671G>A	missense	1	0,99(COSM6921577)		0,00001
S18-04801	ATM	chr11:108099912	C/T	SNV	1	Deleterious	Loss-of-Function	COSM8747548	8,0	29,0	p.Gln65Ter	361	4	NM_000051,3	c.193C>T	nonsense		0,97(COSM8747548)	Likely pathogenic	0,00001
S18-04801	BAP1	chr3:52440379	C/T	SNV	1			COSM159326	8,3	31,0	p.Asp225Asn	372	9	NM_004656,3	c.673G>A	missense	0,995	0,99(COSM159326)		0,00001
S18-04801	BAP1	chr3:52441217	C/T	SNV	1			COSM4410937	6,0	21,0	p.Gly185Arg	352	7	NM_004656,3	c.553G>A	missense	1	0,99(COSM4410937)		0,00013
S18-04801	BAP1	chr3:52439179	G/A	SNV	1	Deleterious	Loss-of-Function	COSM5399743	6,1	18,0	p.Gln355Ter	296	11	NM_004656,3	c.1063C>T	nonsense		1,00(COSM5399743)	Pathogenic	0,00026
S18-04801	BRAF	chr7:140453128	G/A	SNV	1			COSM33729	10,1	32,0	p.Arg603Ter	316	15	NM_004333,5	c.1807C>T	nonsense		0,98(COSM33729)	not provided	0,00001
S18-04801	BRIP1	chr17:59926534	G/A	SNV	1	Deleterious	Loss-of-Function	COSM6465050	7,3	24,0	p.Gln155Ter	327	5	NM_032043,2	c.463C>T	nonsense		0,86(COSM6465050)	Pathogenic	0,00001
S18-04801	CDH1	chr16:68847304	G/A	SNV	1	Deleterious	Loss-of-Function	COSM3818351	8,2	28,0	p.Trp409Ter	342	9	NM_004360,4	c.1226G>A	nonsense		0,99(COSM3818351)		0,00001
S18-04801	CDKN2A	chr9:21974760	C/T	SNV	1			COSM5628983	6,2	11,0	p.Gly23Ser	179	1	NM_001195132,1	c.67G>A	missense	0,998	0,93(COSM5628983)	Likely pathogenic	0,00256
S18-04801	CHEK2	chr22:29130561	C/T	SNV	1			COSM6938075	7,2	33,0	p.Ser50Asn	458	2	NM_007194,4	c.149G>A	missense	0,993	0,92(COSM6938075)		0,00001
S18-04801	CHEK2	chr22:29130564	G/A	SNV	1			COSM1616324	5,2	24,0	p.Ser49Phe	458	2	NM_007194,4	c.146C>T	missense	0,362	0,78(COSM1616324)	Uncertain significance	0,00034
S18-04801	FANCD2	chr3:10116274	C/T	SNV	1	Deleterious	Loss-of-Function	COSM4484163	5,2	27,0	p.Arg926Ter	517	29	NM_033084,4	c.2776C>T	nonsense		0,98(COSM4484163)		0,00017
S18-04801	KIT	chr4:55603353	G/A	SNV	1			COSM4844805	10,4	24,0	p.Met903Ile	231	20	NM_000222,2	c.2709G>A	missense	0,715	0,99(COSM4844805)		0,00001
S18-04801	KRAS	chr12:25398290	C/T	SNV	1			COSM110699	6,2	15,0	p.Gly10Glu	241	2	NM_033360,3	c.29G>A	missense	1	0,98(COSM110699)		0,00055
S18-04801	MLH1	chr3:37067477	G/A	SNV	1			COSM6964602	27,5	30,0	p.Gly463Glu	109	12	NM_000249,3	c.1388G>A	missense	0,032	0,74(COSM6964602)		0,00001
S18-04801	MSH2	chr2:47705646	C/T	SNV	1	Deleterious	Loss-of-Function	COSM461018	6,1	23,0	p.Gln816Ter	376	14	NM_000251,2	c.2446C>T	nonsense		0,98(COSM461018)	Pathogenic	0,00005
S18-04801	MYC	chr8:128750803	C/G	SNV	1			COSM6209969	7,7	31,0	p.Leu114Val	405	2	NM_002467,5	c.340C>G	missense	0,855	0,96(COSM6209969)		0,00001
S18-04801	NF1	chr17:29662039	G/A	SNV	1			COSM9798119	5,3	28,0	p.Ser1999Asn	526	40	NM_001042492,2	c.5996G>A	missense	0,025	0,99(COSM9798119)		0,0001
S18-04801	POLD1	chr19:50912089	C/T	SNV	1			COSM6751889	9,6	29,0	p.Pro608Leu	301	15	NM_001256849,1	c.1823C>T	missense	1	0,95(COSM6751889)		0,00001
S18-04801	POLE	chr12:133218828	C/T	SNV	1			COSM6985195	8,6	24,0	p.Cys1703Tyr	280	38	NM_006231,3	c.5108G>A	missense	0,037	0,99(COSM6985195)		0,00001
S18-04801	PTEN	chr10:89720711	G/A	SNV	1			COSM5055	12,9	59,0	p.Glu288Lys	457	8	NM_000314,6	c.862G>A	missense	0,081	1,00(COSM5055)	Uncertain significance	0,00001
S18-04801	PTEN	chr10:89692841	G/A	SNV	1			COSM2155196	9,2	33,0	p.Asp109Asn	360	5	NM_000314,6	c.325G>A	missense	1	0,99(COSM2155196)		0,00001
S18-04801	RAD51C	chr17:56811555	G/A	SNV	1			COSM981989	6,0	24,0	p.Arg368Gln	401	9	NM_058216,2	c.1103G>A	missense	0,983	0,90(COSM981989)	Uncertain significance	0,00005
S18-04801	RB1	chr13:49039146	G/A	SNV	1			COSM6503563	17,2	62,0	p.Val742Ile	360	22	NM_000321,2	c.2224G>A	missense	0,998	0,99(COSM6503563)		0,00001
S18-04801	RB1	chr13:49033880	C/T	SNV	1			COSM6191574	5,1	39,0	p.His673Tyr	760	20	NM_000321,2	c.2017C>T	missense	1	0,98(COSM6191574)		0,00002
S18-04801	SMAD4	chr18:48591900	G/A	SNV	1			COSM24274	7,3	38,0	p.Asp355Asn	521	9	NM_005359,5	c.1063G>A	missense	1	0,99(COSM24274)		0,00001
S18-04801	SMAD4	chr18:48591817	C/T	SNV	1			COSM7591050	6,5	30,0	p.Ala327Val	461	9	NM_005359,5	c.980C>T	missense	0,994	0,99(COSM7591050)		0,00001
S18-04801	SMAD4	chr18:48581225	C/T	SNV	1			COSM9233304	8,4	10,0	p.His177Tyr	119	5	NM_005359,5	c.529C>T	missense	0,167	0,99(COSM9233304)		0,00036
S18-05065	APC	chr5:112178932	G/A	SNV	1	Deleterious	Loss-of-function	COSM7648785	5,2	32,0	p.Trp2547Ter	612	16	NM_000038,5	c.7641G>A	nonsense		0,95(COSM7648785)		0,00005
S18-05065	APC	chr5:112176278	G/T	SNV	1	Deleterious	Loss-of-function	COSM6475415	5,4	29,0	p.Glu1663Ter	539	16	NM_000038,5	c.4987G>T	nonsense		0,95(COSM6475415)	Pathogenic	0,00006
S18-05065	ARID2	chr12:46230691	C/T	SNV	1	Hotspot	Loss-of-function	COSM278971	8,3	35,0	p.Arg314Cys	420	8	NM_152641,3	c.940C>T	missense	1	0,98(COSM278971)		0,00001
S18-05065	ARID2	chr12:46287446	C/T	SNV	1	Deleterious	Loss-of-function	COSM1628616	6,4	32,0	p.Arg1769Ter	498	20	NM_152641,3	c.5305C>T	nonsense		0,87(COSM1628616)	Pathogenic	0,00001
S18-05065	ATM	chr11:108155043	G/A	SNV	1	Deleterious	Loss-of-function	COSM7409390	5,1	66,0	p.Trp1279Ter	1307	26	NM_000051,3	c.3836G>A	nonsense		0,99(COSM7409390)	Pathogenic	0,00001
S18-05065	ATM	chr11:108172385	C/T	SNV	1	Deleterious	Loss-of-function	COSM172204	8,0	59,0	p.Arg1730Ter	740	35	NM_000051,3	c.5188C>T	nonsense		0,85(COSM172204)	Pathogenic/Likely pathogenic	0,00001
S18-05065	CHEK2	chr22:29130427	G/A	SNV	1	Deleterious	Loss-of-function	COSM6962113	8,1	33,0	p.Arg95Ter	408	2	NM_007194,4	c.283C>T	nonsense		0,95(COSM6962113)	Pathogenic	0,00001
S18-05065	EGFR	chr7:55249166	G/A	SNV	1			COSM8511928	9,6	27,0	p.Ala822Thr	282	20	NM_005228,4	c.2464G>A	missense	1	0,97(COSM8511928)	Uncertain significance	0,00001
S18-05065	FGFR2	chr10:123298200	C/T	SNV	1			COSM6956507	7,4	47,0	p.Met218Ile	638	6	NM_000141,4	c.654G>A	missense	0,99	0,99(COSM6956507)		0,00001
S18-05065	KIT	chr4:55594093	C/T	SNV	1			COSM8503369	10,7	79,0	p.Pro627Ser	742	12	NM_000222,2	c.1879C>T	missense	0,995	0,79(COSM8503369)	Uncertain significance	0,00001
S18-05065	NF1	chr17:29654554	G/A	SNV	1			COSM3742170	5,1	97,0	p.Arg1769Gln	1895	38	NM_001042492,2	c.5306G>A	missense	0,977	0,99(COSM3742170)	Uncertain significance	0,00001
S18-05065	NF1	chr17:29562981	C/T	SNV	1	Deleterious	Loss-of-function	COSM1382104	8,7	27,0	p.Arg1306Ter	311	29	NM_001042492,2	c.3916C>T	nonsense		0,91(COSM1382104)	Pathogenic	0,00001
S18-05065	POLD1	chr19:50918759	G/A	SNV	1			COSM5741692	27,5	33,0	p.Asp877Asn	120	21	NM_001256849,1	c.2629G>A	missense	1	0,93(COSM5741692)	Uncertain significance	0,00001
S18-05065	RAD51C	chr17:56811555	G/A	SNV	1			COSM981989	5,5	36,0	p.Arg368Gln	654	9	NM_058216,2	c.1103G>A	missense	0,983	0,90(COSM981989)	Uncertain significance	0,00001
S18-05065	RB1	chr13:49039374	C/T	SNV	1	Deleterious	Loss-of-function	COSM23824	15,6	57,0	p.Arg787Ter	365	23	NM_000321,2	c.2359C>T	nonsense		0,88(COSM23824)	Pathogenic	0,00001
S18-05065	RB1	chr13:48936983	C/T	SNV	1	Deleterious	Loss-of-function	COSM878	7,6	47,0	p.Arg251Ter	621	8	NM_000321,2	c.751C>T	nonsense		0,82(COSM878)	Pathogenic	0,00001
S18-05065	RB1	chr13:49054143	G/A	SNV	1			COSM5610464	6,1	34,0	p.Arg908Gln	562	27	NM_000321,2	c.2723G>A	missense	1	0,99(COSM5610464)		0,00001
S18-05065	SMAD4	chr18:48603033	G/A	SNV	1			COSM3422225	5,7	34,0	p.Arg445Gln	596	11	NM_005359,5	c.1334G>A	missense	0,827	0,99(COSM3422225)		0,00001
S18-05065	TP53	chr17:7577552	C/T	SNV	1			COSM44129	7,2	39,0	p.Met243Ile	544	7	NM_000546,5	c.729G>A	missense	0,999	0,97(COSM44129)		0,00001
S18-06595	CDKN2A	chr9:21971120	G/A	SNV	1	Deleterious	Loss-of-function	COSM12475	29,7	593,0	p.Arg80Ter	1998	2	NM_001195132,1	c.238C>T	nonsense		0,88(COSM12475)	Likely pathogenic, risk factor	0,00001
S18-06595	KRAS	chr12:25398284	CC/TC	SNV	1	Hotspot	Gain-of-function	COSM521	14,4	63,0	p.Gly12Asp	437	2	NM_033360,3	c.35G>A	missense	0,517	0,98(COSM521)	Pathogenic	0,00001
S18-06595	TP53	chr17:7577120	C/T	SNV	1	Hotspot	Loss-of-function	COSM10660	26,2	523,0	p.Arg273His	2000	8	NM_000546,5	c.818G>A	missense	0,901	1,00(COSM1,000660)	Pathogenic/Likely pathogenic	0,00001
S18-06950	KRAS	chr12:25398284	CC/TC	SNV	1	Hotspot	Gain-of-function	COSM521	32,6	215,0	p.Gly12Asp	659	2	NM_033360,3	c.35G>A	missense	0,517	0,98(COSM521)	Pathogenic	0,00001
S18-06950	TP53	chr17:7578443	A/C	SNV	1	Hotspot	Loss-of-function	COSM44216	28,1	100,0	p.Tyr163Asp	356	5	NM_000546,5	c.487T>G	missense	1	0,99(COSM44216)	Likely pathogenic	0,00001
S18-08122	MYC	chr8:128750803	C/G	SNV	1			COSM6209969	8,3	39,0	p.Leu114Val	472	2	NM_002467,5	c.340C>G	missense	0,855	0,96(COSM6209969)		0,00001
S18-08122	NF1	chr17:29548932	C/T	SNV	1			COSM6948528	5,2	20,0	p.Thr569Ile	388	15	NM_001042492,2	c.1706C>T	missense	0,999	0,97(COSM6948528)		0,00109
S18-08122	POLE	chr12:133220098	CCA/C	INDEL	2	Deleterious	Loss-of-function	COSM1745059	5,8	47,0	p.Val1446GlyfsTer3	813	34	NM_006231,3	c.4337_4338delTG	frameshiftDeletion		1,00(COSM1745059)	Uncertain significance	0,00001
S19-00024	ATM	chr11:108236087	G/A	SNV	1	Hotspot	Loss-of-function	COSM21626	6,4	29,0	p.Arg3008His	451	63	NM_000051,3	c.9023G>A	missense	1	0,99(COSM21626)	Likely pathogenic	0,00007
S19-00024	CTNNB1	chr3:41266152	G/A	SNV	1			COSM5718	5,6	27,0	p.Gly50Asp	481	3	NM_001904,3	c.149G>A	missense	0,002	0,98(COSM5718)		0,00005
S19-00024	EGFR	chr7:55221822	C/A	SNV	1	Hotspot	Gain-of-function	COSM21685	7,3	14,0	p.Ala289Asp	193	7	NM_005228,4	c.866C>A	missense	0,997	0,99(COSM21685)	Likely pathogenic	0,00104
S19-00024	FGFR2	chr10:123298225	C/T	SNV	1			COSM174986	6,5	27,0	p.Arg210Gln	418	6	NM_000141,4	c.629G>A	missense	1	1,00(COSM1,0074986)		0,00001
S19-00024	KRAS	chr12:25398284	CC/AC	SNV	1	Hotspot	Gain-of-function	COSM520	11,7	36,0	p.Gly12Val	307	2	NM_033360,3	c.35G>T	missense	0,999	0,98(COSM520)	Pathogenic	0,00001

ID	Genes	Locus	Genotype	Type	Length	Oncomine Variant Class	Oncomine Gene Class	Variant ID	% Frequency	Mutated Alleles	Amino Acid Change	Coverage	Exon	Transcript	Coding	Variant Effect	PolyPhen	FATHMM	ClinVar	p-value
S19-00024	MYC	chr8:128750803	C/G	SNV	1			COSM6209969	8,9	20,0	p.Leu114Val	226	2	NM_002467,5	c.340C>G	missense	0,855	0,96(COSM6209969)		0,00001
S19-00024	NF1	chr17:29553610	G/A	SNV	1			COSM6724959	5,3	64,0	p.Arg720Gln	1205	18	NM_001042492,2	c.2159G>A	missense	0,938	0,96(COSM6724959)	Uncertain significance	0,00001
S19-00024	NF1	chr17:29560175	C/T	SNV	1	Deleterious	Loss-of-function	COSM5049808	5,9	18,0	p.Gln1218Ter	307	27	NM_001042492,2	c.3652C>T	nonsense		0,98(COSM5049808)		0,00041
S19-00024	NF1	chr17:29684085	C/T	SNV	1	Deleterious	Loss-of-function	COSM5535345	5,4	10,0	p.Gln2616Ter	184	53	NM_001042492,2	c.7846C>T	nonsense		0,98(COSM5535345)		0,00846
S19-00024	PIK3CA	chr3:178937060	G/T	SNV	1			COSM8034249	7,7	13,0	p.Ala581Ser	169	11	NM_006218,3	c.1741G>T	missense	0,002	0,98(COSM8034249)		0,00015
S19-00024	POLE	chr12:133218912	C/T	SNV	1			COSM6071973	8,9	34,0	p.Arg1675His	384	38	NM_006231,3	c.5024G>A	missense	1	0,99(COSM6071973)		0,00001
S19-00024	POLE	chr12:133253140	C/T	SNV	1			COSM5031038	10,9	27,0	p.Asp301Asn	248	9	NM_006231,3	c.901G>A	missense	0,997	0,99(COSM5031038)	Uncertain significance	0,00001
S19-00024	POLE	chr12:133202801	G/A	SNV	1	Deleterious	Loss-of-function	COSM4503587	6,8	24,0	p.Arg2145Ter	355	46	NM_006231,3	c.6433C>T	nonsense		0,92(COSM4503587)	Uncertain significance	0,00001
S19-00024	RAD51C	chr17:56787223	C/T	SNV	1	Deleterious	Loss-of-function	COSM212455	7,1	32,0	p.Arg237Ter	454	5	NM_058216,2	c.709C>T	nonsense		0,78(COSM212455)	Pathogenic	0,00001
S19-00024	RB1	chr13:49050869	G/A	SNV	1			COSM5945309	5,1	41,0	p.Met851Ile	811	25	NM_000321,2	c.2553G>A	missense	0,711	0,95(COSM5945309)		0,00001
S19-00024	SMAD4	chr18:48604809	C/T	SNV	1			COSM6985223	5,3	17,0	p.Pro544Leu	320	12	NM_005359,5	c.1631C>T	missense	0,154	0,99(COSM6985223)		0,0016
S19-00024	TP53	chr17:7577085	C/T	SNV	1	Hotspot	Loss-of-function	COSM10722	20,1	195,0	p.Glu285Lys	970	8	NM_000546,5	c.853G>A	missense	1	0,99(COSM10722)	Pathogenic/Likely pathogenic, dr	0,00001
S19-00024	TP53	chr17:7577552	C/T	SNV	1			COSM44129	6,0	20,0	p.Met243Ile	335	7	NM_000546,5	c.729G>A	missense	0,999	0,97(COSM44129)		0,00017
S19-00643	KIT	chr4:55593599	TGG/TGA	SNV	1			COSM1231	5,2	14,0	p.Trp557Ter	267	11	NM_000222,2	c.1671G>A	nonsense		0,98(COSM1231)		0,01755
S19-00856	KRAS	chr12:25380275	TT/TC	SNV	1	Hotspot	Gain-of-function	COSM552	21,2	342,0	p.Gln61Arg	1614	3	NM_033360,3	c.182A>G	missense	0,026	0,98(COSM552)	Pathogenic	0,00001
S19-00856	MYC	chr8:128750803	C/G	SNV	1			COSM6209969	7,3	41,0	p.Leu114Val	562	2	NM_002467,5	c.340C>G	missense	0,855	0,96(COSM6209969)		0,00001
S19-00856	SMAD4	chr18:48591901	A/T	SNV	1			COSM3388466	15,0	129,0	p.Asp355Val	860	9	NM_005359,5	c.1064A>T	missense	1	0,99(COSM3388466)		0,00001
S19-00856	TP53	chr17:7577559	G/A	SNV	1	Hotspot	Loss-of-function	COSM10812	19,9	227,0	p.Ser241Phe	1143	7	NM_000546,5	c.722C>T	missense	1	0,94(COSM10812)	Likely pathogenic	0,00001
S19-01257	CDKN2A	chr9:21971120	G/A	SNV	1	Deleterious	Loss-of-function	COSM12475	13,5	233,9	p.Arg80Ter	1729	2	NM_001195132,1	c.238C>T	nonsense		0,88(COSM12475)	Likely pathogenic, risk factor	0,00001
S19-01257	KRAS	chr12:25398284	CC/TC	SNV	1	Hotspot	Gain-of-function	COSM521	7,5	37,0	p.Gly12Asp	496	2	NM_033360,3	c.35G>A	missense	0,517	0,98(COSM521)	Pathogenic	0,00001
S19-01257	POLE	chr12:133220098	CCA/C	INDEL	2	Deleterious	Loss-of-function	COSM1745059	5,1	50,0	p.Val1446GlyfsTer3	976	34	NM_006231,3	c.4337_4338delTG	frameshiftDeletion		COSM1745059	Uncertain significance	0,00014
S19-01257	TP53	chr17:7578268	A/C	SNV	1	Hotspot	Loss-of-function	COSM44571	13,3	64,0	p.Leu194Arg	480	6	NM_000546,5	c.581T>G	missense	1	1,00(COSM44571,00)	Uncertain significance	0,00001
S19-02445	MYC	chr8:128750803	C/G	SNV	1			COSM6209969	8,2	45,0	p.Leu114Val	549	2	NM_002467,5	c.340C>G	missense	0,855	0,96(COSM6209969)		0,00001
S19-03810	CDKN2A	chr9:21971120	G/A	SNV	1	Deleterious	Loss-of-function	COSM12475	18,6	372,0	p.Arg80Ter	1998	2	NM_001195132,1	c.238C>T	nonsense		0,88(COSM12475)	Likely pathogenic, risk factor	0,00001
S19-03810	MYC	chr8:128750803	C/G	SNV	1			COSM6209969	5,6	53,0	p.Leu114Val	954	2	NM_002467,5	c.340C>G	missense	0,855	0,96(COSM6209969)		0,00001
S19-03810	NRAS	chr1:115256529	TG/CG	SNV	1	Hotspot	Gain-of-function	COSM584	25,8	348,1	p.Gln61Arg	1348	3	NM_002524,4	c.182A>G	missense	0,214	0,99(COSM584)	Pathogenic	0,00001
S19-03810	TP53	chr17:7578448	G/C	SNV	1			COSM46279	13,5	269,1	p.Ala161Gly	1999	5	NM_000546,5	c.482C>G	missense	0,999	1,00(COSM46279)		0,00001
S19-04576	CDKN2A	chr9:21971156	C/T	SNV	1			COSM13604	6,5	26,0	p.Ala68Thr	402	2	NM_001195132,1	c.202G>A	missense	1	0,97(COSM13604)		0,00001
S19-04576	MYC	chr8:128750803	C/G	SNV	1			COSM6209969	6,7	84,0	p.Leu114Val	1256	2	NM_002467,5	c.340C>G	missense	0,855	0,96(COSM6209969)		0,00001
S19-04576	POLE	chr12:133209315	G/A	SNV	1			COSM6943211	5,3	40,0	p.Pro2024Leu	753	44	NM_006231,3	c.6071C>T	missense	0,942	0,99(COSM6943211)		0,00001
S19-05740	KRAS	chr12:25398284	CC/TC	SNV	1	Hotspot	Gain-of-function	COSM521	6,0	28,0	p.Gly12Asp	469	2	NM_033360,3	c.35G>A	missense	0,517	0,98(COSM521)	Pathogenic	0,00029
S19-05740	SMAD4	chr18:48591888	G/C	SNV	1	Hotspot	Loss-of-function	COSM14135	12,3	120,0	p.Asp351His	973	9	NM_005359,5	c.1051G>C	missense	1	0,99(COSM14135)	Pathogenic	0,00001
S19-05740	TP53	chr17:7578503	C/T	SNV	1			COSM43878	12,4	130,0	p.Val143Met	1050	5	NM_000546,5	c.427G>A	missense	1	0,83(COSM43878)	Uncertain significance	0,00001
S19-08372	KRAS	chr12:25398261	T/C	SNV	1			COSM87289	16,4	57,0	p.Thr20Ala	347	2	NM_033360,3	c.58A>G	missense	1	0,98(COSM87289)		0,00001
S19-08372	KRAS	chr12:25398284	CC/TC	SNV	1	Hotspot	Gain-of-function	COSM521	15,7	54,0	p.Gly12Asp	344	2	NM_033360,3	c.35G>A	missense	0,517	0,98(COSM521)	Pathogenic	0,00001
S19-08372	MYC	chr8:128750803	C/G	SNV	1			COSM6209969	6,2	31,0	p.Leu114Val	502	2	NM_002467,5	c.340C>G	missense	0,855	0,96(COSM6209969)		0,00001
S19-08372	TP53	chr17:7577511	A/G	SNV	1			COSM43842	13,3	87,0	p.Leu257Pro	652	7	NM_000546,5	c.770T>C	missense	1	0,99(COSM43842)	Uncertain significance	0,00001
S20-00190	KRAS	chr12:25398284	CC/TC	SNV	1	Hotspot	Gain-of-function	COSM521	18,6	186,0	p.Gly12Asp	1002	2	NM_033360,3	c.35G>A	missense	0,517	0,98(COSM521)	Pathogenic	0,00001
S20-00190	MYC	chr8:128750803	C/G	SNV	1			COSM6209969	6,1	111,0	p.Leu114Val	1825	2	NM_002467,5	c.340C>G	missense	0,855	0,96(COSM6209969)		0,00001
S20-00190	TP53	chr17:7577548	C/T	SNV	1	Hotspot	Loss-of-function	COSM6932	25,1	502,0	p.Gly245Ser	2000	7	NM_000546,5	c.733G>A	missense	1	0,99(COSM6932)	Pathogenic	0,00001
S20-00355	MYC	chr8:128750803	C/G	SNV	1			COSM6209969	6,2	51,0	p.Leu114Val	821	2	NM_002467,5	c.340C>G	missense	0,855	0,96(COSM6209969)		0,00001
